# *GhSBI1*, a *CUP‐SHAPED COTYLEDON 2* homologue, modulates branch internode elongation in cotton

**DOI:** 10.1111/pbi.14439

**Published:** 2024-07-26

**Authors:** Weiping Zhong, Lanxin Wu, Yan Li, Xiaxuan Li, Junyi Wang, Jingwen Pan, Shouhong Zhu, Shentao Fang, Jinbo Yao, Yongshan Zhang, Wei Chen

**Affiliations:** ^1^ State Key Laboratory of Cotton Bio‐breeding and Integrated Utilization Institute of Cotton Research, Chinese Academy of Agricultural Sciences Anyang China; ^2^ Western Agricultural Research Center, Chinese Academy of Agricultural Sciences Changji Xinjiang China; ^3^ Zhengzhou Research Base, National Key Laboratory of Cotton Bio‐breeding and Integrated Utilization, School of Agricultural Sciences Zhengzhou University Zhengzhou China

**Keywords:** cotton (*Gossypium hirsutum*), internode elongation, *CUP‐SHAPED COTYLEDON 2* (*CUC2*), ghr‐miR164, gibberellic acid (GA)

## Abstract

Branch length is an important plant architecture trait in cotton (*Gossypium*) breeding. Development of cultivars with short branch has been proposed as a main object to enhance cotton yield potential, because they are suitable for high planting density. Here, we report the molecular cloning and characterization of a semi‐dominant quantitative trait locus, *Short Branch Internode 1*(*GhSBI1*), which encodes a NAC transcription factor homologous to CUP‐SHAPED COTYLEDON 2 (CUC2) and is regulated by microRNA ghr‐miR164. We demonstrate that a point mutation found in *sbi1* mutants perturbs ghr‐miR164‐directed regulation of *GhSBI1*, resulting in an increased expression level of *GhSBI1*. The *sbi1* mutant was sensitive to exogenous gibberellic acid (GA) treatments. Overexpression of *GhSBI1* inhibited branch internode elongation and led to the decreased levels of bioactive GAs. In addition, gene knockout analysis showed that *GhSBI1* is required for the maintenance of the boundaries of multiple tissues in cotton. Transcriptome analysis revealed that overexpression of *GhSBI1* affects the expression of plant hormone signalling‐, axillary meristems initiation‐, and abiotic stress response‐related genes. *GhSBI1* interacted with GAIs, the DELLA repressors of GA signalling. *GhSBI1* represses expression of GA signalling‐ and cell elongation‐related genes by directly targeting their promoters. Our work thus provides new insights into the molecular mechanisms for branch length and paves the way for the development of elite cultivars with suitable plant architecture in cotton.

## Introduction

Cotton (*Gossypium*) is one of the most important textile crops in the world, and higher fibre yield still remains a major breeding objective for modern cotton breeders (Paterson *et al*., 2012). A favourable plant architecture that accommodates high‐density planting is a highly desirable trait for commercial cotton cultivars due to its potential to maximize yield per unit area (Yao *et al*., 2016). However, domesticated from tree‐like wild species (Wendel and Grover, 2015), most currently cultivated cotton cultivars typically reach a height of 1.5 m or more without chemical sprays, with spreading and indeterminate branches. This architecture is susceptible to lodging and not suitable for mechanical field management and harvesting. Extensive efforts have therefore been made to breed cotton cultivars with a more compact canopy structure, typically characterized by shortened branches, narrow branch angles, shortened petioles, and upright leaves.

Cultivated cotton typically has a compound branching architecture (Mauney, 1984; McGarry *et al*., 2016). The main shoot of cotton is a monopodial stem that exhibits indeterminate and vegetative growth with continuous generation of leaves and branches. They are spirally arranged on the main shoot with a three‐eighths phyllotaxy. Two types of axillary branches are differentiated on the main shoot. The basal 5 ~ 8 branches, called vegetative branches (VB), are monopodial and show an indeterminate growth habit, just like the main shoot. The distal fruit branches, called fruiting branches (FB), have a sympodial inflorescence structure. Each sympodial unit of the FB consists of an internode, a terminal flower, and a subtending leaf, and one of the axillary buds in the axil of the leaf develops into the next sympodial unit. Consistent with the annual growth habit of cultivated cotton, the elongation potential of the internode usually decreases for the upper FBs on the main shoot or the later sympodial units of the same FB, forming a pyramidal plant structure. The length of internodes in branches determines to a large extent the canopy structure of cotton.

In plants, organ boundaries serve to separate lateral organs such as branches, leaves, or flowers from the shoot apical meristem (SAM) or adjacent organs. Several genes have been reported to be involved in the patterning and maintenance of organ boundaries (Aida *et al*., 1999; Aida and Tasaka, 2006; Barton and Poethig, 1993; Laux *et al*., 1996; Mayer *et al*., 1998). A prominent class among these genes belongs to the NAC (NAM/ATAF1,2/CUC) transcription factor family, which includes the NO APICAL MERISTEM (NAM) gene in Petunia, the CUPULIFORMIS (CUP) gene in Antirrhinum, and the CUP‐SHAPED COTYLEDON genes (CUC1, CUC2, and CUC3) in Arabidopsis (Aida *et al*., 1997; Hibara *et al*., 2006; Souer *et al*., 1996; Weir *et al*., 2004). The mutants of these NAC genes show fusion of cotyledons and some other tissues (such as stem, sepal, and stamen) and defects in shoot apical meristem (SAM) formation. A recent study has shown that the repression of *CUC2*/*CUC3* is required for de novo stem cell establishment (Nicolas *et al*., 2022). In addition, *CUC2* and *CUC3* have been identified as key regulators of leaf margin development (Kierzkowski *et al*., 2019; Maugarny‐Cales *et al*., 2019; Serra and Perrot‐Rechenmann, 2020). *CUC1*, *CUC2*, and four other Arabidopsis NAC genes (*NAC1*, *ORE1*, *NAC4*, and *AtNAC5*) are targeted and negatively regulated by the microRNA miR164 (Aida and Tasaka, 2006).

The CUC genes and their homologues were also found to alter plant architecture by affecting leaf or inflorescence morphology. Expression of a miR164‐resistant *CUC1* gene resulted in broader rosette leaves with shortened petioles (Mallory *et al*., 2004), and the expression of a miR164‐resistant *CUC2* gene (CUC2g‐m4) resulted in enhanced leaf serration and abnormal phyllotactic patterns (Nikovics *et al*., 2006). In addition, some CUC2g‐m4 lines with strong phenotypes showed a severe reduction in inflorescence internode elongation. A recent study showed that *CUC2*/*CUC3* promotes axillary meristem initiation by activating a ubiquitin‐dependent peptidase gene *DA1* (Li *et al*., 2020a). In rice, overexpression of *OsNAC2* (*OMTN2*), a homologue of *CUC1*/*CUC2*, resulted in more tillers by promoting tiller bud outgrowth but reduced plant height by shortening stem internodes and panicles (Chen *et al*., 2015; Mao *et al*., 2007). In addition, *OsNAC2* regulates abiotic stress responses by repressing ABA‐dependent stress‐related genes, and it accelerates leaf senescence by upregulating the expression of ABA biosynthesis genes (Mao *et al*., 2017). Conversely, knockdown of *OsNAC2* increased the primary root length and the number of crown roots (Mao *et al*., 2020). Interestingly, in another report, the overexpression of miR164‐resistant *OsNAC2* resulted in longer main panicles, more tillers, thicker stems, higher leaf area, and longer root systems, whereas knockdown of *OsNAC2* resulted in shorter panicles and fewer branches (Jiang *et al*., 2018). Similarly, overexpression (upregulated transcript levels due to miR164 resistance) and knockout of *OsNAM* (*OMTN5*) both resulted in dwarf plant architecture (Chang *et al*., 2021). Knockout of *OsCUC1*, another homologue of *CUC1*/*CUC2*, resulted in multiple defects including dwarf plant architecture and male sterility (Wang *et al*., 2021a). However, little is known about the underlying molecular mechanism by which CUC genes/homologues influence plant architecture.

In this study, we successfully identified the causal gene (named *GhSBI1*) for a short‐branch‐internode *1*(*sbi1*) mutant in cotton by map‐based cloning. *GhSBI1* encodes a homologue of *CUC2*, and the *sbi1* mutant has a single‐nucleotide substitution that causes a reduced cleavage efficiency of this gene by microRNA ghr‐miR164. We also found that GhSBI1 physically interacts with GhGAI, the key repressor of the GA signalling pathway. In addition, we also found that *GhSBI1* represses the expression of certain hormone biosynthesis/signalling or cell expansion genes by binding directly to their promoters. The identification of *GhSBI1* not only improves our understanding of the genetic regulation of cotton plant architecture but also presents a novel target for cotton breeding improvement strategies.

## Results

### Phenotypic characterization and inheritance of *sbi1*

**Figure 1:**
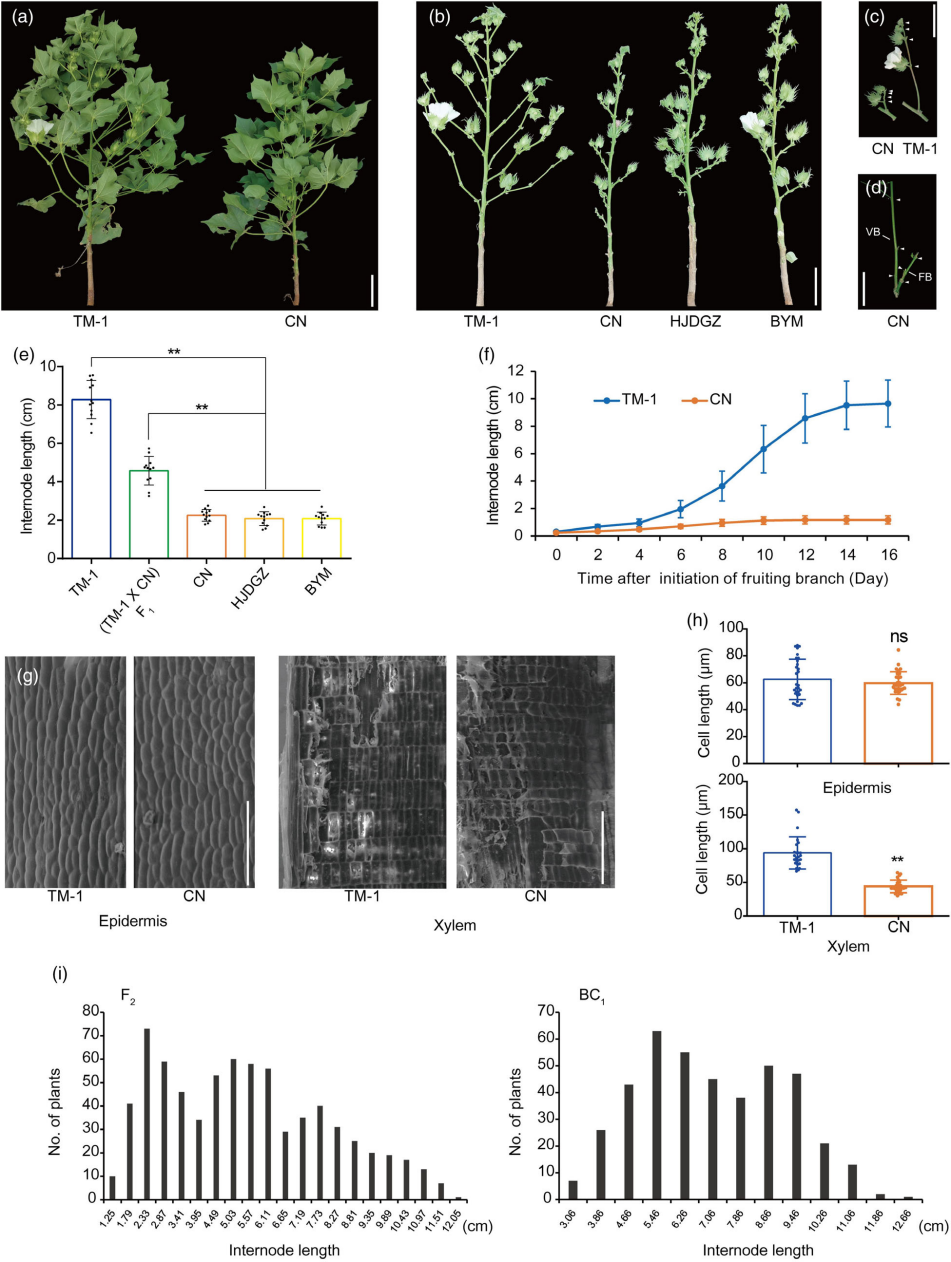
Phenotypic characterization of the *sbi1* mutant. (a) Phenotypes of TM‐1 (wild‐type, WT) and CN (*sbi1*) plants. Scale bar = 10 cm. (b) Defoliated plants. (c) Fruiting branch. (d) Vegetative branch (VB) and fruiting branch (FB) in the same leaf axil of CN. White triangles in c and d indicate nodes of branches. (e) Comparison of internode lengths of FB between WT and *sbi1* mutants. (f) Comparison of internode lengths of FB across the different growth stages in WT and CN. (g) Scanning electron microscopy of the longitudinal sections of the first internode of mature FB (no longer stretching). (h) Comparison of the average cell length. (i) Frequency distribution of the internode lengths in the F_2_ and BC_1_ populations derived from the cross TM‐1 × CN. Data are presented as means ± standard deviation; *P*‐values are determined using Student's *t*‐test (**P < 0.01, ns *P* > 0.05). Scale bars = 10 cm in (a)–(d) and 200 μm in (g).

Chuannong72318 (CN) was selected from a collection of 30 short fruiting branch lines. It consistently exhibited extremely short fruiting branch (FB) internode (<3 cm) in multiple growing reasons compared with normal cotton lines (internode >8 cm) (Figure 1a–e). Interestingly, the internode of vegetative branches (VB) in CN, like the main shoot, was relatively normal, even when differentiated in the same leaf axils with fruiting branches (Figure 1d). The dynamic study of length showed that internode elongation was severely inhibited in CN at the rapid elongation stage (6–12 days after initiation) (Figure 1f). The cell length of the epidermis showed no difference between CN and TM‐1, but for the xylem it was significantly reduced in CN (Figure 1g,h). When CN was crossed with TM‐1 (a genetic and genomic standard line for upland cotton), the F_1_ plants had an internode midway between the two parents (Figure 1c). In the F_2_ and BC_1_ populations of this cross, internode length showed a trimodal and bimodal distribution, respectively (Figure 1i). The chi‐square test showed that the short‐internode (≤3 cm), intermediate (3–7 cm), and long‐internode (≥7 cm) plants in the F_2_ population were in a ratio of 1:2:1 (χ^2^ = 1.46, *P* > 0.05), while in the BC_1_ population the intermediate and long‐internode plants were in a ratio of 1:1 (χ^2^ = 0.03, *P* > 0.05). These results suggest that the short internode phenotype of CN is controlled by a single semi‐dominant locus (named *GhSBI1*, *SHORT BRANCH INTERNODE 1*).

Most of the plant architecture mutants result from changes in phytohormone biosynthesis or signalling. To explore the role of phytohormones in the *sbi1* mutant, exogenous phytohormone treatments were conducted to TM‐1 and CN. The internode elongation of CN was sensitive to exogenous GA3. For the first and second internodes of FB, exogenous GA3 application could rescue the length of CN comparable to that of TM‐1, though TM‐1 was insensitive (Figure S1). For exogenous IAA and BR treatments, no change was found for the FB length in both TM‐1 and CN (data not shown).

### Map‐based cloning of the *GhSBI1* locus

**Figure 2:**
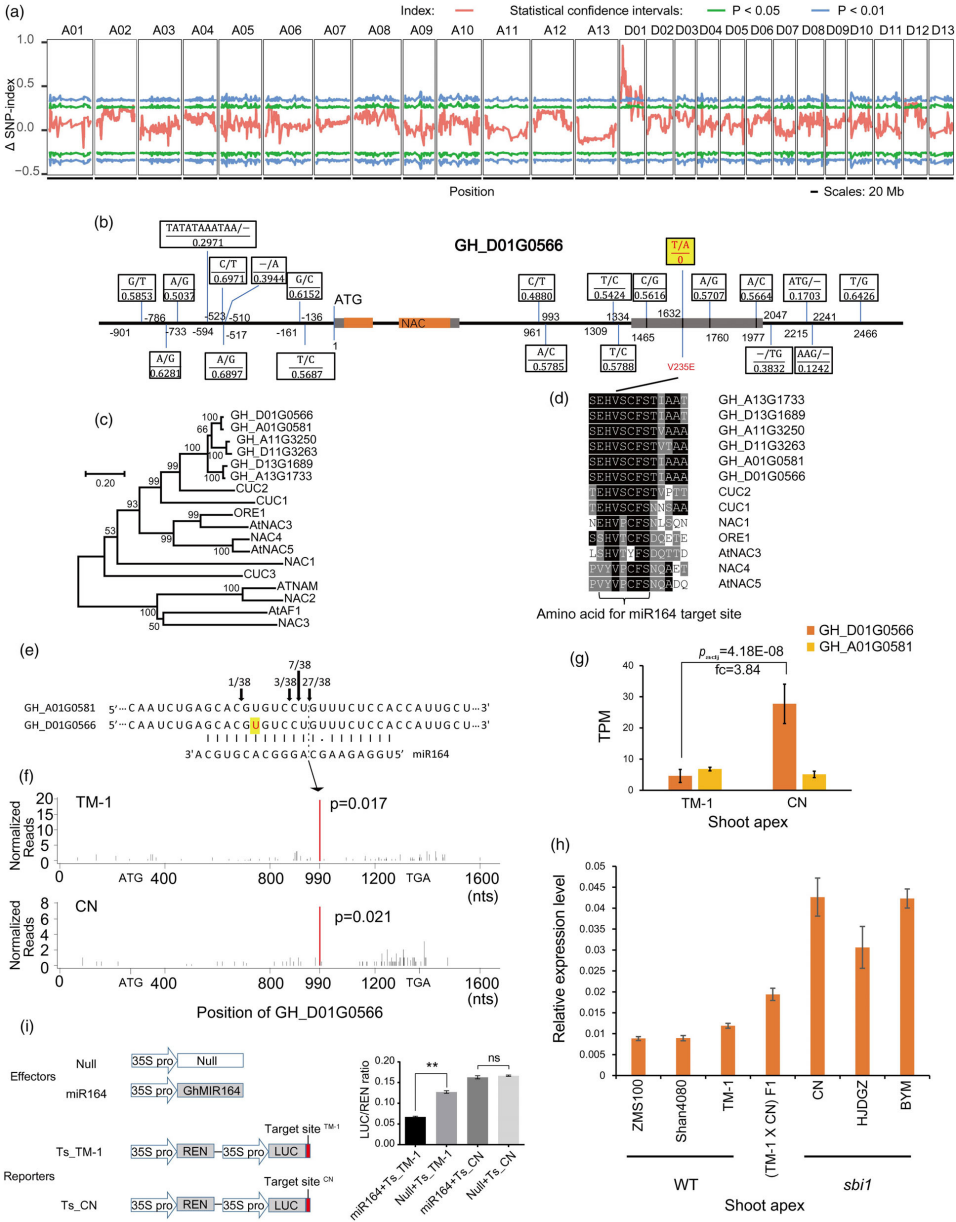
Map‐based cloning of *GhSBI1* and causal variation analyses. (a) *GhSBI1* was mapped on chromosome D01 by QTL‐Seq analysis. Red lines indicate average Δ (SNP‐index) values in sliding windows (1.0‐Mb width with 100 kb increments); green and blue lines indicate statistical confidence intervals under the null hypothesis of no causal gene (green, *P* < 0.05; blue, *P* < 0.01). (b) Summary of variations of *GH_D01G0566* in CN. Numbers under genotypes indicate genotype frequencies in cotton germplasm. Grey boxes indicate exons. (c) Neighbour‐joining phylogenetic tree of NAC transcription factors from cotton and Arabidopsis. Bootstrap values (%) based on 1000 replicates are indicated next to the nodes. (d) Sequence alignment of miR164 targeting sites. *AtNAC3* could not be targeted by miR164. (e) Cleavage sites in *GH_D01G0566*/*GH_A01G0581* obtained from RLM‐RACE experiments. Red letter on yellow background indicates the position of T6400827A. (f) Cleavage positions predicted from degradome sequencing. Red peak indicates the ghr‐miR164 cleavage site position. (g) Transcript abundance of *GH_D01G0566*/*GH_A01G0581* in RNA‐seq. *P*_adj_, adjusted *P*‐value. fc, fold change. (h) qRT‐PCR analysis of *GH_D01G0566* expression in WT and *sbi1* cotton. (i) Verification of the splicing effect of cotton ghr‐miR164 on its target site. ghr‐MIR164 indicates the precursor of ghr‐miR164. Data are presented as means ± standard deviation (*n* = 3); *P*‐values are determined using Student's *t*‐test (** *P* < 0.01, ns *P* > 0.05).

A bulked segregant analysis was used to map *GhSBI1*. The Δ (SNP‐index) calculation revealed the presence of a major effect locus on chromosome D01 (Figure 2a), and the peak value of the Δ (SNP‐index) was in the interval 5.4–6.8 Mb (Figure S2a). To narrow down the position of *GhSBI1*, all 728 F_2_ plants were genotyped with eight SNP markers from the mapping interval. Using classical quantitative trait locus (QTL) mapping method, the *GhSBI1* locus was mapped between the physical interval 6 350 303–6 498 773 bp (Figure S2b), explaining 62.1% of the phenotypic variation for FB internode length. These markers were also used to genotype another F_2_ population (1705 F_2_ plants) from the same cross in 2017. Combined with the phenotypic data of the F_2:3_ families of these recombinant F_2_ plants, the *GhSBI* locus was finally localized to a 95.5‐kb region (6350303–6 445 818 bp) (Figure S2c). This region contains three gene models in the TM‐1 reference genome (Figure S2d). *GH_D01G0566* encodes a NAC transcription factor homologous to *CUC2* in *Arabidopsis thaliana*. *GH_D01G0567* encodes a protein of unknown function, and no homologue is found outside *Gossypium* species. *GH_D01G0568* encodes a protein homologous to *EMB1027* (*EMBRYO DEFECTIVE 1027*) in *Arabidopsis thaliana*, which belongs to the class Ic arginyl‐tRNA synthetase. In the cotton public gene expression database of TM‐1 (http://cotton.zju.edu.cn/), *GH_D01G0566* was barely detected in all tissues examined except the torus and ovule, but no expression of *GH_D01G0567* and *GH_D01G0568* could be found in any of the tissues examined (Figure S2e).

Copy number variation (CNV) and structural variation (SV) analyses showed that there were no CNV or SV variations between the two parents in the final mapping interval. For CN, there were 307 single‐nucleotide polymorphisms (SNPs) and 45 insertion/deletions (Indels) in the interval when aligned to the TM‐1 reference genome (Table S1). Of these, 22 SNPs and 7 Indels were located at upstream (distance from start codon <1000 bp), exonic, intronic, and downstream (distance from stop codon <1000 bp) regions. In the public variation database (http://cotton.zju.edu.cn/), all these 29 variations were popular and showed no association with internode length in 1094 Gh and 77 Gb lines (most of them showed normal internode length like that of TM‐1), except for one SNP (T6400827A) located in the third exons of *GH_D01G0566*, which was missing in the database (Figure 2b). After testing this SNP in the collection of 30 short fruiting branch lines using DNA marker based on this mutation, two other lines, Huojiaduanguozhi (abbreviated as HJDGZ) and Bayimian (abbreviated as BYM), were also found to carry this mutation (Figure 1e; Figure S3). Sanger sequencing of the genomic DNA of *GH_D01G0566* revealed that there are two haplotypes (Type‐1 and Type‐2) in upland cotton and that TM‐1 and CN are in different haplotypes (Figure S3). CN/HJDGZ/BYM shared the Type‐1 haplotype with other normal fruiting branch lines (such as NDM8, San4080, and ZMS24), but the T6400827A SNP was specific to them.

This nucleotide substitution results in an amino acid change (V235E), but this change is located outside the NAC domain of this gene. More importantly, this SNP was located in the putative microRNA ghr‐miR164 targeting site (Pang *et al*., 2009; Ruan *et al*., 2009). In fact, besides *GH_D01G0566* and its counterpart on A01 (*GH_A01G0581*), there are two other pairs of *CUC2* homologues putatively targeted by ghr‐miR164 on A11/D11 and A13/D13 (Figure 2c). Sequence alignment showed that this ghr‐miR164 complementary site (encoding seven amino acids) is conserved among these miR164 target genes across *Arabidopsis* and cotton, especially for the third (V) and fifth to seventh (CFS) amino acids in it (Figure 2d). The nucleotide change in these conserved sites might disrupt the cleavage of this transcript by miR164 due to base mismatch, leading to alterations in gene expression.

The RNA ligase‐mediated rapid amplification of cDNA ends (RLM‐RACE) experiment conducted in TM‐1 revealed that the cleavage of *GH_D01G0566*/*GH_A01G0581* occurred at the ninth nucleotide from the 5′ end of ghr‐miR164, and this finding was further substantiated through degradome sequencing in both TM‐1 and CN (Figure 2e,f). Additionally, the number of reads at the cleavage site in CN was lower compared to that in TM‐1, suggesting that the nucleotide substitution (T6400827A) might reduce the efficiency of cleavage. We compared the transcript abundance of *GH_D01G0566* and *GH_A01G0581* between TM‐1 and CN using RNA‐seq. Notably, *GH_D01G0566* exhibited significantly higher expression in CN, whereas *GH_A01G0581* displayed no discernible difference with relatively low expression levels (Figure 2g). To further substantiate our hypothesis that this mutation in CN diminishes the cleavage efficiency, we initially compared the promoter activity between TM‐1 and CN. The promoter fragment upstream of the start codon of *GH_D01G0566* was isolated from both TM‐1 and CN (1156 and 1147 bp, respectively). An *Agrobacterium*‐mediated GUS transient assay in tobacco (*Nicotiana benthamiana*) leaves was employed to assess their activities. The GUS staining indicated comparable levels of GUS activity between the two promoter constructs, and qRT‐PCR analyses also revealed similar mRNA levels of the GUS gene in both constructs (Figure S4). Therefore, the differential expression of *GH_D01G0566* between TM‐1 and CN is unlikely to be attributed to variations in the promoter region. The expression level of *GH_D01G0566* was then further investigated in other cotton lines using quantitative reverse transcriptase polymerase chain reaction (qRT‐PCR). As expected, HJDGZ and BYM also showed much higher expression levels of *GH_D01G0566* compared to TM‐1 and two other normal internode lines (Figure 2h). In F_1_ of the TM‐1 × CN cross, the expression level of *GH_D01G0566* is intermediate between TM‐1 and CN. To further verify the splicing effect of ghr‐miR164 on *GH_D01G0566* and the attenuation of this effect by the T6400827A SNP, we performed a transient dual‐luciferase reporter assay in tobacco protoplasts (Figure 2i). In this assay, overexpression of ghr‐miR164 (effector) could significantly reduce the LUC activity (reporter) for the TM‐1‐type target site, indicating a true cleavage effect between ghr‐miR164 and the predicted target sequence. However, the CN‐type reporter was resistant to this cleavage, suggesting that this SNP weakened the cleavage effect.

Taken together, the map‐based cloning showed that *GH_D01G0566* was the best candidate for the *GhSBI1* gene and that an SNP in the ghr‐miR164 target site could reduce the efficiency of cleavage, leading to higher expression.

### The expression pattern of the *GhSBI1* gene

**Figure 3:**
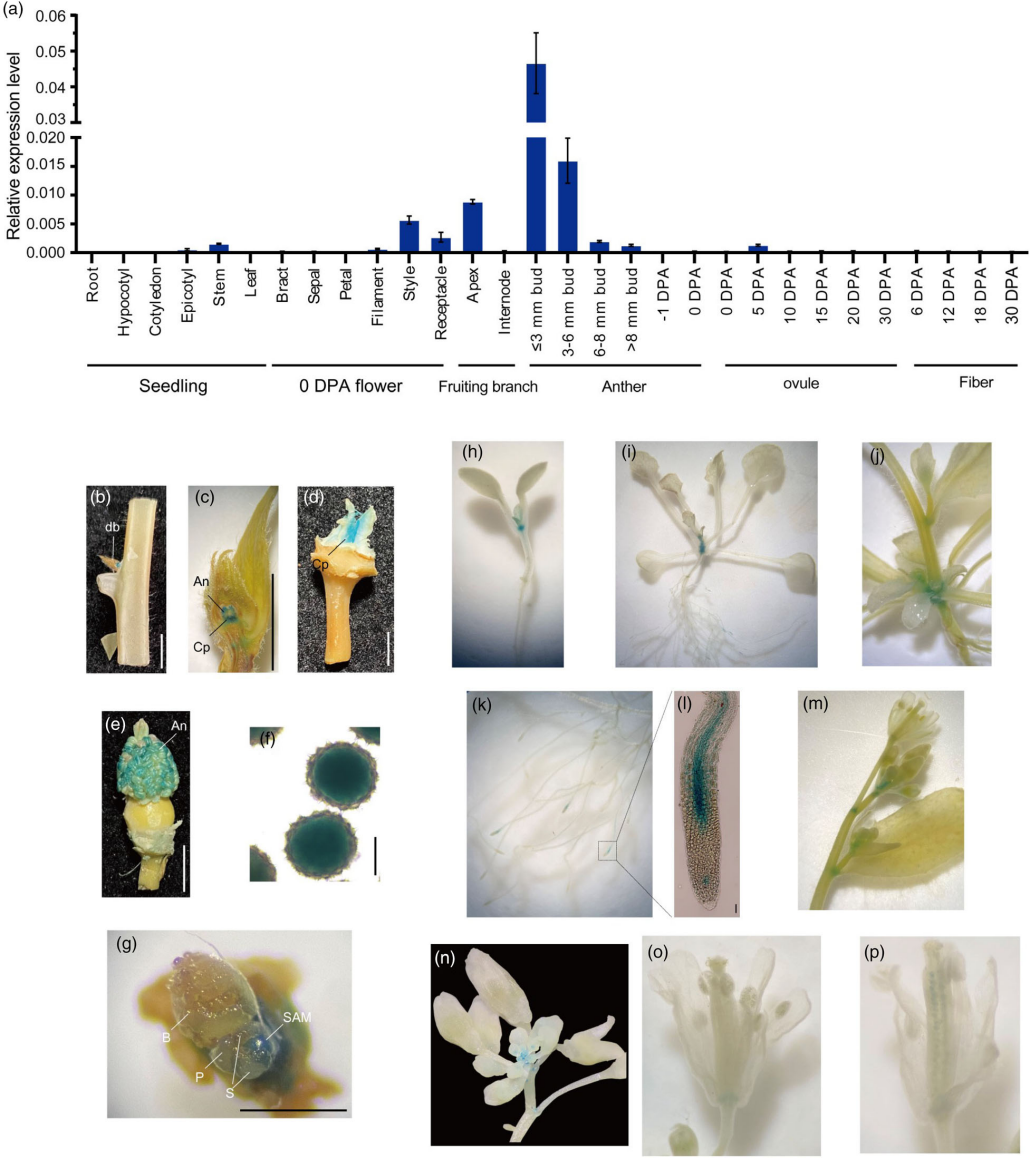
Expression pattern of *GhSBI1* in cotton and Arabidopsis. (a) Transcript levels in various tissues of TM‐1 obtained by qRT‐PCR. Error bars represent standard deviations of three biological replicates. Anthers were gathered at different developmental stages of buds according to the results of Wu *et al*. (2015). Numbers indicate lengths of buds. ≤ 3 mm: before ~ stage 4 of anther; 3–6 mm: at ~ stage 5–6; 6–8 mm: at ~ stage 7–8; >8 mm: later than ~ stage 9. DPA, day post anthesis. (b)–(g) Beta‐glucuronidase (GUS) staining of pGhSBI1:: GUS transgenic plants in cotton. GUS expression was detected in dormant buds (bd) of leaf axil (b), anther (An), and carpel (Cp) (c, d, e), pollen (f), and shoot apical meristem (SAM) (g). B, bract. P, prophyll. S, stipule. (h)–(p) GUS staining of pGhSBI1:: GUS transgenic plants in *Arabidopsis*. GUS expression was detected in the axils of rosette/cauline leaf (h, i, j), root tips (k, l), the base of branches/ inflorescences (m), flower buds (n), the base of filaments (o), and ovules after pollination (p).

A comprehensive survey of *GhSBI1* expression was carried out using qRT‐PCR across diverse tissues (Figure 3a). The highest expression levels were observed in the very early stages of anther development, with a subsequent decline as the anthers matured. Additionally, maximum expression occurred in the shoot apex, and moderate expression was detected in the style and receptacle. No expression was detected in ovules and fibres, except for 5‐DPA ovules. An 1147 bp promoter region of the *GhSBI1* from CN was cloned upstream of the GUS gene and transformed into YZ‐1. GUS activity was detected in the dormant axillary buds of the shoots, which usually consist of leaves, axillary meristems, unelongated internode, and sometimes flower primordia. Strong GUS activity was detected in carpels, developing anthers and shoot apical meristem (Figure 3b–g). Considering the distinct plant architectures of cotton and Arabidopsis, we also transformed this construct into Col‐0 Arabidopsis (Figure 3h–p). At the seedling stage, GUS activity was detected in the leaf axils of rosette leaves and root tips. In root tips, GUS activity was mainly present in the vascular cylinder of the elongation zone, with a relatively weak signal also detected in the dormant central zone. After reproductive transformation, GUS activity was also observed in stem leaf axils, early flower buds, the basal part of flower stalks, ovules after pollination, and the basal part of filaments. However, no GUS signal was detected in anthers or pollen.

### Overexpression of *GhSBI1* alters the architecture of cotton

To confirm that *GhSBI1* controls the internode length of cotton, a *GhSBI1* overexpression (OE) vector driven by the cauliflower mosaic virus (CaMV) 35S promoter was transformed into YZ‐1, a line with okra‐shaped leaves and normal internode length. Transgenic plants were detected using the diagnostic marker based on the SNP T6400827A. All 12 T_0_ transgenic plants highly expressing *GhSBI1* exhibited darker green leaves, wider leaf lamina, shorter FB internodes, and significantly reduced plant height (Figure S5). However, these plants also exhibited severe sterility, possibly due to abnormal pollen development caused by excessive expression of *GhSBI1* in the anther, and no mature seeds were obtained from these transgenic plants.

**Figure 4:**
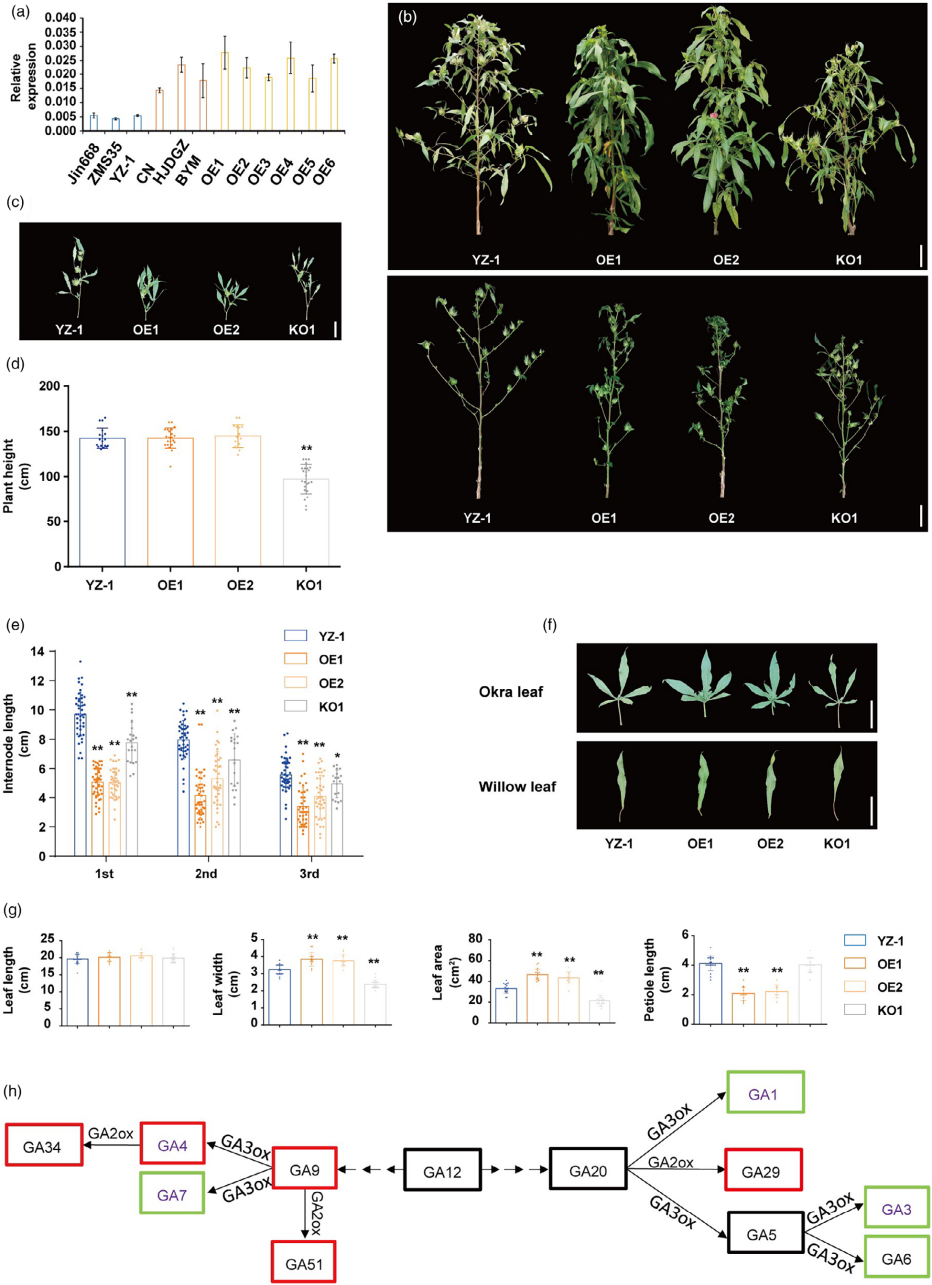
Phenotype analysis of *GhSBI1* overexpression (OE) and knockout (KO) lines. (a) Relative expression levels of *GhSBI1* detected using qRT‐PCR. Jin668, ZMS35, and YZ‐1 are normal‐branch lines. CN, HJDGZ, BYM are *sbi1* lines. OE1–OE6 are OE lines. Error bars represent standard deviations of three biological replicates. (b) Plant morphology of YZ‐1, OE, and KO plants. Upper, original plants; lower, defoliated plants. (c) Phenotype of fruiting branches. (d) Statistical data of plant height (*n* ≥ 16). (e) Statistical data of internode length. For each plant, the first to third internodes from fifth to eighth fruiting branches were measured (*n* ≥ 20). (f) Phenotype of leaves. (g) Statistical data of leaf size. The 15th leaf (willow type) on the main shoot was measured (*n* ≥ 12). (h) Content change of the indicated molecules from the GA biosynthesis pathway in the first internode at the rapid elongation stage from the fifth to tenth FB of YZ‐1 and OE1. Red and green indicate upregulated and downregulated, respectively. The molecules in purple are bioactive gibberellins. Significant differences are indicated by * (*P* < 0.05), ** (*P* < 0.01) (Student's t‐test). Scale bar = 10 cm.

To avoid this situation, we cloned the 5152‐bp genomic fragment spanning from 3093‐bp upstream of the translation start codon to 51‐bp downstream of the termination codon of *GhSBI1* from CN and introduced it into YZ‐1. Although severe sterility was still observed in some of the transgenic lines, we finally obtained six lines (OE1–OE6) with some degree of fertility, in which the expression levels of *GhSBI1* were comparable to those of CN/HJDGZ/BYM (Figure 4a). These six lines showed different degrees of inhibition of internode elongation compared to YZ‐1. For example, in OE1 and OE2, FB internode length was reduced by about 50% (Figure 4b–e). Unexpectedly, the leaves of these lines also showed a wider lamina, a shortened petiole, and a slightly darker coloration, like the above lines with excessive expression of *GhSBI1* caused by the 35S promoter (Figure 4f, g). Thus, the above results indicate that overexpression of *GhSBI1* inhibits FB internode and petiole elongation but promotes leaf expansion.

### Overexpression of *GhSBI1* altered endogenous hormone and flavonoid profiles

Quantification of the endogenous concentration of 94 compounds belonging to eight classes of phytohormones was performed for the first internode at the rapid elongation stage from the fifth to tenth FB of YZ‐1 and OE1 (Table S2; Figure 4h). For the auxin class, although the 3‐indole‐acetic acid (IAA) level was almost unchanged in OE1, some metabolic intermediates in the IAA biosynthetic pathway were increased, such as Indole‐3‐lactic acid (ILA), 3‐Indoleacrylic acid (IA) and Tryptamine (TRA). Two types of IAA metabolites that were found to be inactive in growth promotion, Methyl indole‐3‐acetate (MEIAA) and 2‐oxindole‐3‐acetic acid (OxIAA), were increased in OE1. In the cytokinin (CK) class, all tZ‐type compounds were upregulated in OE1. In the GA class, three bioactive GAs, GA1, GA3, and GA7, were downregulated in OE1, but another bioactive GA, GA4, was upregulated. Four inactive GAs, GA9, GA29, GA34, and GA51, were upregulated, indicating reduced biosynthesis of bioactive gibberellin or improved GA deactivation in OE1. The levels of abscisic acid (ABA) and 1‐Aminocyclopropanecarboxylic acid [ACC, ethylene (ETH) class] in OE1 were more than threefold increased. All jasmonate (JA) class compounds tested were significantly increased in OE1. In the strigolactone (SL) class, strigol was not detected and 5‐Deoxystrigol was increased in OE1.

High levels of flavonols have been shown to inhibit the growth of model plants (Falcone Ferreyra *et al*., 2012; Li and Zachgo, 2013; Tan *et al*., 2019; Yin *et al*., 2014). Therefore, we also investigated the concentration of 204 compounds belonging to 13 flavonoid classes such as flavonols, flavones, flavanols, flavanones, and isoflavanones (Table S3). Thirty compounds, including quercetin and kaemferol, showed a significant difference between YZ‐1 and OE1, and most of them were upregulated in OE1.

### Knockout of *GhSBI1* affects the development of multiple tissues

To further investigate the role of *GhSBI1* in cotton organ development, we used the CRISPR/Cas9 system to simultaneously knock out *GhSBI1_Dt* (*GH_D01G0566*) and *GhSBI1_At* (*GH_A01G0581*) in YZ‐1. We obtained six lines in which both *GhSBI1_At* and *GhSBI1_Dt* were completely knocked out (without native *GhSBI1* sequence) (Figure S6a). These lines exhibited high frequencies of fused cotyledons (~40%) (Figure S6b). Different levels of fusion result in various cotyledon shapes, including cup‐shaped, heart‐shaped, and twin cotyledons with a single fused petiole (see Figure S7a). In some plants, the cotyledons were closed (angle between cotyledons was less than 180°) but not fused. Most plants with cup‐shaped cotyledons failed to develop a shoot apical meristem and did not produce any leaves or shoots until the cotyledons senesced and died (Figure S7b). In some cup‐cotyledon and partially fused‐cotyledon plants, a single shoot developed at the base of the fused cotyledon petiole (Figure S7c). These shoots passively developed into main stems with normal branching and flowering, but the plants exhibited a degree of thinness. Plant height and branch internode length were reduced, and the leaves became smaller due to a constricted lamina (Figure 4b–g). In addition, fused stems were occasionally observed in these lines (Figure S7d).

Flower development is abnormal in these lines. Most of the flowers developed normal bracts, sepals, and petals (Figure S7e), but the inner whorls of these flowers developed abnormally. The styles were slightly thinner but longer, and the stigmas appeared to lose the pollen attraction (Figure S7f–i). Two adjacent filaments were usually fused in these flowers, and these fused filaments were shorter and wider than normal filaments (Figure S7j–l). Occasionally, some flowers had only two bracts (three in normal flowers) and the inner whorls of these flowers usually developed abnormally (Figure S7m–o). Some flowers lost all inner whorls and developed acicular tissue. Some flowers developed an additional bract whorl (usually smaller), and sometimes petals were missing. Severe sterility was observed in all knockout lines, and the boll setting rate is relatively low (<8%). Iodine–iodide potassium staining showed that pollen viability was reduced in these lines, and pollen released from mature anthers usually appeared sparse (Figure S7j).

Thus, the above results indicate that *GhSBI1* is required for the maintenance of the boundaries of multiple tissues in cotton, as its homologous genes do in other plants. In addition, the *GhSBI1* knockout affects leaf and flower development, and internode elongation was also affected to some extent.

### Transcriptome changes associated with overexpression of *GhSBI1*

**Figure 5:**
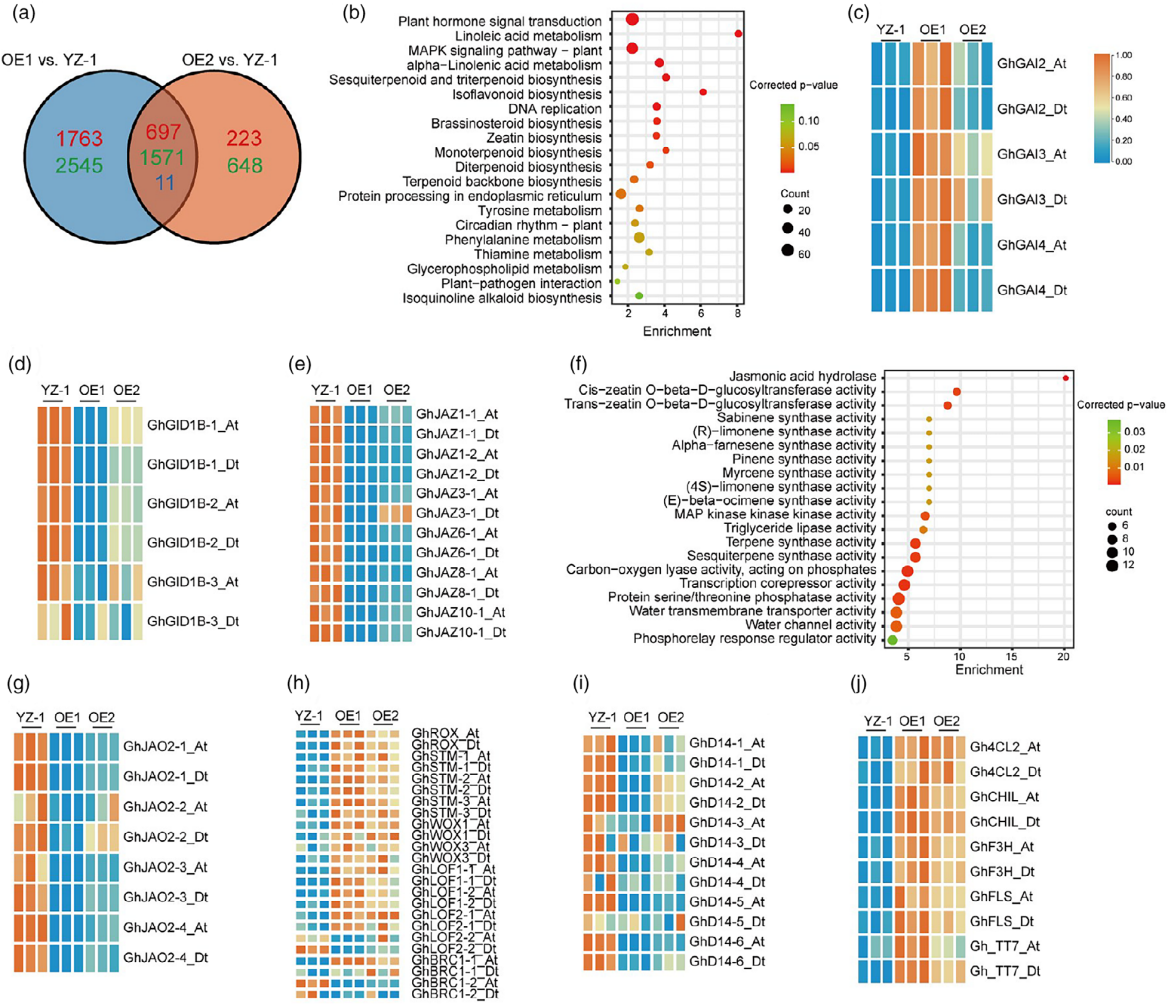
Transcriptome analysis of *GhSBI1*‐regulated genes. (a) Venn diagram of overlapping differentially expressed genes (DEGs) between OE1 vs. YZ‐1 and OE2 vs. YZ‐1. Red numbers indicate upregulated genes; green numbers indicate downregulated genes; the blue numbers indicate differentially regulated genes between OE1 vs. YZ‐1 and OE2 vs. YZ‐1. (b) Top 20 pathways in KEGG enrichment for the commonly regulated genes between the two OE lines. (c) Expression heatmap for *GhGAIs*. The expression values (transcripts per kilobase million, TPM) were normalized to the 0 to 1 scale by row. (d) Expression heatmap for *GhGID1Bs*. (e) Expression heatmap for *GhJAZs*. (f) Top 20 gene ontology (GO) terms in GO enrichments (molecular function class). (g) Expression heatmap for *GhJAO2s*. (h) Expression heatmap for the key genes related to axillary meristem initiation/formation. (i) Expression heatmap for *GhD14s*. (j) Expression heatmap for the key genes in flavonols biosynthesis.

We performed deep sequencing of the transcriptome (RNA sequencing [RNA‐seq]) to identify genes affected by *GhSBI1* in the two overexpression lines (OE1 and OE2) using the shoot apex of FB. 6587 and 3150 differentially expressed genes (DEGs) were identified in the OE1 versus YZ‐1 and OE2 versus YZ‐1 comparisons, respectively (Figure 5a). There were 2279 overlapping DEGs between OE1 and OE2, and almost all of them (2268 DEGs) were commonly regulated in both lines (both induced or both repressed) (Table S4). A KEGG enrichment analysis using the 2268 DEGs revealed a most significant enrichment in the plant hormone signalling pathway (Figure 5b). In the auxin signalling pathway, the overexpression lines exhibited upregulation of three Aux/IAA repressors, namely IAA4, IAA13, and IAA29. In the GA signalling pathway, three out of four homologues (*GhGAI2*, *GhGAI3*, and *GhGAI4*) of the Arabidopsis GAI, a key DELLA repressor of GA signalling, exhibited upregulation in the overexpression lines (Figure 5c). Concurrently, the GID1B genes (GA receptors), which are positive regulators of GA signalling, were downregulated in the overexpression lines (Figure 5d). In the ABA signalling pathway, 16 protein phosphatase 2C (PP2C) DEGs (PP2C25, PP2C49, PP2C56, PP2C63, PP2C72, and PP2CA), negative regulators of ABA signalling, were all downregulated in the OE lines. Given the elevated ABA levels observed in the overexpressing lines, we examined the nine‐CIS‐EPOXYCAROTENOID DIOXYGENASE (NCED) genes involved in ABA biosynthesis. However, none of these genes exhibited upregulation. In the JA signalling pathway, all jasmonate‐zim‐domain protein (JAZ) DEGs (JAZ1, JAZ3, JAZ6, JAZ8, and JAZ10), which act as repressors of JA signalling, were downregulated by *GhSBI1* overexpression (Figure 5e). In the gene ontology (GO) enrichment analysis, jasmonate hydrolase was the most enriched term in the molecular function class (Figure 5f). All jasmonate‐induced oxygenase 2(JAO2) DEGs were downregulated in the OE lines (Figure 5g). In Arabidopsis, JAO2 has been demonstrated to play a role in jasmonate oxidation and prevention of excessive JA accumulation (Caarls *et al*., 2017; Smirnova *et al*., 2017). Therefore, the elevated JA levels observed in the OE line could be attributed to a compromised jasmonate metabolism. All DEGs encoding ethylene‐responsive transcription factors (*ERF4*, *ERF5*, *ERF9*, and *ERF13*) were downregulated in the OE lines, except for *ERF1*.

Regarding the pivotal genes related to the initiation and formation of axillary meristems, *REGULATOR OF AXILLARY MERISTEM FORMATION* (*ROX*), *WUSCHEL RELATED HOMEOBOX 1* (*WOX1*), *WOX3*, *LATERAL ORGAN FUSION 1*(*LOF1*), and *SHOOT MERISTEMLESS* (*STM*) were found to be upregulated in the OE lines (Figure 5h). There were two cotton homologues for *BRANCHED 1*(*BRC1*) and *LOF2*, and one was upregulated while the other was downregulated. All homologues of *DWARF14* (*D14*), an essential receptor for strigolactone signalling, were downregulated in the overexpressing lines (Figure 5i).

In accordance with the higher level of flavonols, the key genes in flavonol biosynthesis were upregulated in the overexpression lines. These genes include *4‐coumarate‐CoA ligase 2*(*4CL2*), *Chalcone isomerase 3* (*CHIL*), *Flavanone‐3‐hydroxylase* (*F3H*), *Flavonol synthase*/*flavanone 3‐hydroxylase* (*FLS*), and *CYTOCHROME P450 75B1*/*TRANSPARENT TESTA 7* (*TT7*) (Figure 5j).

Interestingly, many stress‐related genes were found in DEGs, such as *ZINC FINGER OF ARABIDOPSIS THALIANA 10* (*ZAT10*) (Bittner *et al*., 2021; Mittler *et al*., 2006; Sakamoto *et al*., 2004), *WRKY DNA‐BINDING PROTEIN 33* (*WRKY33*) (Guo *et al*., 2022; Wang and Zhang, 2021), and *COLD‐REGULATED GENE 27* (*COR27*) (Li *et al*., 2016, 2020b; Mikkelsen and Thomashow, 2009; Wang *et al*., 2017; Zhu *et al*., 2020).

### GhSBI1 interacts with GhGAIs

**Figure 6:**
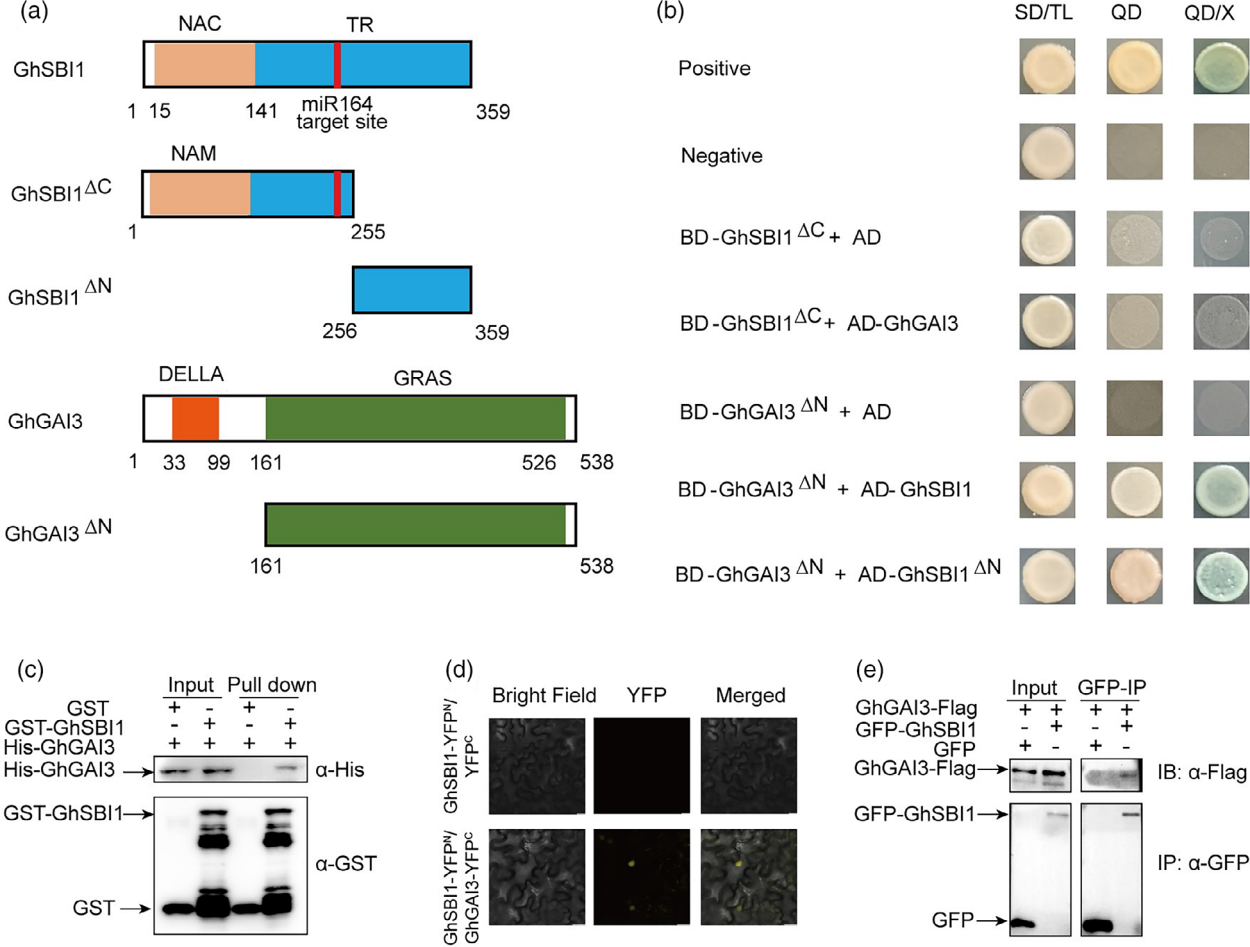
GhSBI1 physically interacts with GhGAI3. (a) Schematic diagram of different truncated versions of proteins used in yeast two‐hybrid assays. (b) Yeast two‐hybrid assays of the interactions between GhSBI1 and GhGAI3. SD/TL, double‐dropout medium; QD and QD/X, quadruple dropout medium without or with X‐α‐Gal. (c) In vitro pull‐down assays of GhSBI1–GhGAI3 interaction. (d) BiFC assay of GhSBI1–GhGAI3 interaction. Bar = 20 μm. (e) Detection of GhSBI1‐GhGAI3 interaction by co‐immunoprecipitation.

The *sbi1* mutant was sensitive to exogenous GA3, and *GhGAIs* were upregulated in the over‐expression lines. A recent study showed that *GhGAIs* regulated secondary cell wall (SCW) development, and knockdown of these GAI genes promoted stem elongation of cotton (Wang *et al*., 2021b). Ectopic expression of *GhGAI2* in Arabidopsis led to reduced growth (reduced plant height, flower size, and silique size) (Aleman *et al*., 2008). Additionally, three endogenous bioactive GAs (GA1, GA3, and GA7) were downregulated in OE1. These results suggested that the *sbi1* phenotype might be associated with the GA biosynthesis or signalling, prompting us to investigate the relationship between *GhSBI1* and *GhGAIs*. We first examined the physical interaction between GhSBI1 and GhGAIs in yeast two‐hybrid assays. When the full GhGAI or GhSBI1 proteins were used as baits, strong transcriptional autoactivation was detected for both proteins. Further deletion studies revealed that in yeast cells, the later part of the transcriptional regulatory region (TRR) of GhSBI1 interacted with the C terminus of GhGAIs (Figure 6a,b; Figure S8). The highly diverged TRRs of NAC proteins can either activate or repress gene transcription, and some of them possess the protein‐binding ability. The C terminus of GAI proteins contains the GRAS domain, which is required for the growth repressor function of DELLA (Itoh *et al*., 2002). We also used an in vitro pull‐down assay to test the interaction between GhSBI1 and GhGAI3, which were produced and purified from *Escherichia coli*. GhSBI1 pulled down GhGAI3 in this assay (Figure 6c), indicating that they physically interact in vitro. The bi‐molecular fluorescence complementation (BiFC) and co‐immunoprecipitation (Co‐IP) assays also indicated that GhSBI1 interacts with GhGAI3 in plant cells (Figure 6d,e). Taken together, these results indicate that GhSBI1 directly interacts with GhGAIs in cotton.

### Identification of the genome‐wide direct targets of *GhSBI1*

**Figure 7:**
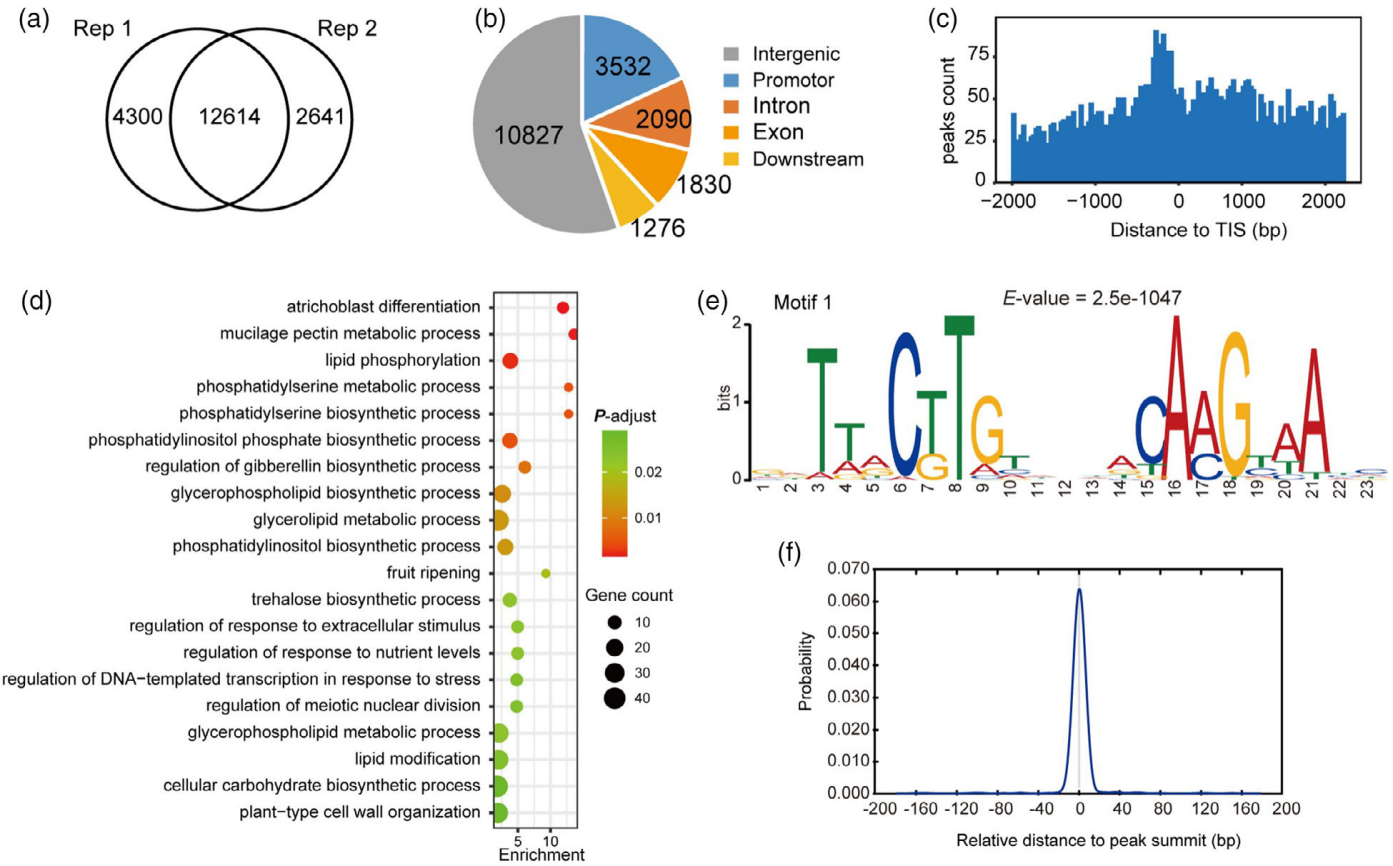
Genome‐wide identification of *GhSBI1* binding sites through DNA affinity purification sequencing (DAP‐seq). (a) The Venn diagram of peaks between two technical replicates in DAP‐seq. (b) Genome‐wide distribution of *GhSBI1* binding sites. Promoter regions were determined as the binding peaks within 3000‐bp upstream of translation initiation site (start codon). Downstream regions were determined as the binding peaks within 1000‐bp downstream of stop codon. (c) Distance from the centre of the binding site to translation initiation site (TIS) for all *GhSBI1* target genes. (d) Top 20 enriched Gene Ontology (GO) terms in the biological process class of *GhSBI1* bound genes. (e) The most enriched motif within the GhSBI1 binding peaks. The E‐value was calculated by MEME. (f) The position of Motif 1 relative to the DAP‐seq peak centres.

In order to gain a better understanding of *GhSBI1*‐mediated transcriptional regulation in cotton, we employed DNA affinity purification sequencing (DAP‐seq) to identify GhSBI1‐binding sites on a genome‐wide scale. A total of 19 555 binding peaks were identified, of which 12 614 peaks were detected in two technical repeats (Figure 7a). Overall, more than half (10827) of the binding peaks were distributed in the intergenic regions, and approximately 18% of the peaks (3532) were located in promoter regions (Figure 7b,c; Table S5). GO enrichment analysis of these promoter‐targeted genes (3466) revealed a significant overrepresentation of categories in the biological process class such as ‘mucilage pectin metabolic process’, ‘regulation of gibberellin biosynthetic process’, ‘regulation of meiotic nuclear division’, and ‘plant−type cell wall organization’, which are relevant to cell division or cell wall organization (Figure 7d).

Based on the binding peaks identified, a de novo motif prediction revealed the most enriched motif, Motif 1, which contains a palindromic 23‐bp conserved sequence (5′‐GDTTRCTTGTNNNACAAGYAAHC‐3′) (Figure 7e). This is reasonable considering that NAC proteins normally form a functional dimer using subdomain A located in the NAC domain (Puranik *et al*., 2012). Distribution analysis showed that Motif 1 was predominantly positioned at the summit of GhSBI1‐binding peaks (Figure 7f). Three other less enriched motifs with larger *E*‐values were also identified, but they are all positioned near the summit of the binding peaks (Figure S9). Therefore, Motif 1 is the most reliable GhSBI1‐binding motif in cotton.

### *GhSBI1* directly regulates core genes involved in internode elongation

**Figure 8:**
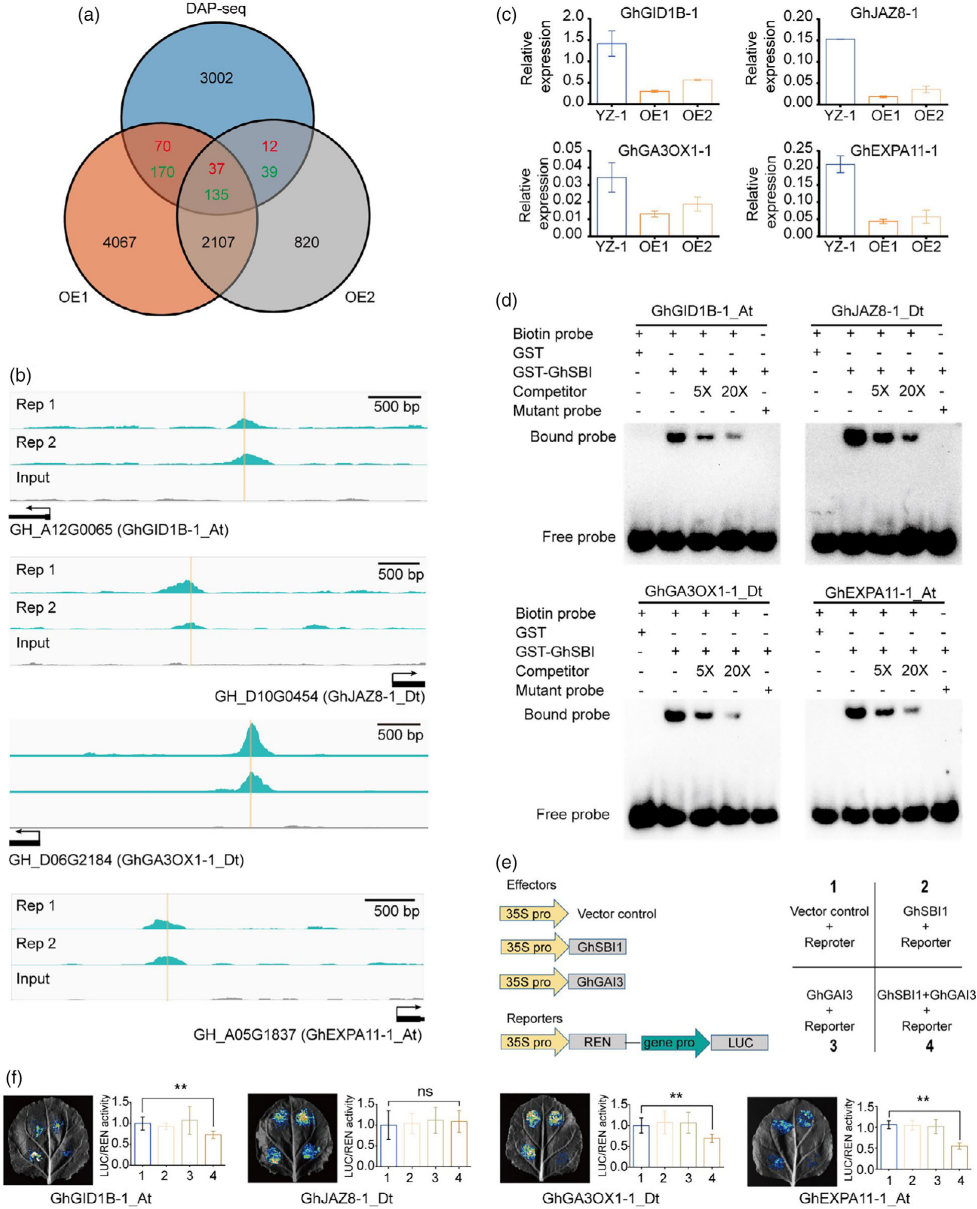
*GhSBI1*‐mediated direct regulation of genes responsible for internode elongation in cotton. (a) Venn diagram showing the overlap between *GhSBI1*‐bound genes as revealed by DAP‐seq and *GhSBI1*‐regulated genes identified by RNA‐seq. Red numbers indicate upregulated genes; green numbers indicate downregulated genes. (b) *GhSBI1* binding peaks (Reps 1 and 2) and negative control (Input) over the GhGID1B‐1_At, GhJAZ8‐1_Dt, GhGA3OX1‐1_Dt, and GhEXPA11‐1_At loci as determined by DAP‐seq. Orange lines indicate the positions of Motif 1 in binding peaks. (c) Verification of gene expression by RT‐qPCR. Error bars represent standard deviations of three biological replicates. (d) Electrophoretic mobility shift assay (EMSA) of the interactions between GhSBI1 and promoter fragments; biotin probe, biotin‐labelled probe with intact binding motif; competitor, unlabelled DNA probe with intact binding motif; mutant probe, biotin‐labelled probe with mutated binding motif. (e) Schematic representation of various constructs and their combinations used in the luciferase activation assay. (f) Quantification of relevant Luc activities. Data are presented as means ± standard deviation (*n* ≥ 5); *P*‐values are determined using Student's *t*‐test (** *P* < 0.01, ns *P* > 0.05).

To further identify the direct transcriptional targets of *GhSBI1*, we compared DEGs (OE1 vs. YZ‐1 and OE2 vs. YZ‐1) in RNA‐seq with potential *GhSBI1* target genes (3466 promoter‐targeted genes) in DAP‐seq. A total of 464 genes were found to be shared between the three datasets, of which 172 genes (37 upregulated and 135 downregulated) were common between OE1 and OE2 (Figure 8a; Table S6). Some genes related to hormone metabolism were found among these genes, such as *GIBBERELLIN 3‐OXIDASE 1* (*GA3OX1*, for GA biosynthesis) (downregulated), *GRETCHEN HAGEN3.6* (*GH3.6*, an IAA‐amido synthase) (downregulated), and *DIOXYGENASE FOR AUXIN OXIDATION 1* (*DAO1*, an IAA oxidase) (downregulated). Some genes in hormone signalling pathways were also found, such as *GID1B*, *ERF*, *JAZ*, and *PP2C*. Moreover, many transcription factors were found, including NAC (*NAC SECONDARY WALL THICKENING PROMOTING FACTOR 1*, *NAC5*, *KIRA1*, *CALMODULIN‐BINDING NAC PROTEIN*), MYB (*LOF2*, *PHYTOCLOCK 1*, *CAPRICE*, *MYB116*, *MYB121*, *MYB62*, *MYB102*), and homeobox (*WOX1*, *WOX3*). Notably, genes involved in stress/disease tolerance, such as *BAK1‐INTERACTING RECEPTOR‐LIKE KINASE 1* (*BIR1*), *COLD SHOCK DOMAIN PROTEIN 3* (*CSP3*), *WRKY40*, and *WRKY75*, were also found.

Four genes related to hormone biosynthesis/signalling or cell expansion were selected for further analysis, annotated as *GID1B* (*GhGID1B‐1_At*), *JAZ8* (*GhJAZ8‐1_Dt*), *GA3OX1* (*GhGA3OX1‐1_Dt*), and *EXPANSIN 11* (*GhEXPA11‐1_At*) (Figure 8b). *GA3OX1* encodes a GA 3‐oxidase that catalyses the terminal biosynthetic step of bioactive gibberellins (GA20 to GA1, GA5 to GA3, and GA9 to GA4) (Yamaguchi, 2008). The loss‐of‐function of *GA3OX1* results in the dwarf phenotype (Hu *et al*., 2008; Mitchum *et al*., 2006). *AtEXPA11* encodes a cell wall‐loosening enzyme and has been found to be involved in cell expansion (Gómez‐Ocampo *et al*., 2023; Hou *et al*., 2022; Simon *et al*., 2018). We first used qRT‐PCR to confirm the RNA‐seq results and found that these four genes were all downregulated in OE plants (Figure 8c). We then performed an electrophoretic mobility shift assay (EMSA), and the results confirmed the in vitro binding of GhSBI1 to corresponding synthetic oligonucleotide probes containing the core motif in the promoter regions of these four selected genes (Figure 8d). In addition, we conducted a transactivation assay using tobacco leaves to determine whether *GhSBI1* effectively represses the expression of the firefly luciferase (LUC) reporter gene driven by the promoters of these four genes. *GhSBI1* alone could not affect the promoter activity of these genes, but with the help of *GhGAI3* it could act as an inhibitor, except for *GhJAZ8‐1_Dt* (Figure 8e, f). These results suggest that *GhSBI1* represses the expression of these gibberellin biosynthesis/signalling or cell expansion genes by binding directly to their promoters, resulting in reduced internode elongation of shoots in cotton.

## Discussion

Branch length is an important yield‐related trait and shows wide variation among cotton accessions. However, until now few defined genes related to this trait and regulatory mechanisms have been characterized. Here, we identified a major‐effect QTL related to FB internode length in cotton, and after fine mapping *GhSBI1* was identified as the causal gene for this locus. Phylogenetic analysis revealed that *GhSBI1* is most homologous to the *CUC2* gene in *Arabidopsis*. *CUC2* has been reported to be involved in regulating shoot apical meristem initiation during post‐embryonic development (Aida *et al*., 1997; Hibara *et al*., 2006), but its roles in regulating internode elongation have been somewhat unclear. Our present study showed that increased expression of *GhSBI1* in cotton shortened the internode of FB and petiole. Moreover, knockout of *GhSBI1* also resulted in reduced internode length. Thus, achieving an appropriate expression level of *GhSBI1* is essential for proper internode elongation in cotton. In the present study, the *sbi1* mutant resulting from the mutation in the ghr‐miR164 targeting site happens to be a good example of the proper expression level of *GhSBI1*, whereas the expression driven by the 35S promoter was a bad example. Another good example is *Ideal Plant Architecture 1* (*IPA1*) in rice (Jiao *et al*., 2010; Miura *et al*., 2010). A point mutation in the miR156 targeting site disrupts the cleavage of the *OsSPL14* transcripts by OsmiR156, producing an ‘ideal’ plant type with increased grain yield. Therefore, in cotton and other crops, to achieve suitable plant architecture, manipulating the miR164 targeting sites of *CUC2s* might be a preferred method for fine‐tuning the expression of *CUC2s*.

**Figure 9:**
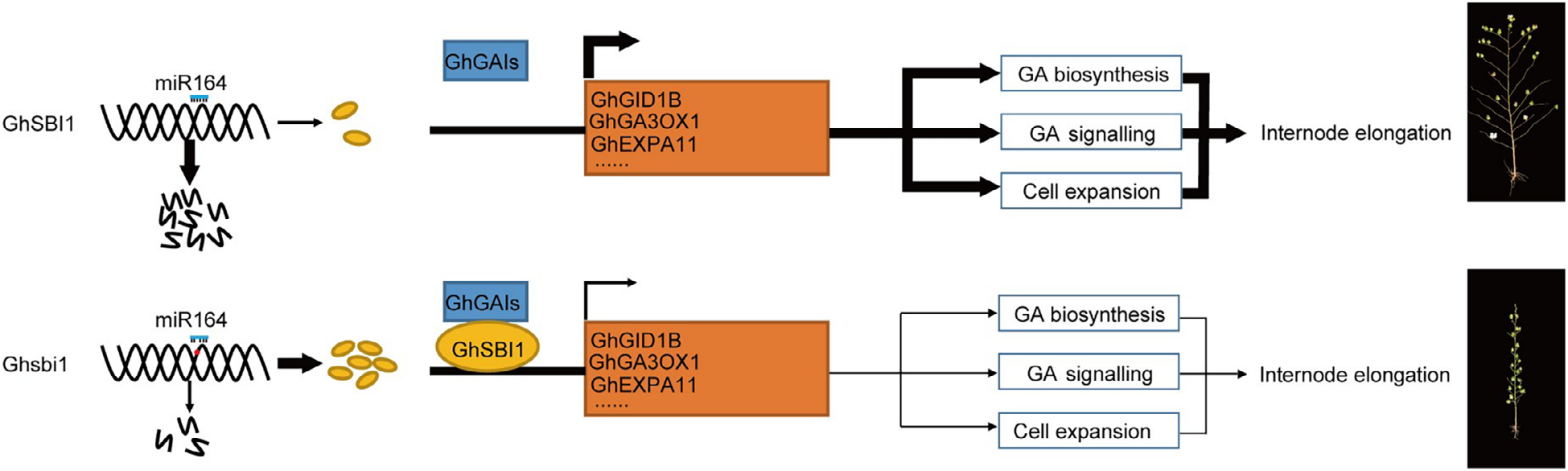
The proposed model showing the mechanism by which *GhSBI1* determines internode length.

Our present study showed that GhSBI1 can physically act with GhGAIs. In *Arabidopsis* and other plant species, GAIs, members of DELLA genes controlling cell expansion and cell division during vegetative growth and floral induction, are key repressors of GA signalling. DELLAs establish protein–protein interaction with diverse classes of transcription factors (TF) or transcriptional regulators (TR), and GA signalling accordingly controls the expression of a great deal of target genes functioning in varied pathways (Daviere're and Achard, 2016; Locascio et al., 2013). Our present results showed that GhSBI1 functioned as a newly identified DELLA interactor, and the expression of GID1B, a positive regulator of GA signalling, was also downregulated. In addition, the content of bioactive gibberellins also decreased in the *GhSBI1* overexpression plants, and the expression of *GA3OX1* was inhibited by the interaction of GhSBI1 and GhGAI3. These results indicate that *GhSBI1* affects FB internode elongation through GA biosynthesis and signalling pathway (Figure 9).

In *GhSBI1* overexpression plants, the levels of JA, ABA, and ETH were significantly increased. These three hormones play crucial roles in plant adaptation to adverse abiotic or biotic conditions. They also have important roles in regulating multiple plant developmental processes. ABA plays an important role in plant growth and development, including seed dormancy, fruit development, fruit ripening, and bud dormancy, usually in cross‐talk with ET and other hormones (Brookbank *et al*., 2021). Activation of the JA defence pathway has been shown to restrict plant growth in model plants (Kazan and Manners, 2011, 2012; Wan and Xin, 2022). In the context of internode elongation, high JA levels have been found to inhibit elongation of mesocotyl (rice), hypocotyl (Arabidopsis), and stems (*Nicotiana attenuate*) (Feng *et al*., 2017; Heinrich *et al*., 2013; Minami *et al*., 2018; Yang *et al*., 2012; Zhu *et al*., 2021). This inhibitory effect on stem elongation usually functions through GA pathway (Heinrich *et al*., 2013; Yang *et al*., 2012). High levels of JA antagonize the biosynthesis of gibberellins by inhibiting the expression of *GA20ox* and *GA13ox*, the key genes in GA production. JA delays GA‐mediated DELLA protein degradation, and knockdown of *OsCOI1*, a principal component of a receptor of JA, displays phenotypes similar to those of GA overproduction. JAZ repressors directly interfere with the interaction of DELLA and growth‐promoting phytochrome interacting factors (PIFs). On the other hand, JA signalling is enhanced by DELLA‐JAZs interaction (Yang *et al*., 2012). The accumulation of JA and the higher expression level of *GhGAIs* in *GhSBI1* overexpression plants reflect this crosstalk between JA and GA.

The expression of multiple stress‐related genes (such as *ZAT10*, *WRKY33*, and *COR27*) was found to be altered in the *GhSBI1* over‐expression lines, which meant plant defence might also be changed in the *sbi1* mutants. Actually, several mutants of plant architecture have been proven to have enhanced plant defence against biotic or abiotic stresses. *AiSheng98* (*AS98*), a dwarf cotton mutant resulting from elevated expression of a dehydration‐responsive element‐binding (DREB) transcription factor (*GhDREB1B*), has enhanced tolerance to drought and cold stress (Ji *et al*., 2021; Wang *et al*., 2021c). The *IPA1* rice mutant showed enhanced tolerance to rice blast disease, drought stress, cold stress, and salt stress (Chen *et al*., 2023; Jia *et al*., 2022a, 2022b). In the future, detailed characterization of the changes in stress/disease resistance associated with the *sbi1* mutant is needed to verify this speculation.

In summary, our results show that *GhSBI1*, a pleiotropic gene, is an important regulator of cotton plant architecture. In the breeding of elite cotton varieties, introgression or genetic engineering of *GhSBI1* could be used to achieve proper plant architecture for much higher yield potential. Considering the potential negative effect of *GhSBI1* on plant fertility, precise editing of *GhSBI1* using base editors in the promoter, DNA binding or miR164 target region could be an effective strategy to create different types of cotton plant architecture without compromising fertility. A recent study on *GhTFL1*, another important regulator of cotton plant architecture, showed that this strategy is effective (Wang *et al*., 2024). In addition, the potential alteration of stress/disease resistance associated with *GhSBI1* is also an important consideration in cotton breeding.

## Materials and methods

### Plant materials, growth conditions, and phenotypic analysis

CN, HJDGZ, and BYM are upland cotton inbred lines characterized by exceptionally short fruiting branches (FB). TM‐1 is a standard genetic line of upland cotton with a sequenced reference genome. To conduct inheritance analysis and map‐based cloning, CN was crossed with TM‐1 to generate F_2_, F_2:3_, and BC_1_ populations. For phenotypic analysis and DNA extraction, the parent lines and populations were planted in the experimental field of the Institute of Cotton Research, Chinese Academic Agricultural Sciences (ICR, CAAS) located in (Anyang, 35°12′N, 113°37′ E). For phenotypic evaluation, the mean internode length was obtained by measuring the first and second internodes of the fifth to eighth fruiting branches of each plant.

### Hormone treatments

Seven‐week‐old seedlings were treated with gibberellic acid (GA3) (50 or 75 μM), 3‐indole‐acetic acid (IAA) (100 or 200 μM), and brassinolide (BL) (5 or 10 μM), with distilled water as a control. The solution was sprayed over the entire leaf surface of the plant every 7 days, and this treatment was conducted for six times in succession.

### QTL‐seq and fine mapping

Two DNA pools (long‐internode pool and short‐internode pool) were constructed by mixing equal amounts of DNA from 50‐long‐FB‐internode (>7.5 cm) and 50‐short‐FB‐internode (<2.5 cm) F_2_ plants from the 2016 experiment. Total genomic DNA of CN, TM‐1, and two pools were subjected to whole‐genome sequencing on an Illumina HiSeq2500 platform using the PE150 strategy. After quality filtering, the clean reads were aligned to the TM‐1 reference genome (ZJU, V2.1) (http://cotton.zju.edu.cn/) using the Burrows–Wheeler–Alignment (BWA) tool. Calling of single‐nucleotide polymorphisms (SNPs) and insertions and deletions (indels) was performed using the GATK toolkit. Only polymorphic homozygous SNPs/indels between CN and TM‐1 with base quality scores greater than 20 were retained for further analysis. The QTL‐seq method based on SNP index association analysis was performed (Takagi *et al*., 2013). The minimum read coverages of SNPs/inDels were set to 5 and 10 for parent and pool, respectively. Only the genomic region with a ∆ (SNP‐index) above the 99% confidence interval threshold was considered as a candidate QTL.

For the fine mapping of the *sbi1* mutation, the eight SNP markers from the mapping interval on chromosome D01 were used to genotype the F_2_ plants to identify recombinants. The identified recombinants were self‐pollinated to generate F_2:3_ families, and F_2:3_ plants were further phenotyped in the next growing season. The phenotypic distribution of the F_2:3_ family was used to verify the genotype of the *GhSBI1* locus for each recombinant.

### Gene constructs and cotton transformation

For the CaMV35S:GhSBI1 overexpression construct, a 1080‐bp full‐length *GhSBI1* cDNA was PCR‐amplified from CN and sub‐cloned into the binary vector pBI121. For the pGhSBI1:GhSBI1 construct, a 5152‐bp genomic fragment spanning from 3093‐bp upstream of the translation start codon to 51‐bp downstream of the termination codon of *GH_D01G0566* was PCR‐amplified from CN, and sub‐cloned into the binary vector pBI121. For the pGhSBI:GUS reporter construct, the CaMV35S promoter in pBI121 was replaced with the *GhSBI1* promoter (−1147 to −1 bp to the initiation ATG). For the *GhSBI1* knockout construct, an sgRNA targeting *GhSBI1* was cloned into the pRGEB32‐GhU6.9 vector (Wang *et al*., 2018). These constructs were transformed into Agrobacterium strain LBA4404 for cotton transformation (Jin et al., 2006). YZ‐1 was the transgenic receptor. Primers used in this study are listed in Table S7.

### RNA extraction and qRT‐PCR analysis

RNA was extracted from the collected samples using the RNAprep Pure Plant Kit (Tiangen, Beijing, China). First‐strand cDNA was reverse transcribed using a PrimeScript® RT reagent kit with a gDNA Eraser (Takara, Dalian, China). qRT‐PCR reactions were conducted on a 7500 Fast Real‐Time PCR System (Applied Biosystems, StepOnePlus, USA) using SYBR® Premix Ex Taq™ (Tli RNaseH Plus) (Takara, Dalian, China). Cotton ACTIN14 (GenBank accession number: AY305733) was used as the reference gene to normalize the expression levels of the target genes. Primers are listed in Table S7.

### Scanning electron microscopy (SEM) imaging

For SEM, the middle section of the mature internode of FB was collected and fixed with 2.5% glutaraldehyde. The detailed SEM procedures were performed as reported (Hu *et al*., 2018). Cell length was estimated using Image J software (https://imagej.nih.gov/ij/).

### RLM‐ race

To determine the ghr‐miR164 cleavage site in *GhSBI1*, the RLM‐RACE was performed using the SMARTer RACE 5′/3′ Kit (Takara, Dalian, China), according to the manufacturer's instructions. Total RNA extracted from shoot apices of FB of TM‐1 was used. The final PCR products were inserted into pEASY T1‐cloning (TransGen Biotech, Beijing) for sequencing. Primers used in this assay are listed in Table S7.

### Degradome sequencing

Total RNA was extracted from the shoot apices of FB of TM‐1, and the degradome library was constructed according to the manufacturer's instructions. Degradome sequencing was performed on an Illumina HiSeq2500 platform using the SE50 strategy at Lianchuan Bio, Hangzhou, China. CleaveLand v3.0.1 was used to predict the miRNA cleavage sites.

### RNA‐seq and data analysis

The shoot apices of FB from YZ‐1 and the two transgenic lines were collected at the bud stage for RNA extraction and sequencing. Sequencing libraries were constructed according to the user manual and sequenced to generate 150‐bp paired‐end reads on a BGISEQ‐500. Routine data analysis was performed as previously described (Hu *et al*., 2018; Tian *et al*., 2022).

### Measurements of endogenous phytohormones and flavonoids

The first internodes of the fifth to eighth FB at the early elongation stage were collected at the bud stage. Sample preparation and content determination were performed by MetWare (http://www.metware.cn/) using the AB Sciex QTRAP 6500 LC‐MS/MS platform according to previously described methods (Chen *et al*., 2020; Luo *et al*., 2022). Eight classes of phytohormones (auxin, CK, ABA, JA, SA, GA, ETH, and SL) and classes of flavonoids (anthocyanins, biflavonoids, chalcones, flavanols, flavanones, flavanonols, flavone glycosides, flavones, flavonols, isoflavanones, phenolic acids, xanthones, and other flavonoids). Three biological replicates were performed for each sample.

### Yeast two‐hybrid assays

The GAL4‐based two‐hybrid system was used for Y2H experiments. The pGBKT7 and pGADT7 vectors were used for bait and prey construction, respectively. Bait and prey constructs were transformed into the yeast strain Y2HGold. Transformed yeast cells were grown on an SD/−Trp/−Leu (SD/TL) double dropout medium for selection, and protein–protein interactions were tested on an SD/−Trp/−Leu/‐His/−Ade quadruple dropout medium with (QD/X) or without X‐α‐Gal (QD).

### GST pull‐down assay

The full‐length coding sequence of *GhSBI1* was cloned into the pGEX‐6P‐1 vector as bait (GST‐GhSBI1). The full‐length coding sequence of *GhGAI3* was cloned into the pET32a vector as prey (His‐GhGAI3). The plasmids were transformed into *E. coli* strain Rosetta (DE3). The recombinant proteins were induced with 0.2 mM IPTG. Purified bait and prey proteins were mixed and incubated at 4 °C overnight and then purified using glutathione‐conjugated agarose beads (Solarbio, Beijing, China). The eluted proteins were then subjected to immunoblot analysis using anti‐GST or anti‐His antibodies (Proteintech, Rosemont, IL).

### BiFC assay

The coding sequences of *GhSBI1* and *GhGAI3* were cloned into the binary yellow fluorescent protein (YFP) BiFC vectors pXY106 (YFP^n^) and pXY104 (YFP^c^), respectively, resulting in the recombinant plasmids GhSBI‐YFP^n^ and GhGAI3‐YFP^c^. The plasmids were transferred into *Agrobacterium tumefaciens* strain GV3101 and were transiently expressed in tobacco leaves by agroinfiltration. 48 h after transformation, the YFP signals in the transfected cells were examined using an Olympus FV1000 confocal microscope (Olympus Kyoto, Japan).

### Co‐IP assay

The full‐length coding sequence of *GhSBI1* was fused downstream of green fluorescent protein (GFP) in the vector pMDC43 to generate GFP‐GhSBI1 overexpressing constructs. The full‐length coding sequence of *GhGAI3* was fused into the vector pAN580 to generate the GhGAI3‐Flag overexpressing construct. Co‐immunoprecipitated proteins were prepared from rice protoplasts transiently expressing GFP‐GhSBI1 and GhGAI3‐Flag using GFP‐Trap beads and then analysed by immunoblotting with anti‐FLAG (working dilution 1:5000) or anti‐GFP (1:5000) antibodies.

### DAP‐seq

DAP‐seq experiments were performed according to the method described in the previous study (Bartlett *et al*., 2017). Briefly, genomic DNA was extracted from shoot apices of TM‐1 plants, and a genomic DNA library was prepared using the MICH TLX DNA‐seq kit (PerkinElmer, Inc., Austin, TX, USA) according to the manufacturer's instructions. The GhSBI1 protein was prepared using the TNT SP6 Coupled Wheat Germ Extract System (Promega, Madison, WI, USA) and subsequently purified using HaloTag Beads (Promega, USA). The GhSBI1‐HaloTag bead mixtures or HaloTag beads (input negative control) were incubated with the ‘TM‐1’ genomic DNA libraries. The eluted GhSBI1‐binding DNA fragments were subjected to high‐throughput sequencing analysis on an Illumina NavoSeq6000 instrument platform (San Diego, CA, USA). DAP‐seq reads were aligned to the TM‐1 reference genome (ZJU, V2.1) using Bowtie2. DAP‐seq peaks were identified by using MACS2 (fold enrichment >2 and *q*‐value <0.05). MEME‐CHIP software (version 5.0.5) was used to identify conservative motifs in the peaks.

### EMSA assay

The coding region of *GhSBI1* was cloned into the pGEX‐6P‐1 vector. The recombinant proteins were induced with 0.2 mm isopropyl‐beta‐D‐thiogalactopyranoside (IPTG). The promoter fragment of *GhGID1B‐1_At*, *GhJAZ8‐1_Dt*, *GhGA3OX1‐1_Dt*, and *GhEXPA11‐1_At* containing the intact or mutated GhSBI1‐binding site was labelled with biotin on both ends of the probe. Unlabelled probes were used as cold competitors. The LightShift Chemiluminescent EMSA kit (Thermo Fisher Scientific, Shanghai, China) was used to perform EMSA according to the manufacturer's protocol. The probes are listed in Table S7.

### Dual‐luciferase reporter assay

For validation of the DNA–protein interactions between GhSBI1 and its target genes, promoters of target genes were cloned individually into the pGreenII‐0800‐LUC vector as reporters. The coding sequences of *GhSBI1* and *GhGAI3* were cloned into pGreenII‐62‐SK as effectors. The Agrobacterium strains transformed with the reporter and effector constructs were co‐infiltrated into tobacco leaves. The LUC (firefly luciferase) and REN (Renilla luciferase) activities were measured using a dual‐luciferase reporter assay kit (E1910, Promega, Madison, WI).

To verify the splicing effect of ghr‐miR164 on its target site, the pGreenII Dual‐Luciferase miRNA Target Expression Vector derived from pGreenII‐0800‐LUC was used to quantitatively assess the cleavage activity of ghr‐miR164 (Liu *et al*., 2014; Xue *et al*., 2019). The ghr‐miR164 target site with flanking sequence (TM‐1 or CN type, 36 bp) was inserted into the 3′ untranslated regions of the LUC gene as a reporter. The precursor of ghr‐miR164 (ghr‐MIR164, 101 bp) was cloned into pGreenII‐62‐SK as an effectors. The *Agrobacterium* strains transformed with the reporter and effector constructs were co‐infiltrated into tobacco protoplast, and the ratio of LUC/REN was measured. The sequences of the ghr‐miR164 target site and ghr‐MIR164 are listed in Table S7.

## Conflicts of interest

The authors declared that they have no conflict of interest.

## Author contributions

W.C. and Y.Z. conceived and instructed the study; W.Z., L.W., X.L., J.W., J.P., and Y.L. performed the experiments and data analysis; S.Z., S.F., and J.Y. participated in the experiments and bred the plant materials; W.Z., L.W., and W.C. wrote the manuscript. All authors read and approved the manuscript.

## Supporting information


**Data S1** Supporting Information.

**Figure S1** Exogenous GA3 treatments of CN and TM‐1.

**Figure S2** Fine mapping of *GhSBI1*.

**Figure S3** Alignment of genomic DNA sequences of *GH_D01G0566*.

**Figure S4** Promoter activity assay of *GH_D01G0566*.

**Figure S5** Phenotype of transgenic plants with high levels of *GhSBI1* driven by the cauliflower mosaic virus (CaMV) 35S promoter.

**Figure S6** Characterization of cotton CRISPR editing lines on *GhSBI1* genes.

**Figure S7** Phenotype of *GhSBI1* knockout lines.

**Figure S8** GhSBI1 physically interacts with GhGAI1, GhGAI2, and GhGAI4 in yeast two‐hybrid assays.

**Figure S9** The enriched motifs and their positions within the GhSBI1‐binding peaks.

**Table S1** Summary of variations in the final mapping interval.
**Table S2** Content of endogenous phytohormones.
**Table S3** Content of endogenous flavonoids.
**Table S4** Summary of common DEG between OE1 vs. YZ‐1 and OE2 vs. YZ‐1.
**Table S5** Summary of GhSBI1 binding peaks.
**Table S6** Summary of transcriptional targets of GhSBI1 in DAP‐seq and RNA‐seq.
**Table S7** Primers used in this study.

## Data Availability

The RNA‐seq data were deposited in the NCBI SRA database (BioProject accession number: PRJNA755799).
