# Supplementary material for: GhSBI1, a CUP‐SHAPED COTYLEDON 2 homologue, modulates branch internode elongation in cotton
Source: Plant Biotechnol J. 2024 Jul 26;22(11):3175–93. doi: 10.1111/pbi.14439 (PMC11500989; doi:10.1111/pbi.14439)
Supplement: Supplementary file 1 — Data S1 Supporting Information. [file PBI-22-3175-s011.docx]

**Supporting materials legends**

**Figure S1** Exogenous GA3 treatments of CN and TM-1. (a) Summary of internode length of fruiting branch in GA3 treatments (n > 10). The 1st and 2nd internodes were measured to get the average value for each branch. (b) Phenotype of CN in GA3 treatments. Scale bar = 10 cm.

**Figure S2** Fine mapping of *GhSBI1*. (a) Δ (SNP-index) on D01. Grey dot indicates SNP or Indel. Green dot indicates average SNP-index in every 1-Mb interval. (b) Logarithm of the odds (LOD) profiles of internode length quantitative trait loci (QTL). (c) Fine mapping of *GhSBI1* using recombinant plants. Gray segments indicate heterozygous regions. The red vertical lines indicate the average values of F_2:3_ families. Dots indicate the values of F_2:3_ plants. The phenotypic distribution and mean phenotype of the F_2:3_ families were used to determine the inferred genotypes of *GhSBI1*. (d) Schematic diagram of the final mapped interval for *GhSBI1*. Green rectangles indicate exons. (e) Expression value (Fragments per kilo base of transcript per million mapped fragments, FPKM) of candidate genes in public RNA-seq data. Hai7124 is a Sea Island (*G. barbadense*) line.

**Figure S4** Promotor activity assay of *GH_D01G0566*. (a) Schematic diagram of constructs and GUS activity. (b) qRT-PCR analyses of mRNA levels of the GUS gene. Error bars represent standard deviations of three biological replicates.

**Figure S5** Phenotype of transgenic plants with high levels of *GhSBI1* driven by the cauliflower mosaic virus (CaMV) 35S promoter. (a) Phenotype of transgenic plants. NT1, non-transgenic line 1. (b) qRT-PCR analyses of mRNA levels of the *GhSBI1* gene in leaves. (c) Phenotype of fruiting branches. FB, fruiting branch. VB, vegetative branch. Numbers indicate the internodes of branches. Scale bar = 10 cm.

**Figure S6** Characterization of cotton CRISPR editing lines on *GhSBI1* genes. (a) Schematic diagram of target mutation of *GhSBI1* genes in editing lines. (b) Summary of frequencies of cotyledon fusion in editing lines.

**Figure S7** Phenotype of *GhSBI1* knockout lines. (a) Phenotype of fused cotyledons. (b) Cup-shaped-cotyledon plant without shoot apical meristem. (c) Fused-cotyledon plants with shoots. (d) Fused shoots of knockout lines. Red arrows indicate fused stems. (e) Whole flower. (f) Defoliated flower. (g) Style. (h) Stigma. (i) Anther. (j) Filament. (k) Statistical analysis of style length. (l) Statistical analysis of filament number. (m) – (o) Abnormal flowers of knockout lines. (p) Pollen stainability. Significant differences are indicated by ** (*P* < 0.01) (Student’s t-test). Scale bars = 10 mm in (e)–(g) and 1 mm in (h)-(j) and (p).

**Figure S8** GhSBI1 physically interacts with GhGAI1, GhGAI2 and GhGAI4 in yeast two-hybrid assays. (a) Autoactivation detection of GhSBI1. (b) Schematic diagram of different truncated versions of GhGAIs. (c) Autoactivation detection of GhGAIs. (d) Yeast two-hybrid assays of the interactions between GhSBI1 and GhGAIs.

**Figure S9** The enriched motifs and their positions within the GhSBI1 binding peaks.

**Table S1** Summary of variations in the final mapping interval.

**Table S2** Content of endogenous phytohormones.

**Table S3** Content of endogenous flavonoids.

**Table S4** Summary of common DEG between OE1 vs. YZ-1 and OE2 vs. YZ-1.

**Table S5** Summary of GhSBI1 binding peaks.

**Table S6** Summary of transcriptional targets of GhSBI1 in DAP-seq and RNA-seq.

**Table S7** Primers used in this study.
