# Supplementary material for: GhSBI1, a CUP‐SHAPED COTYLEDON 2 homologue, modulates branch internode elongation in cotton
Source: Plant Biotechnol J. 2024 Jul 26;22(11):3175–93. doi: 10.1111/pbi.14439 (PMC11500989; doi:10.1111/pbi.14439)
Supplement: Supplementary file 4 — Figure S3 Alignment of genomic DNA sequences of GH_D01G0566. [file PBI-22-3175-s005.docx]

**Figure S3** Alignment of genomic DNA sequences of GH_D01G0566. TM-1, YZ-1, Jin668, ZMS35, ZM24, San4080 and NDM8 are normal-fruiting-branch lines (internode length: 8-10 cm). CN, HJDGZ and BYM are *sbi1* mutants (internode length: ~2 cm). Jin7 has an intermediate type of fruiting branch (internode length: ~5cm).

Type-1

Type-2

**HJDGZ AGTTGAAAGAGAAGGGAGAAAGCTCTAGTTCGATGTATTTCATAATAGTGGAGAGATTACTGGAATGCAATTGTAAATGA BYM AGTTGAAAGAGAAGGGAGAAAGCTCTAGTTCGATGTATTTCATAATAGTGGAGAGATTACTGGAATGCAATTGTAAATGA CN AGTTGAAAGAGAAGGGAGAAAGCTCTAGTTCGATGTATTTCATAATAGTGGAGAGATTACTGGAATGCAATTGTAAATGA NDM8 AGTTGAAAGAGAAGGGAGAAAGCTCTAGTTCGATGTATTTCATAATAGTGGAGAGATTACTGGAATGCAATTGTAAATGA San4080 AGTTGAAAGAGAAGGGAGAAAGCTCTAGTTCGATGTATTTCATAATAGTGGAGAGATTACTGGAATGCAATTGTAAATGA ZM24 AGTTGAAAGAGAAGGGAGAAAGCTCTAGTTCGATGTATTTCATAATAGTGGAGAGATTACTGGAATGCAATTGTAAATGA ZMS35 AGTTGAAAGAGAAGGGAGAAAGCTCTAGTTCGATGTATTTCATAATAGTGGAGAGATTACTGGAATGCAATTGTAAATGA Jin7 AGTTGAAAGAGAAGGGAGAAAGCTCTAGTTCGATGTATTTCATAATAGTGGAGAGATTACTGGAATGCAATTGTAAATGA Jin668 AGTTGAAAGAGAAGGGAGAAAGCTCTAGTTCGATGTATTTCATAATAGTGGAGAGATTACTGGAATGCAATTGTAAATGA YZ-1 AGTTGAAAGAGAAGGGAGAAAGCTCTAGTTCGATGTATTTCATAATAGTGGAGAGATTACTGGAATGCAATTGTAAATGA TM-1 AGTTGAAAGAGAAGGGAGAAAGCTCTAGTTCGATGTATTTCATAATAGTGGAGAGATTACTGGAATGCAATTGTAAATGA**

**HJDGZ TGTCTCCAAAACCCTAATAGTTTTGAATAGTTTAGTGATAAAGTTATAATTTTTTTTTAAATTAAGTGATCAAACTATAA BYM TGTCTCCAAAACCCTAATAGTTTTGAATAGTTTAGTGATAAAGTTATAATTTTTTTTTAAATTAAGTGATCAAACTATAA CN TGTCTCCAAAACCCTAATAGTTTTGAATAGTTTAGTGATAAAGTTATAATTTTTTTTTAAATTAAGTGATCAAACTATAA NDM8 TGTCTCCAAAACCCTAATAGTTTTGAATAGTTTAGTGATAAAGTTATAATTTTTTTTTAAATTAAGTGATCAAACTATAA San4080 TGTCTCCAAAACCCTAATAGTTTTGAATAGTTTAGTGATAAAGTTATAATTTTTTTTTAAATTAAGTGATCAAACTATAA ZM24 TGTCTCCAAAACCCTAATAGTTTTGAATAGTTTAGTGATAAAGTTATAATTTTTTTTTAAATTAAGTGATCAAACTATAA ZMS35 TGTCTCCAAAACCCTAATAGTTTTGAATAGTTTAGTGATAAAGTTATAATTTTTTTTTAAATTAAGTGATCAAACTATAA Jin7 TGTCTCCAAAACCCTAATAGTTTTGAATAGTTTAGTGATAAAGTTATAATTTTTTTTTAAATTAAGTGATCAAACTATAA Jin668 TGTCTCCAAAACCCTAATAGTTTTGAATAGTTTAGTGATAAAGTTATAATTTTTTTTTAAATTAAGTGATCAAACTATAA YZ-1 TGTCTCCAAAACCCTAATAGTTTTGAATAGTTTAGTGATAAAGTTATAATTTTTTTTTAAATTAAGTGATCAAACTATAA TM-1 TGTCTCCAAAACCCTAATAGTTTTGAATAGTTTAGTGATAAAGTTATAATTTTTTTTTAAATTAAGTGATCAAACTATAA**

**HJDGZ ACTTATTAATAGTTAAAAGATTTTAAATGTAATTGAGGAGTTAATTAATAGTCATAAAACAATTGTTAGCTATTTTCAAA BYM ACTTATTAATAGTTAAAAGATTTTAAATGTAATTGAGGAGTTAATTAATAGTCATAAAACAATTGTTAGCTATTTTCAAA CN ACTTATTAATAGTTAAAAGATTTTAAATGTAATTGAGGAGTTAATTAATAGTCATAAAACAATTGTTAGCTATTTTCAAA NDM8 ACTTATTAATAGTTAAAAGATTTTAAATGTAATTGAGGAGTTAATTAATAGTCATAAAACAATTGTTAGCTATTTTCAAA San4080 ACTTATTAATAGTTAAAAGATTTTAAATGTAATTGAGGAGTTAATTAATAGTCATAAAACAATTGTTAGCTATTTTCAAA ZM24 ACTTATTAATAGTTAAAAGATTTTAAATGTAATTGAGGAGTTAATTAATAGTCATAAAACAATTGTTAGCTATTTTCAAA ZMS35 ACTTATTAATAGTTAAAAGATTTGAAATGTAATTGAGGAGTTAATTAATAGTCATAAAACAATTGTTAGCTATTTTCAAA Jin7 ACTTATTAATAGTTAAAAGATTTGAAATGTAATTGAGGAGTTAATTAATAGTCATAAAACAATTGTTAGCTATTTTCAAA Jin668 ACTTATTAATAGTTAAAAGATTTGAAATGTAATTGAGGAGTTAATTAATAGTCATAAAACAATTGTTAGCTATTTTCAAA YZ-1 ACTTATTAATAGTTAAAAGATTTGAAATGTAATTGAGGAGTTAATTAATAGTCATAAAACAATTGTTAGCTATTTTCAAA TM-1 ACTTATTAATAGTTAAAAGATTTGAAATGTAATTGAGGAGTTAATTAATAGTCATAAAACAATTGTTAGCTATTTTCAAA**

**HJDGZ TATAATTTAATGTTACTTTTTGAAATTAAATAAATAAGGTATACATTTAGGACATTGGGTGTAATTTTCCCATCAATTAT BYM TATAATTTAATGTTACTTTTTGAAATTAAATAAATAAGGTATACATTTAGGACATTGGGTGTAATTTTCCCATCAATTAT CN TATAATTTAATGTTACTTTTTGAAATTAAATAAATAAGGTATACATTTAGGACATTGGGTGTAATTTTCCCATCAATTAT NDM8 TATAATTTAATGTTACTTTTTGAAATTAAATAAATAAGGTATACATTTAGGACATTGGGTGTAATTTTCCCATCAATTAT San4080 TATAATTTAATGTTACTTTTTGAAATTAAATAAATAAGGTATACATTTAGGACATTGGGTGTAATTTTCCCATCAATTAT ZM24 TATAATTTAATGTTACTTTTTGAAATTAAATAAATAAGGTATACATTTAGGACATTGGGTGTAATTTTCCCATCAATTAT ZMS35 TATAATTTAATGTTACTTTTTGAAATTAAATAAATAAGGTATACATTTAGGACATTGGATGTAATTTTCCCATCAATTAT Jin7 TATAATTTAATGTTACTTTTTGAAATTAAATAAATAAGGTATACATTTAGGACATTGGATGTAATTTTCCCATCAATTAT Jin668 TATAATTTAATGTTACTTTTTGAAATTAAATAAATAAGGTATACATTTAGGACATTGGATGTAATTTTCCCATCAATTAT YZ-1 TATAATTTAATGTTACTTTTTGAAATTAAATAAATAAGGTATACATTTAGGACATTGGATGTAATTTTCCCATCAATTAT TM-1 TATAATTTAATGTTACTTTTTGAAATTAAATAAATAAGGTATACATTTAGGACATTGGATGTAATTTTCCCATCAATTAT**

**HJDGZ CTAACTTCGTAGTTAGTAAAATGAAGAAAGGGTTTTAGTTTTTATTAGTAGAAGAAAAAGAGGAAGGGAAGGGTGGCGTG BYM CTAACTTCGTAGTTAGTAAAATGAAGAAAGGGTTTTAGTTTTTATTAGTAGAAGAAAAAGAGGAAGGGAAGGGTGGCGTG CN CTAACTTCGTAGTTAGTAAAATGAAGAAAGGGTTTTAGTTTTTATTAGTAGAAGAAAAAGAGGAAGGGAAGGGTGGCGTG NDM8 CTAACTTCGTAGTTAGTAAAATGAAGAAAGGGTTTTAGTTTTTATTAGTAGAAGAAAAAGAGGAAGGGAAGGGTGGCGTG San4080 CTAACTTCGTAGTTAGTAAAATGAAGAAAGGGTTTTAGTTTTTATTAGTAGAAGAAAAAGAGGAAGGGAAGGGTGGCGTG ZM24 CTAACTTCGTAGTTAGTAAAATGAAGAAAGGGTTTTAGTTTTTATTAGTAGAAGAAAAAGAGGAAGGGAAGGGTGGCGTG ZMS35 CTAACTTCGTAGTTAGTAAAATGAAGAAAGGATTTTAGTTTTTATTAGTAGAAGAAAAAGAGGAAGGGAAGGGTGGCGTG Jin7 CTAACTTCGTAGTTAGTAAAATGAAGAAAGGATTTTAGTTTTTATTAGTAGAAGAAAAAGAGGAAGGGAAGGGTGGCGTG Jin668 CTAACTTCGTAGTTAGTAAAATGAAGAAAGGATTTTAGTTTTTATTAGTAGAAGAAAAAGAGGAAGGGAAGGGTGGCGTG YZ-1 CTAACTTCGTAGTTAGTAAAATGAAGAAAGGATTTTAGTTTTTATTAGTAGAAGAAAAAGAGGAAGGGAAGGGTGGCGTG TM-1 CTAACTTCGTAGTTAGTAAAATGAAGAAAGGATTTTAGTTTTTATTAGTAGAAGAAAAAGAGGAAGGGAAGGGTGGCGTG**

**HJDGZ TAATGACAATGTGTGTATAATTTTTAATTTTTGTACTGTTCTGACAAGAAGAGATATGAAACAGCTTTATCGATTCATTA BYM TAATGACAATGTGTGTATAATTTTTAATTTTTGTACTGTTCTGACAAGAAGAGATATGAAACAGCTTTATCGATTCATTA CN TAATGACAATGTGTGTATAATTTTTAATTTTTGTACTGTTCTGACAAGAAGAGATATGAAACAGCTTTATCGATTCATTA NDM8 TAATGACAATGTGTGTATAATTTTTAATTTTTGTACTGTTCTGACAAGAAGAGATATGAAACAGCTTTATCGATTCATTA San4080 TAATGACAATGTGTGTATAATTTTTAATTTTTGTACTGTTCTGACAAGAAGAGATATGAAACAGCTTTATCGATTCATTA ZM24 TAATGACAATGTGTGTATAATTTTTAATTTTTGTACTGTTCTGACAAGAAGAGATATGAAACAGCTTTATCGATTCATTA ZMS35 TAATGACAATGTGTGTATAATTTTTAATTTTTGTACTGTTCTGACAAGAAGAGATATGAAACAGCTTTATCGATTCATTA Jin7 TAATGACAATGTGTGTATAATTTTTAATTTTTGTACTGTTCTGACAAGAAGAGATATGAAACAGCTTTATCGATTCATTA**

**Jin668 TAATGACAATGTGTGTATAATTTTTAATTTTTGTACTGTTCTGACAAGAAGAGATATGAAACAGCTTTATCGATTCATTA YZ-1 TAATGACAATGTGTGTATAATTTTTAATTTTTGTACTGTTCTGACAAGAAGAGATATGAAACAGCTTTATCGATTCATTA TM-1 TAATGACAATGTGTGTATAATTTTTAATTTTTGTACTGTTCTGACAAGAAGAGATATGAAACAGCTTTATCGATTCATTA**

**HJDGZ CATTTTTACATTATATAAATAATAT-----------TACTTTCTCAAAATCAATCTATCACCCTTTTCTTTTCTTGTCTC BYM CATTTTTACATTATATAAATAATAT-----------TACTTTCTCAAAATCAATCTATCACCCTTTTCTTTTCTTGTCTC CN CATTTTTACATTATATAAATAATAT-----------TACTTTCTCAAAATCAATCTATCACCCTTTTCTTTTCTTGTCTC NDM8 CATTTTTACATTATATAAATAATAT-----------TACTTTCTCAAAATCAATCTATCACCCTTTTCTTTTCTTGTCTC San4080 CATTTTTACATTATATAAATAATAT-----------TACTTTCTCAAAATCAATCTATCACCCTTTTCTTTTCTTGTCTC ZM24 CATTTTTACATTATATAAATAATAT-----------TACTTTCTCAAAATCAATCTATCACCCTTTTCTTTTCTTGTCTC ZMS35 CATTTTTACATTATATAAATAATATATAAATAATATTACTTTCTCAAAATCAATCTATCACCCTTTTCTTTTCTTGTCTC Jin7 CATTTTTACATTATATAAATAATATATAAATAATATTACTTTCTCAAAATCAATCTATCACCCTTTTCTTTTCTTGTCTC Jin668 CATTTTTACATTATATAAATAATATATAAATAATATTACTTTCTCAAAATCAATCTATCACCCTTTTCTTTTCTTGTCTC YZ-1 CATTTTTACATTATATAAATAATATATAAATAATATTACTTTCTCAAAATCAATCTATCACCCTTTTCTTTTCTTGTCTC TM-1 CATTTTTACATTATATAAATAATATATAAATAATATTACTTTCTCAAAATCAATCTATCACCCTTTTCTTTTCTTGTCTC**

**HJDGZ TTAAAAAGAAAAAAGAAAAAGAAAAAGAAATTTTACTTTCTCCTTTCATTTTTTCTTACACTGACAGGACAGGGAATACC BYM TTAAAAAGAAAAAAGAAAAAGAAAAAGAAATTTTACTTTCTCCTTTCATTTTTTCTTACACTGACAGGACAGGGAATACC CN TTAAAAAGAAAAAAGAAAAAGAAAAAGAAATTTTACTTTCTCCTTTCATTTTTTCTTACACTGACAGGACAGGGAATACC NDM8 TTAAAAAGAAAAAAGAAAAAGAAAAAGAAATTTTACTTTCTCCTTTCATTTTTTCTTACACTGACAGGACAGGGAATACC San4080 TTAAAAAGAAAAAAGAAAAAGAAAAAGAAATTTTACTTTCTCCTTTCATTTTTTCTTACACTGACAGGACAGGGAATACC ZM24 TTAAAAAGAAAAAAGAAAAAGAAAAAGAAATTTTACTTTCTCCTTTCATTTTTTCTTACACTGACAGGACAGGGAATACC ZMS35 TCAAAAAAAAAAAAGAAAA-GAAAAAGAAATTTTACTTTCTCCTTTCATTTTTTCTTACACTGACAGGACAGGGAATACC Jin7 TCAAAAAAAAAAAAGAAAA-GAAAAAGAAATTTTACTTTCTCCTTTCATTTTTTCTTACACTGACAGGACAGGGAATACC Jin668 TCAAAAAAAAAAAAGAAAA-GAAAAAGAAATTTTACTTTCTCCTTTCATTTTTTCTTACACTGACAGGACAGGGAATACC YZ-1 TCAAAAAAAAAAAAGAAAA-GAAAAAGAAATTTTACTTTCTCCTTTCATTTTTTCTTACACTGACAGGACAGGGAATACC TM-1 TCAAAAAAAAAAAAGAAAA-GAAAAAGAAATTTTACTTTCTCCTTTCATTTTTTCTTACACTGACAGGACAGGGAATACC**

**HJDGZ TGCACACTTTCTCACGCACGCACGCATACTCTAATCCCCCCATACATCACCCCCTCTCTCAATCCCTCTCATCATAATCC BYM TGCACACTTTCTCACGCACGCACGCATACTCTAATCCCCCCATACATCACCCCCTCTCTCAATCCCTCTCATCATAATCC CN TGCACACTTTCTCACGCACGCACGCATACTCTAATCCCCCCATACATCACCCCCTCTCTCAATCCCTCTCATCATAATCC NDM8 TGCACACTTTCTCACGCACGCACGCATACTCTAATCCCCCCATACATCACCCCCTCTCTCAATCCCTCTCATCATAATCC San4080 TGCACACTTTCTCACGCACGCACGCATACTCTAATCCCCCCATACATCACCCCCTCTCTCAATCCCTCTCATCATAATCC ZM24 TGCACACTTTCTCACGCACGCACGCATACTCTAATCCCCCCATACATCACCCCCTCTCTCAATCCCTCTCATCATAATCC ZMS35 TGCACACTTTCTCACGCACGCACGCATACTCTAATCCCCCCATACATCACCCCCTCTCTCAATCCCTCTCATCATAATCC Jin7 TGCACACTTTCTCACGCACGCACGCATACTCTAATCCCCCCATACATCACCCCCTCTCTCAATCCCTCTCATCATAATCC Jin668 TGCACACTTTCTCACGCACGCACGCATACTCTAATCCCCCCATACATCACCCCCTCTCTCAATCCCTCTCATCATAATCC YZ-1 TGCACACTTTCTCACGCACGCACGCATACTCTAATCCCCCCATACATCACCCCCTCTCTCAATCCCTCTCATCATAATCC TM-1 TGCACACTTTCTCACGCACGCACGCATACTCTAATCCCCCCATACATCACCCCCTCTCTCAATCCCTCTCATCATAATCC**

**HJDGZ ATACCCCCTCTCCCTATCTCTCTCCTTAGAAAGAAAAGAGAAAGATTTCGCAACAGGACACTGCAGAGAAACAACACAGA BYM ATACCCCCTCTCCCTATCTCTCTCCTTAGAAAGAAAAGAGAAAGATTTCGCAACAGGACACTGCAGAGAAACAACACAGA CN ATACCCCCTCTCCCTATCTCTCTCCTTAGAAAGAAAAGAGAAAGATTTCGCAACAGGACACTGCAGAGAAACAACACAGA NDM8 ATACCCCCTCTCCCTATCTCTCTCCTTAGAAAGAAAAGAGAAAGATTTCGCAACAGGACACTGCAGAGAAACAACACAGA San4080 ATACCCCCTCTCCCTATCTCTCTCCTTAGAAAGAAAAGAGAAAGATTTCGCAACAGGACACTGCAGAGAAACAACACAGA ZM24 ATACCCCCTCTCCCTATCTCTCTCCTTAGAAAGAAAAGAGAAAGATTTCGCAACAGGACACTGCAGAGAAACAACACAGA ZMS35 ATACCCCCTCTCCCTATCTCTCTCCTTAGAAAGAAAAGAGAAAGATTTCGCAACAGGACACTGCAGAGAAACAACACAGA Jin7 ATACCCCCTCTCCCTATCTCTCTCCTTAGAAAGAAAAGAGAAAGATTTCGCAACAGGACACTGCAGAGAAACAACACAGA Jin668 ATACCCCCTCTCCCTATCTCTCTCCTTAGAAAGAAAAGAGAAAGATTTCGCAACAGGACACTGCAGAGAAACAACACAGA YZ-1 ATACCCCCTCTCCCTATCTCTCTCCTTAGAAAGAAAAGAGAAAGATTTCGCAACAGGACACTGCAGAGAAACAACACAGA TM-1 ATACCCCCTCTCCCTATCTCTCTCCTTAGAAAGAAAAGAGAAAGATTTCGCAACAGGACACTGCAGAGAAACAACACAGA**

**HJDGZ TTTTCTCAGCTTCAAATTATCCCCCACCCCCTAATCTTCTAATATTATTAATATTTTCTGTTGCCTCTCTTTCCTCTGTC BYM TTTTCTCAGCTTCAAATTATCCCCCACCCCCTAATCTTCTAATATTATTAATATTTTCTGTTGCCTCTCTTTCCTCTGTC CN TTTTCTCAGCTTCAAATTATCCCCCACCCCCTAATCTTCTAATATTATTAATATTTTCTGTTGCCTCTCTTTCCTCTGTC NDM8 TTTTCTCAGCTTCAAATTATCCCCCACCCCCTAATCTTCTAATATTATTAATATTTTCTGTTGCCTCTCTTTCCTCTGTC San4080 TTTTCTCAGCTTCAAATTATCCCCCACCCCCTAATCTTCTAATATTATTAATATTTTCTGTTGCCTCTCTTTCCTCTGTC ZM24 TTTTCTCAGCTTCAAATTATCCCCCACCCCCTAATCTTCTAATATTATTAATATTTTCTGTTGCCTCTCTTTCCTCTGTC ZMS35 TTTTCTCAGCTTCAAATTATCCCCCACCCCCTAATCTTCTAATATTATTAATATTTTCTGTTGCCTCTCTTTCCTCTGTC Jin7 TTTTCTCAGCTTCAAATTATCCCCCACCCCCTAATCTTCTAATATTATTAATATTTTCTGTTGCCTCTCTTTCCTCTGTC Jin668 TTTTCTCAGCTTCAAATTATCCCCCACCCCCTAATCTTCTAATATTATTAATATTTTCTGTTGCCTCTCTTTCCTCTGTC YZ-1 TTTTCTCAGCTTCAAATTATCCCCCACCCCCTAATCTTCTAATATTATTAATATTTTCTGTTGCCTCTCTTTCCTCTGTC TM-1 TTTTCTCAGCTTCAAATTATCCCCCACCCCCTAATCTTCTAATATTATTAATATTTTCTGTTGCCTCTCTTTCCTCTGTC**

**HJDGZ ACTCGATTCATCAAATCCCTTTTTTCCCCCTCACCCCCCATTGTCACACCCCATATAACAACCCCTCCTCCCTTCCTTTT BYM ACTCGATTCATCAAATCCCTTTTTTCCCCCTCACCCCCCATTGTCACACCCCATATAACAACCCCTCCTCCCTTCCTTTT CN ACTCGATTCATCAAATCCCTTTTTTCCCCCTCACCCCCCATTGTCACACCCCATATAACAACCCCTCCTCCCTTCCTTTT NDM8 ACTCGATTCATCAAATCCCTTTTTTCCCCCTCACCCCCCATTGTCACACCCCATATAACAACCCCTCCTCCCTTCCTTTT San4080 ACTCGATTCATCAAATCCCTTTTTTCCCCCTCACCCCCCATTGTCACACCCCATATAACAACCCCTCCTCCCTTCCTTTT ZM24 ACTCGATTCATCAAATCCCTTTTTTCCCCCTCACCCCCCATTGTCACACCCCATATAACAACCCCTCCTCCCTTCCTTTT ZMS35 ACTCGATTCATCAAATCCCTTTTTTCCCCCTCACCCCCCATTGTGACACCCCATATAACAACCCCTCCTTCCTTCCTTTT Jin7 ACTCGATTCATCAAATCCCTTTTTTCCCCCTCACCCCCCATTGTGACACCCCATATAACAACCCCTCCTTCCTTCCTTTT Jin668 ACTCGATTCATCAAATCCCTTTTTTCCCCCTCACCCCCCATTGTGACACCCCATATAACAACCCCTCCTTCCTTCCTTTT**

**YZ-1 ACTCGATTCATCAAATCCCTTTTTTCCCCCTCACCCCCCATTGTGACACCCCATATAACAACCCCTCCTTCCTTCCTTTT TM-1 ACTCGATTCATCAAATCCCTTTTTTCCCCCTCACCCCCCATTGTGACACCCCATATAACAACCCCTCCTTCCTTCCTTTT**

**HJDGZ CTTCCCATTCCTCAGCAGCTTCAAAACCCTTTGTTCCCTTTGTTCATTTCATGATGTTTTAGCTTTGGTGTTGAAGTTTA BYM CTTCCCATTCCTCAGCAGCTTCAAAACCCTTTGTTCCCTTTGTTCATTTCATGATGTTTTAGCTTTGGTGTTGAAGTTTA CN CTTCCCATTCCTCAGCAGCTTCAAAACCCTTTGTTCCCTTTGTTCATTTCATGATGTTTTAGCTTTGGTGTTGAAGTTTA NDM8 CTTCCCATTCCTCAGCAGCTTCAAAACCCTTTGTTCCCTTTGTTCATTTCATGATGTTTTAGCTTTGGTGTTGAAGTTTA San4080 CTTCCCATTCCTCAGCAGCTTCAAAACCCTTTGTTCCCTTTGTTCATTTCATGATGTTTTAGCTTTGGTGTTGAAGTTTA ZM24 CTTCCCATTCCTCAGCAGCTTCAAAACCCTTTGTTCCCTTTGTTCATTTCATGATGTTTTAGCTTTGGTGTTGAAGTTTA ZMS35 CTTCCCATTCCTCAGCAGCTTCAAAACCCTTTGTTCCCTTTGTTCATTTCATGATGTTTTAGCTTTGGTGTTGAAGTTTA Jin7 CTTCCCATTCCTCAGCAGCTTCAAAACCCTTTGTTCCCTTTGTTCATTTCATGATGTTTTAGCTTTGGTGTTGAAGTTTA Jin668 CTTCCCATTCCTCAGCAGCTTCAAAACCCTTTGTTCCCTTTGTTCATTTCATGATGTTTTAGCTTTGGTGTTGAAGTTTA YZ-1 CTTCCCATTCCTCAGCAGCTTCAAAACCCTTTGTTCCCTTTGTTCATTTCATGATGTTTTAGCTTTGGTGTTGAAGTTTA TM-1 CTTCCCATTCCTCAGCAGCTTCAAAACCCTTTGTTCCCTTTGTTCATTTCATGATGTTTTAGCTTTGGTGTTGAAGTTTA**

**HJDGZ GGGTTCTTAGTTGTTTTTGGTTCCAGGTTTTTAAGGGTTTTGAAAATGGACAGTTACCATCATTTTGACAATGGTGATAC BYM GGGTTCTTAGTTGTTTTTGGTTCCAGGTTTTTAAGGGTTTTGAAAATGGACAGTTACCATCATTTTGACAATGGTGATAC CN GGGTTCTTAGTTGTTTTTGGTTCCAGGTTTTTAAGGGTTTTGAAAATGGACAGTTACCATCATTTTGACAATGGTGATAC NDM8 GGGTTCTTAGTTGTTTTTGGTTCCAGGTTTTTAAGGGTTTTGAAAATGGACAGTTACCATCATTTTGACAATGGTGATAC San4080 GGGTTCTTAGTTGTTTTTGGTTCCAGGTTTTTAAGGGTTTTGAAAATGGACAGTTACCATCATTTTGACAATGGTGATAC ZM24 GGGTTCTTAGTTGTTTTTGGTTCCAGGTTTTTAAGGGTTTTGAAAATGGACAGTTACCATCATTTTGACAATGGTGATAC ZMS35 GGGTTCTTAGTTGTTTTTGGTTCCAGGTTTTTAAGGGTTTTGAAAATGGACAGTTACCATCATTTTGACAATGGTGATAC Jin7 GGGTTCTTAGTTGTTTTTGGTTCCAGGTTTTTAAGGGTTTTGAAAATGGACAGTTACCATCATTTTGACAATGGTGATAC Jin668 GGGTTCTTAGTTGTTTTTGGTTCCAGGTTTTTAAGGGTTTTGAAAATGGACAGTTACCATCATTTTGACAATGGTGATAC YZ-1 GGGTTCTTAGTTGTTTTTGGTTCCAGGTTTTTAAGGGTTTTGAAAATGGACAGTTACCATCATTTTGACAATGGTGATAC TM-1 GGGTTCTTAGTTGTTTTTGGTTCCAGGTTTTTAAGGGTTTTGAAAATGGACAGTTACCATCATTTTGACAATGGTGATAC**

**Start codon**

**HJDGZ ACATTTGCCTCCTGGTTTTCGTTTCCATCCTACTGATGAAGAGCTTATTACTTACTACCTGTTGAAGAAAGTTCTTGATA BYM ACATTTGCCTCCTGGTTTTCGTTTCCATCCTACTGATGAAGAGCTTATTACTTACTACCTGTTGAAGAAAGTTCTTGATA CN ACATTTGCCTCCTGGTTTTCGTTTCCATCCTACTGATGAAGAGCTTATTACTTACTACCTGTTGAAGAAAGTTCTTGATA NDM8 ACATTTGCCTCCTGGTTTTCGTTTCCATCCTACTGATGAAGAGCTTATTACTTACTACCTGTTGAAGAAAGTTCTTGATA San4080 ACATTTGCCTCCTGGTTTTCGTTTCCATCCTACTGATGAAGAGCTTATTACTTACTACCTGTTGAAGAAAGTTCTTGATA ZM24 ACATTTGCCTCCTGGTTTTCGTTTCCATCCTACTGATGAAGAGCTTATTACTTACTACCTGTTGAAGAAAGTTCTTGATA ZMS35 ACATTTGCCTCCTGGTTTTCGTTTCCATCCTACTGATGAAGAGCTTATTACTTACTACCTGTTGAAGAAAGTTCTTGATA Jin7 ACATTTGCCTCCTGGTTTTCGTTTCCATCCTACTGATGAAGAGCTTATTACTTACTACCTGTTGAAGAAAGTTCTTGATA Jin668 ACATTTGCCTCCTGGTTTTCGTTTCCATCCTACTGATGAAGAGCTTATTACTTACTACCTGTTGAAGAAAGTTCTTGATA YZ-1 ACATTTGCCTCCTGGTTTTCGTTTCCATCCTACTGATGAAGAGCTTATTACTTACTACCTGTTGAAGAAAGTTCTTGATA TM-1 ACATTTGCCTCCTGGTTTTCGTTTCCATCCTACTGATGAAGAGCTTATTACTTACTACCTGTTGAAGAAAGTTCTTGATA**

**HJDGZ GTAGCTTTACTGGTAGAGCTATAGCTGAAGTTGACCTTAACAAGTGTGAGCCTTGGGAACTTCCTGGTATGTAATCATTA BYM GTAGCTTTACTGGTAGAGCTATAGCTGAAGTTGACCTTAACAAGTGTGAGCCTTGGGAACTTCCTGGTATGTAATCATTA CN GTAGCTTTACTGGTAGAGCTATAGCTGAAGTTGACCTTAACAAGTGTGAGCCTTGGGAACTTCCTGGTATGTAATCATTA NDM8 GTAGCTTTACTGGTAGAGCTATAGCTGAAGTTGACCTTAACAAGTGTGAGCCTTGGGAACTTCCTGGTATGTAATCATTA San4080 GTAGCTTTACTGGTAGAGCTATAGCTGAAGTTGACCTTAACAAGTGTGAGCCTTGGGAACTTCCTGGTATGTAATCATTA ZM24 GTAGCTTTACTGGTAGAGCTATAGCTGAAGTTGACCTTAACAAGTGTGAGCCTTGGGAACTTCCTGGTATGTAATCATTA ZMS35 GTAGCTTTACTGGTAGAGCTATAGCTGAAGTTGACCTTAACAAGTGTGAGCCTTGGGAACTTCCTGGTATGTAATCATTA Jin7 GTAGCTTTACTGGTAGAGCTATAGCTGAAGTTGACCTTAACAAGTGTGAGCCTTGGGAACTTCCTGGTATGTAATCATTA Jin668 GTAGCTTTACTGGTAGAGCTATAGCTGAAGTTGACCTTAACAAGTGTGAGCCTTGGGAACTTCCTGGTATGTAATCATTA YZ-1 GTAGCTTTACTGGTAGAGCTATAGCTGAAGTTGACCTTAACAAGTGTGAGCCTTGGGAACTTCCTGGTATGTAATCATTA TM-1 GTAGCTTTACTGGTAGAGCTATAGCTGAAGTTGACCTTAACAAGTGTGAGCCTTGGGAACTTCCTGGTATGTAATCATTA End of exon1**

**HJDGZ ACACTTCTCTGGACTACCTTTTCATTTTTTACACAAAATTCGTTAATGTAATCATACTGTTTATAACTCTCTTTTCTTTC BYM ACACTTCTCTGGACTACCTTTTCATTTTTTACACAAAATTCGTTAATGTAATCATACTGTTTATAACTCTCTTTTCTTTC CN ACACTTCTCTGGACTACCTTTTCATTTTTTACACAAAATTCGTTAATGTAATCATACTGTTTATAACTCTCTTTTCTTTC NDM8 ACACTTCTCTGGACTACCTTTTCATTTTTTACACAAAATTCGTTAATGTAATCATACTGTTTATAACTCTCTTTTCTTTC San4080 ACACTTCTCTGGACTACCTTTTCATTTTTTACACAAAATTCGTTAATGTAATCATACTGTTTATAACTCTCTTTTCTTTC ZM24 ACACTTCTCTGGACTACCTTTTCATTTTTTACACAAAATTCGTTAATGTAATCATACTGTTTATAACTCTCTTTTCTTTC ZMS35 ACACTTCTCTGGACTACCTTTTCATTTTTTACACAAAATTCGTTAATGTAATCATACTGTTTATAACTCTCTTTTCTTTC Jin7 ACACTTCTCTGGACTACCTTTTCATTTTTTACACAAAATTCGTTAATGTAATCATACTGTTTATAACTCTCTTTTCTTTC Jin668 ACACTTCTCTGGACTACCTTTTCATTTTTTACACAAAATTCGTTAATGTAATCATACTGTTTATAACTCTCTTTTCTTTC YZ-1 ACACTTCTCTGGACTACCTTTTCATTTTTTACACAAAATTCGTTAATGTAATCATACTGTTTATAACTCTCTTTTCTTTC TM-1 ACACTTCTCTGGACTACCTTTTCATTTTTTACACAAAATTCGTTAATGTAATCATACTGTTTATAACTCTCTTTTCTTTC**

**HJDGZ TTTTATGCATTGGACATGGAATATGGCAGAGAAAGCAAAGATGGGAGAGAAAGAGTGGTATTTTTTTAGTTTAAGAGATA BYM TTTTATGCATTGGACATGGAATATGGCAGAGAAAGCAAAGATGGGAGAGAAAGAGTGGTATTTTTTTAGTTTAAGAGATA CN TTTTATGCATTGGACATGGAATATGGCAGAGAAAGCAAAGATGGGAGAGAAAGAGTGGTATTTTTTTAGTTTAAGAGATA NDM8 TTTTATGCATTGGACATGGAATATGGCAGAGAAAGCAAAGATGGGAGAGAAAGAGTGGTATTTTTTTAGTTTAAGAGATA San4080 TTTTATGCATTGGACATGGAATATGGCAGAGAAAGCAAAGATGGGAGAGAAAGAGTGGTATTTTTTTAGTTTAAGAGATA ZM24 TTTTATGCATTGGACATGGAATATGGCAGAGAAAGCAAAGATGGGAGAGAAAGAGTGGTATTTTTTTAGTTTAAGAGATA**

**ZMS35 TTTTATGCATTGGACATGGAATATGGCAGAGAAAGCAAAGATGGGAGAGAAAGAGTGGTATTTTTTTAGTTTAAGAGATA Jin7 TTTTATGCATTGGACATGGAATATGGCAGAGAAAGCAAAGATGGGAGAGAAAGAGTGGTATTTTTTTAGTTTAAGAGATA Jin668 TTTTATGCATTGGACATGGAATATGGCAGAGAAAGCAAAGATGGGAGAGAAAGAGTGGTATTTTTTTAGTTTAAGAGATA YZ-1 TTTTATGCATTGGACATGGAATATGGCAGAGAAAGCAAAGATGGGAGAGAAAGAGTGGTATTTTTTTAGTTTAAGAGATA TM-1 TTTTATGCATTGGACATGGAATATGGCAGAGAAAGCAAAGATGGGAGAGAAAGAGTGGTATTTTTTTAGTTTAAGAGATA**

**Start of exon2**

**HJDGZ GGAAATACCCAACTGGGTTGAGAACTAACCGAGCTACTGAAGCTGGTTATTGGAAAGCTACTGGGAAAGATAGGGAGATT BYM GGAAATACCCAACTGGGTTGAGAACTAACCGAGCTACTGAAGCTGGTTATTGGAAAGCTACTGGGAAAGATAGGGAGATT CN GGAAATACCCAACTGGGTTGAGAACTAACCGAGCTACTGAAGCTGGTTATTGGAAAGCTACTGGGAAAGATAGGGAGATT NDM8 GGAAATACCCAACTGGGTTGAGAACTAACCGAGCTACTGAAGCTGGTTATTGGAAAGCTACTGGGAAAGATAGGGAGATT San4080 GGAAATACCCAACTGGGTTGAGAACTAACCGAGCTACTGAAGCTGGTTATTGGAAAGCTACTGGGAAAGATAGGGAGATT ZM24 GGAAATACCCAACTGGGTTGAGAACTAACCGAGCTACTGAAGCTGGTTATTGGAAAGCTACTGGGAAAGATAGGGAGATT ZMS35 GGAAATACCCAACTGGGTTGAGAACTAACCGAGCTACTGAAGCTGGTTATTGGAAAGCTACTGGGAAAGATAGGGAGATT Jin7 GGAAATACCCAACTGGGTTGAGAACTAACCGAGCTACTGAAGCTGGTTATTGGAAAGCTACTGGGAAAGATAGGGAGATT Jin668 GGAAATACCCAACTGGGTTGAGAACTAACCGAGCTACTGAAGCTGGTTATTGGAAAGCTACTGGGAAAGATAGGGAGATT YZ-1 GGAAATACCCAACTGGGTTGAGAACTAACCGAGCTACTGAAGCTGGTTATTGGAAAGCTACTGGGAAAGATAGGGAGATT TM-1 GGAAATACCCAACTGGGTTGAGAACTAACCGAGCTACTGAAGCTGGTTATTGGAAAGCTACTGGGAAAGATAGGGAGATT**

**HJDGZ TACAGCTCAAAGACGAGTGCACTTGTTGGGATGAAGAAAACCCTTGTTTTTTATAGAGGTAGAGCTCCTAAAGGAGAAAA BYM TACAGCTCAAAGACGAGTGCACTTGTTGGGATGAAGAAAACCCTTGTTTTTTATAGAGGTAGAGCTCCTAAAGGAGAAAA CN TACAGCTCAAAGACGAGTGCACTTGTTGGGATGAAGAAAACCCTTGTTTTTTATAGAGGTAGAGCTCCTAAAGGAGAAAA NDM8 TACAGCTCAAAGACGAGTGCACTTGTTGGGATGAAGAAAACCCTTGTTTTTTATAGAGGTAGAGCTCCTAAAGGAGAAAA San4080 TACAGCTCAAAGACGAGTGCACTTGTTGGGATGAAGAAAACCCTTGTTTTTTATAGAGGTAGAGCTCCTAAAGGAGAAAA ZM24 TACAGCTCAAAGACGAGTGCACTTGTTGGGATGAAGAAAACCCTTGTTTTTTATAGAGGTAGAGCTCCTAAAGGAGAAAA ZMS35 TACAGCTCAAAGACGAGTGCACTTGTTGGGATGAAGAAAACCCTTGTTTTTTATAGAGGTAGAGCTCCTAAAGGAGAAAA Jin7 TACAGCTCAAAGACGAGTGCACTTGTTGGGATGAAGAAAACCCTTGTTTTTTATAGAGGTAGAGCTCCTAAAGGAGAAAA Jin668 TACAGCTCAAAGACGAGTGCACTTGTTGGGATGAAGAAAACCCTTGTTTTTTATAGAGGTAGAGCTCCTAAAGGAGAAAA YZ-1 TACAGCTCAAAGACGAGTGCACTTGTTGGGATGAAGAAAACCCTTGTTTTTTATAGAGGTAGAGCTCCTAAAGGAGAAAA TM-1 TACAGCTCAAAGACGAGTGCACTTGTTGGGATGAAGAAAACCCTTGTTTTTTATAGAGGTAGAGCTCCTAAAGGAGAAAA**

**HJDGZ AAGCAACTGGGTCATGCATGAATATCGCCTCGAAGGAAAATTTGCTTACCATTATCTCTCCAGAAGCTCAAAGGTCTCCC BYM AAGCAACTGGGTCATGCATGAATATCGCCTCGAAGGAAAATTTGCTTACCATTATCTCTCCAGAAGCTCAAAGGTCTCCC CN AAGCAACTGGGTCATGCATGAATATCGCCTCGAAGGAAAATTTGCTTACCATTATCTCTCCAGAAGCTCAAAGGTCTCCC NDM8 AAGCAACTGGGTCATGCATGAATATCGCCTCGAAGGAAAATTTGCTTACCATTATCTCTCCAGAAGCTCAAAGGTCTCCC San4080 AAGCAACTGGGTCATGCATGAATATCGCCTCGAAGGAAAATTTGCTTACCATTATCTCTCCAGAAGCTCAAAGGTCTCCC ZM24 AAGCAACTGGGTCATGCATGAATATCGCCTCGAAGGAAAATTTGCTTACCATTATCTCTCCAGAAGCTCAAAGGTCTCCC ZMS35 AAGCAACTGGGTCATGCATGAATATCGCCTCGAAGGAAAATTTGCTTACCATTATCTCTCCAGAAGCTCAAAGGTCTCCC Jin7 AAGCAACTGGGTCATGCATGAATATCGCCTCGAAGGAAAATTTGCTTACCATTATCTCTCCAGAAGCTCAAAGGTCTCCC Jin668 AAGCAACTGGGTCATGCATGAATATCGCCTCGAAGGAAAATTTGCTTACCATTATCTCTCCAGAAGCTCAAAGGTCTCCC YZ-1 AAGCAACTGGGTCATGCATGAATATCGCCTCGAAGGAAAATTTGCTTACCATTATCTCTCCAGAAGCTCAAAGGTCTCCC TM-1 AAGCAACTGGGTCATGCATGAATATCGCCTCGAAGGAAAATTTGCTTACCATTATCTCTCCAGAAGCTCAAAGGTCTCCC**

**End of exon2**

**HJDGZ CCCTCTCTCTCTCACTTAATTGCTTCTTTATGCTGTGTTTTACTTTCTTGTTTCATGGTCGATCTTTGGTTCTTCTGGTA BYM CCCTCTCTCTCTCACTTAATTGCTTCTTTATGCTGTGTTTTACTTTCTTGTTTCATGGTCGATCTTTGGTTCTTCTGGTA CN CCCTCTCTCTCTCACTTAATTGCTTCTTTATGCTGTGTTTTACTTTCTTGTTTCATGGTCGATCTTTGGTTCTTCTGGTA NDM8 CCCTCTCTCTCTCACTTAATTGCTTCTTTATGCTGTGTTTTACTTTCTTGTTTCATGGTCGATCTTTGGTTCTTCTGGTA San4080 CCCTCTCTCTCTCACTTAATTGCTTCTTTATGCTGTGTTTTACTTTCTTGTTTCATGGTCGATCTTTGGTTCTTCTGGTA ZM24 CCCTCTCTCTCTCACTTAATTGCTTCTTTATGCTGTGTTTTACTTTCTTGTTTCATGGTCGATCTTTGGTTCTTCTGGTA ZMS35 CCCTCTCTCTCTCACTTAATTGCTTCTTTATGCTGTGTTTTACTTTCTTGTTTCATGGTCGATCTTTGGTTCTTCTGGTA Jin7 CCCTCTCTCTCTCACTTAATTGCTTCTTTATGCTGTGTTTTACTTTCTTGTTTCATGGTCGATCTTTGGTTCTTCTGGTA Jin668 CCCTCTCTCTCTCACTTAATTGCTTCTTTATGCTGTGTTTTACTTTCTTGTTTCATGGTCGATCTTTGGTTCTTCTGGTA YZ-1 CCCTCTCTCTCTCACTTAATTGCTTCTTTATGCTGTGTTTTACTTTCTTGTTTCATGGTCGATCTTTGGTTCTTCTGGTA TM-1 CCCTCTCTCTCTCACTTAATTGCTTCTTTATGCTGTGTTTTACTTTCTTGTTTCATGGTCGATCTTTGGTTCTTCTGGTA**

**HJDGZ CTGTGTCCGTACTGTTTTTGCTTCATCTTTGCTTCCTGAGAGCTGAAATTTGCTCTCAAGCAATGGTGTATGCGTATAAC BYM CTGTGTCCGTACTGTTTTTGCTTCATCTTTGCTTCCTGAGAGCTGAAATTTGCTCTCAAGCAATGGTGTATGCGTATAAC CN CTGTGTCCGTACTGTTTTTGCTTCATCTTTGCTTCCTGAGAGCTGAAATTTGCTCTCAAGCAATGGTGTATGCGTATAAC NDM8 CTGTGTCCGTACTGTTTTTGCTTCATCTTTGCTTCCTGAGAGCTGAAATTTGCTCTCAAGCAATGGTGTATGCGTATAAC San4080 CTGTGTCCGTACTGTTTTTGCTTCATCTTTGCTTCCTGAGAGCTGAAATTTGCTCTCAAGCAATGGTGTATGCGTATAAC ZM24 CTGTGTCCGTACTGTTTTTGCTTCATCTTTGCTTCCTGAGAGCTGAAATTTGCTCTCAAGCAATGGTGTATGCGTATAAC ZMS35 CTGTGTCCGTACTGTTTTTGCTTCATCTTTGCTTCCTGAGAGCTGAAATTTGCTCTCAAGCAATGGTGTATGCGTATAAC Jin7 CTGTGTCCGTACTGTTTTTGCTTCATCTTTGCTTCCTGAGAGCTGAAATTTGCTCTCAAGCAATGGTGTATGCGTATAAC Jin668 CTGTGTCCGTACTGTTTTTGCTTCATCTTTGCTTCCTGAGAGCTGAAATTTGCTCTCAAGCAATGGTGTATGCGTATAAC YZ-1 CTGTGTCCGTACTGTTTTTGCTTCATCTTTGCTTCCTGAGAGCTGAAATTTGCTCTCAAGCAATGGTGTATGCGTATAAC TM-1 CTGTGTCCGTACTGTTTTTGCTTCATCTTTGCTTCCTGAGAGCTGAAATTTGCTCTCAAGCAATGGTGTATGCGTATAAC**

**HJDGZ ACAGTACTTTACTACATTTATTATGTTACCGTATGCTGTTGTTTCATTTAAGGAGCTGGAAAAGGTATGTTGGGAAATCT BYM** **ACAGTACTTTACTACATTTATTATGTTACCGTATGCTGTTGTTTCATTTAAGGAGCTGGAAAAGGTATGTTGGGAAATCT CN** **ACAGTACTTTACTACATTTATTATGTTACCGTATGCTGTTGTTTCATTTAAGGAGCTGGAAAAGGTATGTTGGGAAATCT NDM8 ACAGTACTTTACTACATTTATTATGTTACCGTATGCTGTTGTTTCATTTAAGGAGCTGGAAAAGGTATGTTGGGAAATCT**

**San4080 ACAGTACTTTACTACATTTATTATGTTACCGTATGCTGTTGTTTCATTTAAGGAGCTGGAAAAGGTATGTTGGGAAATCT ZM24 ACAGTACTTTACTACATTTATTATGTTACCGTATGCTGTTGTTTCATTTAAGGAGCTGGAAAAGGTATGTTGGGAAATCT ZMS35 ACAGTACTTTACTACATTTATTATGTTACCGTATGCTGTTGTTTCATTTAAGGAGCTGGAAAAGGTATGTTGGGAAATCT Jin7 ACAGTACTTTACTACATTTATTATGTTACCGTATGCTGTTGTTTCATTTAAGGAGCTGGAAAAGGTATGTTGGGAAATCT Jin668 ACAGTACTTTACTACATTTATTATGTTACCGTATGCTGTTGTTTCATTTAAGGAGCTGGAAAAGGTATGTTGGGAAATCT YZ-1 ACAGTACTTTACTACATTTATTATGTTACCGTATGCTGTTGTTTCATTTAAGGAGCTGGAAAAGGTATGTTGGGAAATCT TM-1 ACAGTACTTTACTACATTTATTATGTTACCGTATGCTGTTGTTTCATTTAAGGAGCTGGAAAAGGTATGTTGGGAAATCT**

**HJDGZ AAACACCATTTGCACACTGCCTTGCAGTCAAATATAAAAACCTTTGTCTTCATTGGGATTTAAATACCCACAATTTTTCT BYM AAACACCATTTGCACACTGCCTTGCAGTCAAATATAAAAACCTTTGTCTTCATTGGGATTTAAATACCCACAATTTTTCT CN AAACACCATTTGCACACTGCCTTGCAGTCAAATATAAAAACCTTTGTCTTCATTGGGATTTAAATACCCACAATTTTTCT NDM8 AAACACCATTTGCACACTGCCTTGCAGTCAAATATAAAAACCTTTGTCTTCATTGGGATTTAAATACCCACAATTTTTCT San4080 AAACACCATTTGCACACTGCCTTGCAGTCAAATATAAAAACCTTTGTCTTCATTGGGATTTAAATACCCACAATTTTTCT ZM24 AAACACCATTTGCACACTGCCTTGCAGTCAAATATAAAAACCTTTGTCTTCATTGGGATTTAAATACCCACAATTTTTCT ZMS35 AAACACCATTTGCACACTGCCTTGCAGTCAAATATAAAAACCTTTGTCTTCATTGGGATTTAAATACCCACAATTTTTCT Jin7 AAACACCATTTGCACACTGCCTTGCAGTCAAATATAAAAACCTTTGTCTTCATTGGGATTTAAATACCCACAATTTTTCT Jin668 AAACACCATTTGCACACTGCCTTGCAGTCAAATATAAAAACCTTTGTCTTCATTGGGATTTAAATACCCACAATTTTTCT YZ-1 AAACACCATTTGCACACTGCCTTGCAGTCAAATATAAAAACCTTTGTCTTCATTGGGATTTAAATACCCACAATTTTTCT TM-1 AAACACCATTTGCACACTGCCTTGCAGTCAAATATAAAAACCTTTGTCTTCATTGGGATTTAAATACCCACAATTTTTCT**

**HJDGZ GCATCATTGTTCTATAAAAGAATTAAATTTTTTTTCCTTTGCACTTATTCCAATGAAATAACCAAATTCATATTTCTCGT BYM GCATCATTGTTCTATAAAAGAATTAAATTTTTTTTCCTTTGCACTTATTCCAATGAAATAACCAAATTCATATTTCTCGT CN GCATCATTGTTCTATAAAAGAATTAAATTTTTTTTCCTTTGCACTTATTCCAATGAAATAACCAAATTCATATTTCTCGT NDM8 GCATCATTGTTCTATAAAAGAATTAAATTTTTTTTCCTTTGCACTTATTCCAATGAAATAACCAAATTCATATTTCTCGT San4080 GCATCATTGTTCTATAAAAGAATTAAATTTTTTTTCCTTTGCACTTATTCCAATGAAATAACCAAATTCATATTTCTCGT ZM24 GCATCATTGTTCTATAAAAGAATTAAATTTTTTTTCCTTTGCACTTATTCCAATGAAATAACCAAATTCATATTTCTCGT ZMS35 GCATCATTGTTCTATAAAAGAATTAAATTTTTTTTCCTTTGCACTCATTCCAATGAAATAACCAAATTCATATTTCTAGT Jin7 GCATCATTGTTCTATAAAAGAATTAAATTTTTTTTCCTTTGCACTCATTCCAATGAAATAACCAAATTCATATTTCTAGT Jin668 GCATCATTGTTCTATAAAAGAATTAAATTTTTTTTCCTTTGCACTCATTCCAATGAAATAACCAAATTCATATTTCTAGT YZ-1 GCATCATTGTTCTATAAAAGAATTAAATTTTTTTTCCTTTGCACTCATTCCAATGAAATAACCAAATTCATATTTCTAGT TM-1 GCATCATTGTTCTATAAAAGAATTAAATTTTTTTTCCTTTGCACTCATTCCAATGAAATAACCAAATTCATATTTCTAGT**

**HJDGZ TTTATTGCTGATGAAAAGCTAAGGGTTATTTTCTTTTTTTACAATTAGTATATTTATAGCCTGTTTAAAGCAATGATTCT BYM TTTATTGCTGATGAAAAGCTAAGGGTTATTTTCTTTTTTTACAATTAGTATATTTATAGCCTGTTTAAAGCAATGATTCT CN TTTATTGCTGATGAAAAGCTAAGGGTTATTTTCTTTTTTTACAATTAGTATATTTATAGCCTGTTTAAAGCAATGATTCT NDM8 TTTATTGCTGATGAAAAGCTAAGGGTTATTTTCTTTTTTTACAATTAGTATATTTATAGCCTGTTTAAAGCAATGATTCT San4080 TTTATTGCTGATGAAAAGCTAAGGGTTATTTTCTTTTTTTACAATTAGTATATTTATAGCCTGTTTAAAGCAATGATTCT ZM24 TTTATTGCTGATGAAAAGCTAAGGGTTATTTTCTTTTTTTACAATTAGTATATTTATAGCCTGTTTAAAGCAATGATTCT ZMS35 TTTATTGCTGATGAAAAGCTAAGGGTTATTTTCTTTTTTTACAATTAGTATATTTATAGCCTGTTTAAAGCAATGATTCT Jin7 TTTATTGCTGATGAAAAGCTAAGGGTTATTTTCTTTTTTTACAATTAGTATATTTATAGCCTGTTTAAAGCAATGATTCT Jin668 TTTATTGCTGATGAAAAGCTAAGGGTTATTTTCTTTTTTTACAATTAGTATATTTATAGCCTGTTTAAAGCAATGATTCT YZ-1 TTTATTGCTGATGAAAAGCTAAGGGTTATTTTCTTTTTTTACAATTAGTATATTTATAGCCTGTTTAAAGCAATGATTCT TM-1 TTTATTGCTGATGAAAAGCTAAGGGTTATTTTCTTTTTTTACAATTAGTATATTTATAGCCTGTTTAAAGCAATGATTCT**

**HJDGZ TACTTGTTCAGTGAACTGAAACTCAATAATCTGTGTTCAAAGCATTGTTCTGTTACTGTTGTTTTTATGCATTTACTCTT BYM TACTTGTTCAGTGAACTGAAACTCAATAATCTGTGTTCAAAGCATTGTTCTGTTACTGTTGTTTTTATGCATTTACTCTT CN TACTTGTTCAGTGAACTGAAACTCAATAATCTGTGTTCAAAGCATTGTTCTGTTACTGTTGTTTTTATGCATTTACTCTT NDM8 TACTTGTTCAGTGAACTGAAACTCAATAATCTGTGTTCAAAGCATTGTTCTGTTACTGTTGTTTTTATGCATTTACTCTT San4080 TACTTGTTCAGTGAACTGAAACTCAATAATCTGTGTTCAAAGCATTGTTCTGTTACTGTTGTTTTTATGCATTTACTCTT ZM24 TACTTGTTCAGTGAACTGAAACTCAATAATCTGTGTTCAAAGCATTGTTCTGTTACTGTTGTTTTTATGCATTTACTCTT ZMS35 TACTTGTTCAGTGAACTGAAACTCAATAATCTGTGTTCAAAGCATTGTTCTGTTACTGTTGTTTTTATGCATTTACTCTT Jin7 TACTTGTTCAGTGAACTGAAACTCAATAATCTGTGTTCAAAGCATTGTTCTGTTACTGTTGTTTTTATGCATTTACTCTT Jin668 TACTTGTTCAGTGAACTGAAACTCAATAATCTGTGTTCAAAGCATTGTTCTGTTACTGTTGTTTTTATGCATTTACTCTT YZ-1 TACTTGTTCAGTGAACTGAAACTCAATAATCTGTGTTCAAAGCATTGTTCTGTTACTGTTGTTTTTATGCATTTACTCTT TM-1 TACTTGTTCAGTGAACTGAAACTCAATAATCTGTGTTCAAAGCATTGTTCTGTTACTGTTGTTTTTATGCATTTACTCTT**

**HJDGZ TAAGCTTTTCAGCCTTGTTTTTCAGGTTTTTTAACACTGAAAATGCAAAGATCTTACTGTAAATATAATCTCTTAAAAGG BYM TAAGCTTTTCAGCCTTGTTTTTCAGGTTTTTTAACACTGAAAATGCAAAGATCTTACTGTAAATATAATCTCTTAAAAGG CN TAAGCTTTTCAGCCTTGTTTTTCAGGTTTTTTAACACTGAAAATGCAAAGATCTTACTGTAAATATAATCTCTTAAAAGG NDM8 TAAGCTTTTCAGCCTTGTTTTTCAGGTTTTTTAACACTGAAAATGCAAAGATCTTACTGTAAATATAATCTCTTAAAAGG San4080 TAAGCTTTTCAGCCTTGTTTTTCAGGTTTTTTAACACTGAAAATGCAAAGATCTTACTGTAAATATAATCTCTTAAAAGG ZM24 TAAGCTTTTCAGCCTTGTTTTTCAGGTTTTTTAACACTGAAAATGCAAAGATCTTACTGTAAATATAATCTCTTAAAAGG ZMS35 TAAGCTTTTCAGCCTTGTTTTTCAGGTTTTTTAACACTGAAAATGCAAAGATCTTACTGTAAATATAATCTCTTAAAAGG Jin7 TAAGCTTTTCAGCCTTGTTTTTCAGGTTTTTTAACACTGAAAATGCAAAGATCTTACTGTAAATATAATCTCTTAAAAGG Jin668 TAAGCTTTTCAGCCTTGTTTTTCAGGTTTTTTAACACTGAAAATGCAAAGATCTTACTGTAAATATAATCTCTTAAAAGG YZ-1 TAAGCTTTTCAGCCTTGTTTTTCAGGTTTTTTAACACTGAAAATGCAAAGATCTTACTGTAAATATAATCTCTTAAAAGG TM-1 TAAGCTTTTCAGCCTTGTTTTTCAGGTTTTTTAACACTGAAAATGCAAAGATCTTACTGTAAATATAATCTCTTAAAAGG**

**HJDGZ TGTTTTTTACCATTTGGACTTCTGGCAATTAACTGAATCTCACTAATCTTTGTTATCTTTCCAATGGTTTCACCGTTGCA BYM TGTTTTTTACCATTTGGACTTCTGGCAATTAACTGAATCTCACTAATCTTTGTTATCTTTCCAATGGTTTCACCGTTGCA CN TGTTTTTTACCATTTGGACTTCTGGCAATTAACTGAATCTCACTAATCTTTGTTATCTTTCCAATGGTTTCACCGTTGCA NDM8 TGTTTTTTACCATTTGGACTTCTGGCAATTAACTGAATCTCACTAATCTTTGTTATCTTTCCAATGGTTTCACCGTTGCA San4080 TGTTTTTTACCATTTGGACTTCTGGCAATTAACTGAATCTCACTAATCTTTGTTATCTTTCCAATGGTTTCACCGTTGCA**

**ZM24 TGTTTTTTACCATTTGGACTTCTGGCAATTAACTGAATCTCACTAATCTTTGTTATCTTTCCAATGGTTTCACCGTTGCA ZMS35 TGTTTTTTACCATTTGGACTTCTGGCAATTAACTGAATCTCACTAATCTTTGTTATCTTTCCAATGGTTTCACTGTTGCA Jin7 TGTTTTTTACCATTTGGACTTCTGGCAATTAACTGAATCTCACTAATCTTTGTTATCTTTCCAATGGTTTCACTGTTGCA Jin668 TGTTTTTTACCATTTGGACTTCTGGCAATTAACTGAATCTCACTAATCTTTGTTATCTTTCCAATGGTTTCACTGTTGCA YZ-1 TGTTTTTTACCATTTGGACTTCTGGCAATTAACTGAATCTCACTAATCTTTGTTATCTTTCCAATGGTTTCACTGTTGCA TM-1 TGTTTTTTACCATTTGGACTTCTGGCAATTAACTGAATCTCACTAATCTTTGTTATCTTTCCAATGGTTTCACTGTTGCA**

**HJDGZ TTAACCCCAAATTACTTGCAGGAAAAGCAATGTTTTTTCTTTATTTGGCTAAAAATATGCAAGTTTTTATCATTGCAGGA BYM TTAACCCCAAATTACTTGCAGGAAAAGCAATGTTTTTTCTTTATTTGGCTAAAAATATGCAAGTTTTTATCATTGCAGGA CN TTAACCCCAAATTACTTGCAGGAAAAGCAATGTTTTTTCTTTATTTGGCTAAAAATATGCAAGTTTTTATCATTGCAGGA NDM8 TTAACCCCAAATTACTTGCAGGAAAAGCAATGTTTTTTCTTTATTTGGCTAAAAATATGCAAGTTTTTATCATTGCAGGA San4080 TTAACCCCAAATTACTTGCAGGAAAAGCAATGTTTTTTCTTTATTTGGCTAAAAATATGCAAGTTTTTATCATTGCAGGA ZM24 TTAACCCCAAATTACTTGCAGGAAAAGCAATGTTTTTTCTTTATTTGGCTAAAAATATGCAAGTTTTTATCATTGCAGGA ZMS35 TTAACCCCAAATTACTTGTAGGAAAAGCAATGTTTTTTCTTTATTTGGCTAAAAATATGCAAGTTTTTATCATTGCAGGA Jin7 TTAACCCCAAATTACTTGTAGGAAAAGCAATGTTTTTTCTTTATTTGGCTAAAAATATGCAAGTTTTTATCATTGCAGGA Jin668 TTAACCCCAAATTACTTGTAGGAAAAGCAATGTTTTTTCTTTATTTGGCTAAAAATATGCAAGTTTTTATCATTGCAGGA YZ-1 TTAACCCCAAATTACTTGTAGGAAAAGCAATGTTTTTTCTTTATTTGGCTAAAAATATGCAAGTTTTTATCATTGCAGGA TM-1 TTAACCCCAAATTACTTGTAGGAAAAGCAATGTTTTTTCTTTATTTGGCTAAAAATATGCAAGTTTTTATCATTGCAGGA**

**Start of exon3**

**HJDGZ TGAATGGGTTATATCCAGGGTTTTTCAGAAGAGTGGTTCCGGCAATGGTGCAAGCAGCAGCAATGGCGGGGGAGCAAGGA BYM TGAATGGGTTATATCCAGGGTTTTTCAGAAGAGTGGTTCCGGCAATGGTGCAAGCAGCAGCAATGGCGGGGGAGCAAGGA CN TGAATGGGTTATATCCAGGGTTTTTCAGAAGAGTGGTTCCGGCAATGGTGCAAGCAGCAGCAATGGCGGGGGAGCAAGGA NDM8 TGAATGGGTTATATCCAGGGTTTTTCAGAAGAGTGGTTCCGGCAATGGTGCAAGCAGCAGCAATGGCGGGGGAGCAAGGA San4080 TGAATGGGTTATATCCAGGGTTTTTCAGAAGAGTGGTTCCGGCAATGGTGCAAGCAGCAGCAATGGCGGGGGAGCAAGGA ZM24 TGAATGGGTTATATCCAGGGTTTTTCAGAAGAGTGGTTCCGGCAATGGTGCAAGCAGCAGCAATGGCGGGGGAGCAAGGA ZMS35 TGAATGGGTTATATCCAGGGTTTTTCAGAAGAGTGGTTCCGGCAATGGTGCAAGCAGCAGCAATGGCGGCGGAGCAAGGA Jin7 TGAATGGGTTATATCCAGGGTTTTTCAGAAGAGTGGTTCCGGCAATGGTGCAAGCAGCAGCAATGGCGGCGGAGCAAGGA Jin668 TGAATGGGTTATATCCAGGGTTTTTCAGAAGAGTGGTTCCGGCAATGGTGCAAGCAGCAGCAATGGCGGCGGAGCAAGGA YZ-1 TGAATGGGTTATATCCAGGGTTTTTCAGAAGAGTGGTTCCGGCAATGGTGCAAGCAGCAGCAATGGCGGCGGAGCAAGGA TM-1 TGAATGGGTTATATCCAGGGTTTTTCAGAAGAGTGGTTCCGGCAATGGTGCAAGCAGCAGCAATGGCGGCGGAGCAAGGA**

**HJDGZ AGACTGGCCGTATGAGTGCCTCCATTGCTCTTTATCAAGAACCTAGCTCTCCTTCCTCCATCTCCCTTCCACCTCTCCTT BYM AGACTGGCCGTATGAGTGCCTCCATTGCTCTTTATCAAGAACCTAGCTCTCCTTCCTCCATCTCCCTTCCACCTCTCCTT CN AGACTGGCCGTATGAGTGCCTCCATTGCTCTTTATCAAGAACCTAGCTCTCCTTCCTCCATCTCCCTTCCACCTCTCCTT NDM8 AGACTGGCCGTATGAGTGCCTCCATTGCTCTTTATCAAGAACCTAGCTCTCCTTCCTCCATCTCCCTTCCACCTCTCCTT San4080 AGACTGGCCGTATGAGTGCCTCCATTGCTCTTTATCAAGAACCTAGCTCTCCTTCCTCCATCTCCCTTCCACCTCTCCTT ZM24 AGACTGGCCGTATGAGTGCCTCCATTGCTCTTTATCAAGAACCTAGCTCTCCTTCCTCCATCTCCCTTCCACCTCTCCTT ZMS35 AGACTGGCCGTATGAGTGCCTCCATTGCTCTTTATCAAGAACCTAGCTCTCCTTCCTCCATCTCCCTTCCACCTCTCCTT Jin7 AGACTGGCCGTATGAGTGCCTCCATTGCTCTTTATCAAGAACCTAGCTCTCCTTCCTCCATCTCCCTTCCACCTCTCCTT Jin668 AGACTGGCCGTATGAGTGCCTCCATTGCTCTTTATCAAGAACCTAGCTCTCCTTCCTCCATCTCCCTTCCACCTCTCCTT YZ-1 AGACTGGCCGTATGAGTGCCTCCATTGCTCTTTATCAAGAACCTAGCTCTCCTTCCTCCATCTCCCTTCCACCTCTCCTT TM-1 AGACTGGCCGTATGAGTGCCTCCATTGCTCTTTATCAAGAACCTAGCTCTCCTTCCTCCATCTCCCTTCCACCTCTCCTT**

**HJDGZ GATCCCACCACCACTGCCGTTTCCCTCACCGACCGTGACAGCTGCTCCTACGACAGCCATACACAATCTGAGCACGAGTC BYM GATCCCACCACCACTGCCGTTTCCCTCACCGACCGTGACAGCTGCTCCTACGACAGCCATACACAATCTGAGCACGAGTC CN GATCCCACCACCACTGCCGTTTCCCTCACCGACCGTGACAGCTGCTCCTACGACAGCCATACACAATCTGAGCACGAGTC NDM8 GATCCCACCACCACTGCCGTTTCCCTCACCGACCGTGACAGCTGCTCCTACGACAGCCATACACAATCTGAGCACGTGTC San4080 GATCCCACCACCACTGCCGTTTCCCTCACCGACCGTGACAGCTGCTCCTACGACAGCCATACACAATCTGAGCACGTGTC ZM24 GATCCCACCACCACTGCCGTTTCCCTCACCGACCGTGACAGCTGCTCCTACGACAGCCATACACAATCTGAGCACGTGTC ZMS35 GATCCCACCACCACTGCCGTTTCCCTCACCGACCGTGACAGCTGCTCCTACGACAGCCATACACAATCTGAGCACGTGTC Jin7 GATCCCACCACCACTGCCGTTTCCCTCACCGACCGTGACAGCTGCTCCTACGACAGCCATACACAATCTGAGCACGTGTC Jin668 GATCCCACCACCACTGCCGTTTCCCTCACCGACCGTGACAGCTGCTCCTACGACAGCCATACACAATCTGAGCACGTGTC YZ-1 GATCCCACCACCACTGCCGTTTCCCTCACCGACCGTGACAGCTGCTCCTACGACAGCCATACACAATCTGAGCACGTGTC TM-1 GATCCCACCACCACTGCCGTTTCCCTCACCGACCGTGACAGCTGCTCCTACGACAGCCATACACAATCTGAGCACGTGTC**

**SNP (T6400827A)**

**HJDGZ CTGTTTCTCCACCATTGCTGCTGCAGCTGCCTCCGCTGCTGCCACTTCCACAACCACCCCACATCTCTTCCACCCGGGTT BYM CTGTTTCTCCACCATTGCTGCTGCAGCTGCCTCCGCTGCTGCCACTTCCACAACCACCCCACATCTCTTCCACCCGGGTT CN CTGTTTCTCCACCATTGCTGCTGCAGCTGCCTCCGCTGCTGCCACTTCCACAACCACCCCACATCTCTTCCACCCGGGTT NDM8 CTGTTTCTCCACCATTGCTGCTGCAGCTGCCTCCGCTGCTGCCACTTCCACAACCACCCCACATCTCTTCCACCCGGGTT San4080 CTGTTTCTCCACCATTGCTGCTGCAGCTGCCTCCGCTGCTGCCACTTCCACAACCACCCCACATCTCTTCCACCCGGGTT ZM24 CTGTTTCTCCACCATTGCTGCTGCAGCTGCCTCCGCTGCTGCCACTTCCACAACCACCCCACATCTCTTCCACCCGGGTT ZMS35 CTGTTTCTCCACCATTGCTGCTGCAGCTGCCTCCGCTGCTGCCACTTCCACAACCACCCCACATCTCTTCCACCCGGGTT Jin7 CTGTTTCTCCACCATTGCTGCTGCAGCTGCCTCCGCTGCTGCCACTTCCACAACCACCCCACATCTCTTCCACCCGGGTT Jin668 CTGTTTCTCCACCATTGCTGCTGCAGCTGCCTCCGCTGCTGCCACTTCCACAACCACCCCACATCTCTTCCACCCGGGTT YZ-1 CTGTTTCTCCACCATTGCTGCTGCAGCTGCCTCCGCTGCTGCCACTTCCACAACCACCCCACATCTCTTCCACCCGGGTT TM-1 CTGTTTCTCCACCATTGCTGCTGCAGCTGCCTCCGCTGCTGCCACTTCCACAACCACCCCACATCTCTTCCACCCGGGTT**

**HJDGZ TCGACCTAGCAATGCCACCACAATCACCCCAAATGATGAACAACGGTTTTGATTCAATCTCAAGGTATTCGCGAAATCCC BYM TCGACCTAGCAATGCCACCACAATCACCCCAAATGATGAACAACGGTTTTGATTCAATCTCAAGGTATTCGCGAAATCCC**

**CN TCGACCTAGCAATGCCACCACAATCACCCCAAATGATGAACAACGGTTTTGATTCAATCTCAAGGTATTCGCGAAATCCC NDM8 TCGACCTAGCAATGCCACCACAATCACCCCAAATGATGAACAACGGTTTTGATTCAATCTCAAGGTATTCGCGAAATCCC San4080 TCGACCTAGCAATGCCACCACAATCACCCCAAATGATGAACAACGGTTTTGATTCAATCTCAAGGTATTCGCGAAATCCC ZM24 TCGACCTAGCAATGCCACCACAATCACCCCAAATGATGAACAACGGTTTTGATTCAATCTCAAGGTATTCGCGAAATCCC ZMS35 TCGACCTAGCAATGCCACCACAATCACCCCAAATGATGAACAACAGTTTTGATTCAATCTCAAGGTATTCGCGAAATCCC Jin7 TCGACCTAGCAATGCCACCACAATCACCCCAAATGATGAACAACAGTTTTGATTCAATCTCAAGGTATTCGCGAAATCCC Jin668 TCGACCTAGCAATGCCACCACAATCACCCCAAATGATGAACAACAGTTTTGATTCAATCTCAAGGTATTCGCGAAATCCC YZ-1 TCGACCTAGCAATGCCACCACAATCACCCCAAATGATGAACAACAGTTTTGATTCAATCTCAAGGTATTCGCGAAATCCC TM-1 TCGACCTAGCAATGCCACCACAATCACCCCAAATGATGAACAACAGTTTTGATTCAATCTCAAGGTATTCGCGAAATCCC**

**HJDGZ GGTGTTTCAGTATTCCCTAGCTTGAGGTCTCTACAGGAGAATTTGCAGTTCCCTTTCTTTTTCTCTCAGCCGACAATGGC BYM GGTGTTTCAGTATTCCCTAGCTTGAGGTCTCTACAGGAGAATTTGCAGTTCCCTTTCTTTTTCTCTCAGCCGACAATGGC CN GGTGTTTCAGTATTCCCTAGCTTGAGGTCTCTACAGGAGAATTTGCAGTTCCCTTTCTTTTTCTCTCAGCCGACAATGGC NDM8 GGTGTTTCAGTATTCCCTAGCTTGAGGTCTCTACAGGAGAATTTGCAGTTCCCTTTCTTTTTCTCTCAGCCGACAATGGC San4080 GGTGTTTCAGTATTCCCTAGCTTGAGGTCTCTACAGGAGAATTTGCAGTTCCCTTTCTTTTTCTCTCAGCCGACAATGGC ZM24 GGTGTTTCAGTATTCCCTAGCTTGAGGTCTCTACAGGAGAATTTGCAGTTCCCTTTCTTTTTCTCTCAGCCGACAATGGC ZMS35 GGTGTTTCAGTATTCCCTAGCTTGAGGTCTCTACAGGAGAATTTGCAGTTCCCTTTCTTTTTCTCTCAGCCGACAATGGC Jin7 GGTGTTTCAGTATTCCCTAGCTTGAGGTCTCTACAGGAGAATTTGCAGTTCCCTTTCTTTTTCTCTCAGCCGACAATGGC Jin668 GGTGTTTCAGTATTCCCTAGCTTGAGGTCTCTACAGGAGAATTTGCAGTTCCCTTTCTTTTTCTCTCAGCCGACAATGGC YZ-1 GGTGTTTCAGTATTCCCTAGCTTGAGGTCTCTACAGGAGAATTTGCAGTTCCCTTTCTTTTTCTCTCAGCCGACAATGGC TM-1 GGTGTTTCAGTATTCCCTAGCTTGAGGTCTCTACAGGAGAATTTGCAGTTCCCTTTCTTTTTCTCTCAGCCGACAATGGC**

**HJDGZ AGCAGCACCGCCACTTCACGGTGGTTCACCATTGAACTTCGGCGCTGTATCCGAGGAAGGTAACAACGGTTCCGGTGCTG BYM AGCAGCACCGCCACTTCACGGTGGTTCACCATTGAACTTCGGCGCTGTATCCGAGGAAGGTAACAACGGTTCCGGTGCTG CN AGCAGCACCGCCACTTCACGGTGGTTCACCATTGAACTTCGGCGCTGTATCCGAGGAAGGTAACAACGGTTCCGGTGCTG NDM8 AGCAGCACCGCCACTTCACGGTGGTTCACCATTGAACTTCGGCGCTGTATCCGAGGAAGGTAACAACGGTTCCGGTGCTG San4080 AGCAGCACCGCCACTTCACGGTGGTTCACCATTGAACTTCGGCGCTGTATCCGAGGAAGGTAACAACGGTTCCGGTGCTG ZM24 AGCAGCACCGCCACTTCACGGTGGTTCACCATTGAACTTCGGCGCTGTATCCGAGGAAGGTAACAACGGTTCCGGTGCTG ZMS35 AGCAGCACCGCCACTTCACGGTGGTTCACCATTGAACTTCGGCGCTGTATCCGAGGAAGGTAACAACGGTTCCGGTGCTG Jin7 AGCAGCACCGCCACTTCACGGTGGTTCACCATTGAACTTCGGCGCTGTATCCGAGGAAGGTAACAACGGTTCCGGTGCTG Jin668 AGCAGCACCGCCACTTCACGGTGGTTCACCATTGAACTTCGGCGCTGTATCCGAGGAAGGTAACAACGGTTCCGGTGCTG YZ-1 AGCAGCACCGCCACTTCACGGTGGTTCACCATTGAACTTCGGCGCTGTATCCGAGGAAGGTAACAACGGTTCCGGTGCTG TM-1 AGCAGCACCGCCACTTCACGGTGGTTCACCATTGAACTTCGGCGCTGTATCCGAGGAAGGTAACAACGGTTCCGGTGCTG**

**HJDGZ GTGCTAAGATATCCATTGGTCCATCTGAGTTCGATTGCATGTGGACTTACTGATCAAACTACAACTCGAGGAATATTTTT BYM GTGCTAAGATATCCATTGGTCCATCTGAGTTCGATTGCATGTGGACTTACTGATCAAACTACAACTCGAGGAATATTTTT CN GTGCTAAGATATCCATTGGTCCATCTGAGTTCGATTGCATGTGGACTTACTGATCAAACTACAACTCGAGGAATATTTTT NDM8 GTGCTAAGATATCCATTGGTCCATCTGAGTTCGATTGCATGTGGACTTACTGATCAAACTACAACTCGAGGAATATTTTT San4080 GTGCTAAGATATCCATTGGTCCATCTGAGTTCGATTGCATGTGGACTTACTGATCAAACTACAACTCGAGGAATATTTTT ZM24 GTGCTAAGATATCCATTGGTCCATCTGAGTTCGATTGCATGTGGACTTACTGATCAAACTACAACTCGAGGAATATTTTT ZMS35 GTGCTAAGATATCCATTGGTCAATCTGAGTTCGATTGCATGTGGACTTACTGATCAAACTACAACTCGAGGAATATTTTT Jin7 GTGCTAAGATATCCATTGGTCAATCTGAGTTCGATTGCATGTGGACTTACTGATCAAACTACAACTCGAGGAATATTTTT Jin668 GTGCTAAGATATCCATTGGTCAATCTGAGTTCGATTGCATGTGGACTTACTGATCAAACTACAACTCGAGGAATATTTTT YZ-1 GTGCTAAGATATCCATTGGTCAATCTGAGTTCGATTGCATGTGGACTTACTGATCAAACTACAACTCGAGGAATATTTTT TM-1 GTGCTAAGATATCCATTGGTCAATCTGAGTTCGATTGCATGTGGACTTACTGATCAAACTACAACTCGAGGAATATTTTT**

**Stop codon**

**HJDGZ CAGCCATTAATATGTGTGTGTGTGTGGCTTTTTGAAAAGAACTTTTTCTTCAAATTATCCAACTATGTTGTCATTAGGAG BYM CAGCCATTAATATGTGTGTGTGTGTGGCTTTTTGAAAAGAACTTTTTCTTCAAATTATCCAACTATGTTGTCATTAGGAG CN CAGCCATTAATATGTGTGTGTGTGTGGCTTTTTGAAAAGAACTTTTTCTTCAAATTATCCAACTATGTTGTCATTAGGAG NDM8 CAGCCATTAATATGTGTGTGTGTGTGGCTTTTTGAAAAGAACTTTTTCTTCAAATTATCCAACTATGTTGTCATTAGGAG San4080 CAGCCATTAATATGTGTGTGTGTGTGGCTTTTTGAAAAGAACTTTTTCTTCAAATTATCCAACTATGTTGTCATTAGGAG ZM24 CAGCCATTAATATGTGTGTGTGTGTGGCTTTTTGAAAAGAACTTTTTCTTCAAATTATCCAACTATGTTGTCATTAGGAG ZMS35 CAGCCATTAATATGTGTGTGTGT--GGCTTTTTGAAAAGAACTTTTTCTTCAAATTATCCAACTATGTTGTCATTAGGAG Jin7 CAGCCATTAATATGTGTGTGTGT--GGCTTTTTGAAAAGAACTTTTTCTTCAAATTATCCAACTATGTTGTCATTAGGAG Jin668 CAGCCATTAATATGTGTGTGTGT--GGCTTTTTGAAAAGAACTTTTTCTTCAAATTATCCAACTATGTTGTCATTAGGAG YZ-1 CAGCCATTAATATGTGTGTGTGT--GGCTTTTTGAAAAGAACTTTTTCTTCAAATTATCCAACTATGTTGTCATTAGGAG TM-1 CAGCCATTAATATGTGTGTGTGT--GGCTTTTTGAAAAGAACTTTTTCTTCAAATTATCCAACTATGTTGTCATTAGGAG**

**HJDGZ TAGGCGTAGAAAAGGACGGGTGATTAGTGATTAACGTCAGCTAAGATTTCCCTTTCATGTCTGTAAGATTACCATTAATT BYM TAGGCGTAGAAAAGGACGGGTGATTAGTGATTAACGTCAGCTAAGATTTCCCTTTCATGTCTGTAAGATTACCATTAATT CN TAGGCGTAGAAAAGGACGGGTGATTAGTGATTAACGTCAGCTAAGATTTCCCTTTCATGTCTGTAAGATTACCATTAATT NDM8 TAGGCGTAGAAAAGGACGGGTGATTAGTGATTAACGTCAGCTAAGATTTCCCTTTCATGTCTGTAAGATTACCATTAATT San4080 TAGGCGTAGAAAAGGACGGGTGATTAGTGATTAACGTCAGCTAAGATTTCCCTTTCATGTCTGTAAGATTACCATTAATT ZM24 TAGGCGTAGAAAAGGACGGGTGATTAGTGATTAACGTCAGCTAAGATTTCCCTTTCATGTCTGTAAGATTACCATTAATT ZMS35 TAGGCGTAGAAAAGGACGGGTGATTAGTGATTAACGTCAGCTAAGATTTCCCTTTCATGTCTGTAAGATTACCATTAATT Jin7 TAGGCGTAGAAAAGGACGGGTGATTAGTGATTAACGTCAGCTAAGATTTCCCTTTCATGTCTGTAAGATTACCATTAATT Jin668 TAGGCGTAGAAAAGGACGGGTGATTAGTGATTAACGTCAGCTAAGATTTCCCTTTCATGTCTGTAAGATTACCATTAATT YZ-1 TAGGCGTAGAAAAGGACGGGTGATTAGTGATTAACGTCAGCTAAGATTTCCCTTTCATGTCTGTAAGATTACCATTAATT TM-1 TAGGCGTAGAAAAGGACGGGTGATTAGTGATTAACGTCAGCTAAGATTTCCCTTTCATGTCTGTAAGATTACCATTAATT**

**HJDGZ AATCTTAGTTTTGCTGGTTAAAATG---GTGACTTATACATGGCTGATAAG---TATGTTTCAACTATGAGATCACAACA BYM AATCTTAGTTTTGCTGGTTAAAATG---GTGACTTATACATGGCTGATAAG---TATGTTTCAACTATGAGATCACAACA**

**CN AATCTTAGTTTTGCTGGTTAAAATG---GTGACTTATACATGGCTGATAAG---TATGTTTCAACTATGAGATCACAACA NDM8 AATCTTAGTTTTGCTGGTTAAAATG---GTGACTTATACATGGCTGATAAG---TATGTTTCAACTATGAGATCACAACA San4080 AATCTTAGTTTTGCTGGTTAAAATG---GTGACTTATACATGGCTGATAAG---TATGTTTCAACTATGAGATCACAACA ZM24 AATCTTAGTTTTGCTGGTTAAAATG---GTGACTTATACATGGCTGATAAG---TATGTTTCAACTATGAGATCACAACA ZMS35 AATCTTAGTTTTGCTGGTTAAAATGATGGTGACTTATACATGGCTGATAAGAAGTATGTTTCAACTATGAGATCACAACA Jin7 AATCTTAGTTTTGCTGGTTAAAATGATGGTGACTTATACATGGCTGATAAGAAGTATGTTTCAACTATGAGATCACAACA Jin668 AATCTTAGTTTTGCTGGTTAAAATGATGGTGACTTATACATGGCTGATAAGAAGTATGTTTCAACTATGAGATCACAACA YZ-1 AATCTTAGTTTTGCTGGTTAAAATGATGGTGACTTATACATGGCTGATAAGAAGTATGTTTCAACTATGAGATCACAACA TM-1 AATCTTAGTTTTGCTGGTTAAAATGATGGTGACTTATACATGGCTGATAAGAAGTATGTTTCAACTATGAGATCACAACA**

**HJDGZ GTCAATTTACCCCTTCCTCTTTTTTCCCCTTTCCCCGTCCAAATCTAATTTTCTTTTCCCGGAAAAGATTCATCTCTGGT BYM GTCAATTTACCCCTTCCTCTTTTTTCCCCTTTCCCCGTCCAAATCTAATTTTCTTTTCCCGGAAAAGATTCATCTCTGGT CN GTCAATTTACCCCTTCCTCTTTTTTCCCCTTTCCCCGTCCAAATCTAATTTTCTTTTCCCGGAAAAGATTCATCTCTGGT NDM8 GTCAATTTACCCCTTCCTCTTTTTTCCCCTTTCCCCGTCCAAATCTAATTTTCTTTTCCCGGAAAAGATTCATCTCTGGT San4080 GTCAATTTACCCCTTCCTCTTTTTTCCCCTTTCCCCGTCCAAATCTAATTTTCTTTTCCCGGAAAAGATTCATCTCTGGT ZM24 GTCAATTTACCCCTTCCTCTTTTTTCCCCTTTCCCCGTCCAAATCTAATTTTCTTTTCCCGGAAAAGATTCATCTCTGGT ZMS35 GTCAATTTACCCCTTCCTCTTTTTTCCCCTTTCCCCGTCCAAATCTAATTTTCTTTTCCCGGAAAAGATTCATCTCTGGT Jin7 GTCAATTTACCCCTTCCTCTTTTTTCCCCTTTCCCCGTCCAAATCTAATTTTCTTTTCCCGGAAAAGATTCATCTCTGGT Jin668 GTCAATTTACCCCTTCCTCTTTTTTCCCCTTTCCCCGTCCAAATCTAATTTTCTTTTCCCGGAAAAGATTCATCTCTGGT YZ-1 GTCAATTTACCCCTTCCTCTTTTTTCCCCTTTCCCCGTCCAAATCTAATTTTCTTTTCCCGGAAAAGATTCATCTCTGGT TM-1 GTCAATTTACCCCTTCCTCTTTTTTCCCCTTTCCCCGTCCAAATCTAATTTTCTTTTCCCGGAAAAGATTCATCTCTGGT**

**HJDGZ TTCTTTTTGGGTTCTAAGAAAATGACTTACTATTTCATCAAACACAAAGAATCCCATAAATAACCTTTTAAAAGGTTTTC BYM TTCTTTTTGGGTTCTAAGAAAATGACTTACTATTTCATCAAACACAAAGAATCCCATAAATAACCTTTTAAAAGGTTTTC CN TTCTTTTTGGGTTCTAAGAAAATGACTTACTATTTCATCAAACACAAAGAATCCCATAAATAACCTTTTAAAAGGTTTTC NDM8 TTCTTTTTGGGTTCTAAGAAAATGACTTACTATTTCATCAAACACAAAGAATCCCATAAATAACCTTTTAAAAGGTTTTC San4080 TTCTTTTTGGGTTCTAAGAAAATGACTTACTATTTCATCAAACACAAAGAATCCCATAAATAACCTTTTAAAAGGTTTTC ZM24 TTCTTTTTGGGTTCTAAGAAAATGACTTACTATTTCATCAAACACAAAGAATCCCATAAATAACCTTTTAAAAGGTTTTC ZMS35 TTCTTTTTGGGTTCTAAGAAAATGACTTACTATTTCATCAAACACAAAGAATCCCATAAATAACCTTTTAAAAGGTTTTC Jin7 TTCTTTTTGGGTTCTAAGAAAATGACTTACTATTTCATCAAACACAAAGAATCCCATAAATAACCTTTTAAAAGGTTTTC Jin668 TTCTTTTTGGGTTCTAAGAAAATGACTTACTATTTCATCAAACACAAAGAATCCCATAAATAACCTTTTAAAAGGTTTTC YZ-1 TTCTTTTTGGGTTCTAAGAAAATGACTTACTATTTCATCAAACACAAAGAATCCCATAAATAACCTTTTAAAAGGTTTTC TM-1 TTCTTTTTGGGTTCTAAGAAAATGACTTACTATTTCATCAAACACAAAGAATCCCATAAATAACCTTTTAAAAGGTTTTC**

**HJDGZ CTTTTTTTTTTTTTTTACTTCTTACATTAAATGTTTCAAAAGCAAAGGACTTTTATCTAACTAATGCTCATAAATGGGCC BYM CTTTTTTTTTTTTTTTACTTCTTACATTAAATGTTTCAAAAGCAAAGGACTTTTATCTAACTAATGCTCATAAATGGGCC CN CTTTTTTTTTTTTTTTACTTCTTACATTAAATGTTTCAAAAGCAAAGGACTTTTATCTAACTAATGCTCATAAATGGGCC NDM8 CTTTTTTTTTTTTTTTACTTCTTACATTAAATGTTTCAAAAGCAAAGGACTTTTATCTAACTAATGCTCATAAATGGGCC San4080 CTTTTTTTTTTTTTTTACTTCTTACATTAAATGTTTCAAAAGCAAAGGACTTTTATCTAACTAATGCTCATAAATGGGCC ZM24 CTTTTTTTTTTTTTTTACTTCTTACATTAAATGTTTCAAAAGCAAAGGACTTTTATCTAACTAATGCTCATAAATGGGCC ZMS35 CTTTTTTTTTTTTTTTACTTCTTACATTAAATTTTTCAAAAGCAAAGGACTTTTATCTAACTAATGCTCATAAATGGGCC Jin7 CTTTTTTTTTTTTTTTACTTCTTACATTAAATTTTTCAAAAGCAAAGGACTTTTATCTAACTAATGCTCATAAATGGGCC Jin668 CTTTTTTTTTTTTTTTACTTCTTACATTAAATTTTTCAAAAGCAAAGGACTTTTATCTAACTAATGCTCATAAATGGGCC YZ-1 CTTTTTTTTTTTTTTTACTTCTTACATTAAATTTTTCAAAAGCAAAGGACTTTTATCTAACTAATGCTCATAAATGGGCC TM-1 CTTTTTTTTTTTTTTTACTTCTTACATTAAATTTTTCAAAAGCAAAGGACTTTTATCTAACTAATGCTCATAAATGGGCC**

**HJDGZ GTTGAATTAAGTTTGTGGGGTACTGGAAAGTAGAGCAAGGAAAAAAATTTGAAAATGGATCCTTTTATGAGAAGTAAAAA BYM GTTGAATTAAGTTTGTGGGGTACTGGAAAGTAGAGCAAGGAAAAAAATTTGAAAATGGATCCTTTTATGAGAAGTAAAAA CN GTTGAATTAAGTTTGTGGGGTACTGGAAAGTAGAGCAAGGAAAAAAATTTGAAAATGGATCCTTTTATGAGAAGTAAAAA NDM8 GTTGAATTAAGTTTGTGGGGTACTGGAAAGTAGAGCAAGGAAAAAAATTTGAAAATGGATCCTTTTATGAGAAGTAAAAA San4080 GTTGAATTAAGTTTGTGGGGTACTGGAAAGTAGAGCAAGGAAAAAAATTTGAAAATGGATCCTTTTATGAGAAGTAAAAA ZM24 GTTGAATTAAGTTTGTGGGGTACTGGAAAGTAGAGCAAGGAAAAAAATTTGAAAATGGATCCTTTTATGAGAAGTAAAAA ZMS35 GTTGAATTAAGTTTGTGGGGTACTGGAAAGTAGAGCAAGGAAAAAAATTTGAAAATGGATCCTTTTATGAGAAGTAAAAA Jin7 GTTGAATTAAGTTTGTGGGGTACTGGAAAGTAGAGCAAGGAAAAAAATTTGAAAATGGATCCTTTTATGAGAAGTAAAAA Jin668 GTTGAATTAAGTTTGTGGGGTACTGGAAAGTAGAGCAAGGAAAAAAATTTGAAAATGGATCCTTTTATGAGAAGTAAAAA YZ-1 GTTGAATTAAGTTTGTGGGGTACTGGAAAGTAGAGCAAGGAAAAAAATTTGAAAATGGATCCTTTTATGAGAAGTAAAAA TM-1 GTTGAATTAAGTTTGTGGGGTACTGGAAAGTAGAGCAAGGAAAAAAATTTGAAAATGGATCCTTTTATGAGAAGTAAAAA**

**HJDGZ AAATAGTGAAATAAATTTAAAATTTTATATAAAAATATTTTCTTTATGATTCCTAATTAAAAAAGAGTCCATATTGATTC BYM AAATAGTGAAATAAATTTAAAATTTTATATAAAAATATTTTCTTTATGATTCCTAATTAAAAAAGAGTCCATATTGATTC CN AAATAGTGAAATAAATTTAAAATTTTATATAAAAATATTTTCTTTATGATTCCTAATTAAAAAAGAGTCCATATTGATTC NDM8 AAATAGTGAAATAAATTTAAAATTTTATATAAAAATATTTTCTTTATGATTCCTAATTAAAAAAGAGTCCATATTGATTC San4080 AAATAGTGAAATAAATTTAAAATTTTATATAAAAATATTTTCTTTATGATTCCTAATTAAAAAAGAGTCCATATTGATTC ZM24 AAATAGTGAAATAAATTTAAAATTTTATATAAAAATATTTTCTTTATGATTCCTAATTAAAAAAGAGTCCATATTGATTC ZMS35 AAATAGTGAAATAAATTTAAAATTTTATATAAAAATATTTTCTTTATGATTCCTAATTAAAAAAGAGTCCATATTGATTC Jin7 AAATAGTGAAATAAATTTAAAATTTTATATAAAAATATTTTCTTTATGATTCCTAATTAAAAAAGAGTCCATATTGATTC Jin668 AAATAGTGAAATAAATTTAAAATTTTATATAAAAATATTTTCTTTATGATTCCTAATTAAAAAAGAGTCCATATTGATTC YZ-1 AAATAGTGAAATAAATTTAAAATTTTATATAAAAATATTTTCTTTATGATTCCTAATTAAAAAAGAGTCCATATTGATTC TM-1 AAATAGTGAAATAAATTTAAAATTTTATATAAAAATATTTTCTTTATGATTCCTAATTAAAAAAGAGTCCATATTGATTC**

**HJDGZ ATATTCCTTATAAAATAGGATTAAAAAGAAGAGAGTAAAATTTGATTCCTGCTCTCTCCAAATTGTCAAAATTGAGCTTG BYM** **ATATTCCTTATAAAATAGGATTAAAAAGAAGAGAGTAAAATTTGATTCCTGCTCTCTCCAAATTGTCAAAATTGAGCTTG CN** **ATATTCCTTATAAAATAGGATTAAAAAGAAGAGAGTAAAATTTGATTCCTGCTCTCTCCAAATTGTCAAAATTGAGCTTG**

**NDM8 ATATTCCTTATAAAATAGGATTAAAAAGAAGAGAGTAAAATTTGATTCCTGCTCTCTCCAAATTGTCAAAATTGAGCTTG San4080 ATATTCCTTATAAAATAGGATTAAAAAGAAGAGAGTAAAATTTGATTCCTGCTCTCTCCAAATTGTCAAAATTGAGCTTG ZM24 ATATTCCTTATAAAATAGGATTAAAAAGAAGAGAGTAAAATTTGATTCCTGCTCTCTCCAAATTGTCAAAATTGAGCTTG ZMS35 ATATTCCTTATAAAATAGGATTAAAAAGAAGAGAGTAAAATTTGATTCCTGCTCTCTCCAAATTGTCAAAATTGAGCTTG Jin7 ATATTCCTTATAAAATAGGATTAAAAAGAAGAGAGTAAAATTTGATTCCTGCTCTCTCCAAATTGTCAAAATTGAGCTTG Jin668 ATATTCCTTATAAAATAGGATTAAAAAGAAGAGAGTAAAATTTGATTCCTGCTCTCTCCAAATTGTCAAAATTGAGCTTG YZ-1 ATATTCCTTATAAAATAGGATTAAAAAGAAGAGAGTAAAATTTGATTCCTGCTCTCTCCAAATTGTCAAAATTGAGCTTG TM-1 ATATTCCTTATAAAATAGGATTAAAAAGAAGAGAGTAAAATTTGATTCCTGCTCTCTCCAAATTGTCAAAATTGAGCTTG**

**HJDGZ TTTGATCACATGGGAGCTAAGATTGTGAGGTGTGAGTTTAGGGTAGTGGAGACTCAGAAAGGACCATTGCCCTATTTGTG BYM TTTGATCACATGGGAGCTAAGATTGTGAGGTGTGAGTTTAGGGTAGTGGAGACTCAGAAAGGACCATTGCCCTATTTGTG CN TTTGATCACATGGGAGCTAAGATTGTGAGGTGTGAGTTTAGGGTAGTGGAGACTCAGAAAGGACCATTGCCCTATTTGTG NDM8 TTTGATCACATGGGAGCTAAGATTGTGAGGTGTGAGTTTAGGGTAGTGGAGACTCAGAAAGGACCATTGCCCTATTTGTG San4080 TTTGATCACATGGGAGCTAAGATTGTGAGGTGTGAGTTTAGGGTAGTGGAGACTCAGAAAGGACCATTGCCCTATTTGTG ZM24 TTTGATCACATGGGAGCTAAGATTGTGAGGTGTGAGTTTAGGGTAGTGGAGACTCAGAAAGGACCATTGCCCTATTTGTG ZMS35 TTTGATCACATGGGAGCTAAGATTGTGAGGTGTGAGTTTAGGGTAGTGGAGACTCAGAAAGGACCATTGCCCTATTTGTG Jin7 TTTGATCACATGGGAGCTAAGATTGTGAGGTGTGAGTTTAGGGTAGTGGAGACTCAGAAAGGACCATTGCCCTATTTGTG Jin668 TTTGATCACATGGGAGCTAAGATTGTGAGGTGTGAGTTTAGGGTAGTGGAGACTCAGAAAGGACCATTGCCCTATTTGTG YZ-1 TTTGATCACATGGGAGCTAAGATTGTGAGGTGTGAGTTTAGGGTAGTGGAGACTCAGAAAGGACCATTGCCCTATTTGTG TM-1 TTTGATCACATGGGAGCTAAGATTGTGAGGTGTGAGTTTAGGGTAGTGGAGACTCAGAAAGGACCATTGCCCTATTTGTG**

**HJDGZ GAAGGGACATATTTATTGCACTTCTGTTAACATTGTACCCTTTCATGTCAACATATGATATTGGAAGGGGAAAGTAAAGG BYM GAAGGGACATATTTATTGCACTTCTGTTAACATTGTACCCTTTCATGTCAACATATGATATTGGAAGGGGAAAGTAAAGG CN GAAGGGACATATTTATTGCACTTCTGTTAACATTGTACCCTTTCATGTCAACATATGATATTGGAAGGGGAAAGTAAAGG NDM8 GAAGGGACATATTTATTGCACTTCTGTTAACATTGTACCCTTTCATGTCAACATATGATATTGGAAGGGGAAAGTAAAGG San4080 GAAGGGACATATTTATTGCACTTCTGTTAACATTGTACCCTTTCATGTCAACATATGATATTGGAAGGGGAAAGTAAAGG ZM24 GAAGGGACATATTTATTGCACTTCTGTTAACATTGTACCCTTTCATGTCAACATATGATATTGGAAGGGGAAAGTAAAGG ZMS35 GAAGGGACATATTTATTGCACTTCTGTTAACATTGTACCCTTTCATGTCAACATATGATATTGGAAGGGGAAAGTAAAGG Jin7 GAAGGGACATATTTATTGCACTTCTGTTAACATTGTACCCTTTCATGTCAACATATGATATTGGAAGGGGAAAGTAAAGG Jin668 GAAGGGACATATTTATTGCACTTCTGTTAACATTGTACCCTTTCATGTCAACATATGATATTGGAAGGGGAAAGTAAAGG YZ-1 GAAGGGACATATTTATTGCACTTCTGTTAACATTGTACCCTTTCATGTCAACATATGATATTGGAAGGGGAAAGTAAAGG TM-1 GAAGGGACATATTTATTGCACTTCTGTTAACATTGTACCCTTTCATGTCAACATATGATATTGGAAGGGGAAAGTAAAGG**

**HJDGZ TACATAAAATAATGGTGTCCCAACTTCTTCTTTTTCTCATTTTGCACCATTATTTATTTTATTCACTTCTTAGTCATATT BYM TACATAAAATAATGGTGTCCCAACTTCTTCTTTTTCTCATTTTGCACCATTATTTATTTTATTCACTTCTTAGTCATATT CN TACATAAAATAATGGTGTCCCAACTTCTTCTTTTTCTCATTTTGCACCATTATTTATTTTATTCACTTCTTAGTCATATT NDM8 TACATAAAATAATGGTGTCCCAACTTCTTCTTTTTCTCATTTTGCACCATTATTTATTTTATTCACTTCTTAGTCATATT San4080 TACATAAAATAATGGTGTCCCAACTTCTTCTTTTTCTCATTTTGCACCATTATTTATTTTATTCACTTCTTAGTCATATT ZM24 TACATAAAATAATGGTGTCCCAACTTCTTCTTTTTCTCATTTTGCACCATTATTTATTTTATTCACTTCTTAGTCATATT ZMS35 TACATAAAATAATGGTGTCCCAACTTCTTCTTTTTCTCATTTTGCACCATTATTTATTTTATTCACTTCTTAGTCATATT Jin7 TACATAAAATAATGGTGTCCCAACTTCTTCTTTTTCTCATTTTGCACCATTATTTATTTTATTCACTTCTTAGTCATATT Jin668 TACATAAAATAATGGTGTCCCAACTTCTTCTTTTTCTCATTTTGCACCATTATTTATTTTATTCACTTCTTAGTCATATT YZ-1 TACATAAAATAATGGTGTCCCAACTTCTTCTTTTTCTCATTTTGCACCATTATTTATTTTATTCACTTCTTAGTCATATT TM-1 TACATAAAATAATGGTGTCCCAACTTCTTCTTTTTCTCATTTTGCACCATTATTTATTTTATTCACTTCTTAGTCATATT**

**HJDGZ AGTGCATTCACAATGTAAGATTATACATATTTGCATCTACATGAATATCTCTTT--GTGTGTGTGTGTTATTTTTCCATT BYM AGTGCATTCACAATGTAAGATTATACATATTTGCATCTACATGAATATCTCTTT--GTGTGTGTGTGTTATTTTTCCATT CN AGTGCATTCACAATGTAAGATTATACATATTTGCATCTACATGAATATCTCTTT--GTGTGTGTGTGTTATTTTTCCATT NDM8 AGTGCATTCACAATGTAAGATTATACATATTTGCATCTACATGAATATCTCTTT--GTGTGTGTGTGTTATTTTTCCATT San4080 AGTGCATTCACAATGTAAGATTATACATATTTGCATCTACATGAATATCTCTTT--GTGTGTGTGTGTTATTTTTCCATT ZM24 AGTGCATTCACAATGTAAGATTATACATATTTGCATCTACATGAATATCTCTTT--GTGTGTGTGTGTTATTTTTCCATT ZMS35 AGTGCATTCACAATGTAAGATTATACATATTTGCATCTACATGAATATTTCTTTTTGTGTGTGTGTGTTATTTTTCCATT Jin7 AGTGCATTCACAATGTAAGATTATACATATTTGCATCTACATGAATATTTCTTTTTGTGTGTGTGTGTTATTTTTCCATT Jin668 AGTGCATTCACAATGTAAGATTATACATATTTGCATCTACATGAATATTTCTTTTTGTGTGTGTGTGTTATTTTTCCATT YZ-1 AGTGCATTCACAATGTAAGATTATACATATTTGCATCTACATGAATATTTCTTTTTGTGTGTGTGTGTTATTTTTCCATT TM-1 AGTGCATTCACAATGTAAGATTATACATATTTGCATCTACATGAATATTTCTTTTTGTGTGTGTGTGTTATTTTTCCATT**

**HJDGZ ATTAAAGACAAAATATTTTATTATGGTAATTAATGAAATAAATTTAATTTGTGGTTTGTTTCTTTGTTGTGAAAATTCTA BYM ATTAAAGACAAAATATTTTATTATGGTAATTAATGAAATAAATTTAATTTGTGGTTTGTTTCTTTGTTGTGAAAATTCTA CN ATTAAAGACAAAATATTTTATTATGGTAATTAATGAAATAAATTTAATTTGTGGTTTGTTTCTTTGTTGTGAAAATTCTA NDM8 ATTAAAGACAAAATATTTTATTATGGTAATTAATGAAATAAATTTAATTTGTGGTTTGTTTCTTTGTTGTGAAAATTCTA San4080 ATTAAAGACAAAATATTTTATTATGGTAATTAATGAAATAAATTTAATTTGTGGTTTGTTTCTTTGTTGTGAAAATTCTA ZM24 ATTAAAGACAAAATATTTTATTATGGTAATTAATGAAATAAATTTAATTTGTGGTTTGTTTCTTTGTTGTGAAAATTCTA ZMS35 ATTAAAGACAAAATATTTTATTATGGTAATTAATGAAATAAATTTAATTTGTGGTTTGTTTCTTTGTTGTGAAAATTCTA Jin7 ATTAAAGACAAAATATTTTATTATGGTAATTAATGAAATAAATTTAATTTGTGGTTTGTTTCTTTGTTGTGAAAATTCTA Jin668 ATTAAAGACAAAATATTTTATTATGGTAATTAATGAAATAAATTTAATTTGTGGTTTGTTTCTTTGTTGTGAAAATTCTA YZ-1 ATTAAAGACAAAATATTTTATTATGGTAATTAATGAAATAAATTTAATTTGTGGTTTGTTTCTTTGTTGTGAAAATTCTA TM-1 ATTAAAGACAAAATATTTTATTATGGTAATTAATGAAATAAATTTAATTTGTGGTTTGTTTCTTTGTTGTGAAAATTCTA**

**HJDGZ GTATTGATTAATAATACAAGATAATTAAGAATATTTAATTTATTCTTTTCTTTTTCATATTAATAAACCCTTGTATTAGT BYM** **GTATTGATTAATAATACAAGATAATTAAGAATATTTAATTTATTCTTTTCTTTTTCATATTAATAAACCCTTGTATTAGT CN** **GTATTGATTAATAATACAAGATAATTAAGAATATTTAATTTATTCTTTTCTTTTTCATATTAATAAACCCTTGTATTAGT NDM8 GTATTGATTAATAATACAAGATAATTAAGAATATTTAATTTATTCTTTTCTTTTTCATATTAATAAACCCTTGTATTAGT**

**San4080 GTATTGATTAATAATACAAGATAATTAAGAATATTTAATTTATTCTTTTCTTTTTCATATTAATAAACCCTTGTATTAGT ZM24 GTATTGATTAATAATACAAGATAATTAAGAATATTTAATTTATTCTTTTCTTTTTCATATTAATAAACCCTTGTATTAGT ZMS35 GTATTGATTAATAATACAAGATAATTAAGAATATTTAATTTATTCTTTTCATTTTCATATTAATAAACCCTTGTATTAGT Jin7 GTATTGATTAATAATACAAGATAATTAAGAATATTTAATTTATTCTTTTCATTTTCATATTAATAAACCCTTGTATTAGT Jin668 GTATTGATTAATAATACAAGATAATTAAGAATATTTAATTTATTCTTTTCATTTTCATATTAATAAACCCTTGTATTAGT YZ-1 GTATTGATTAATAATACAAGATAATTAAGAATATTTAATTTATTCTTTTCATTTTCATATTAATAAACCCTTGTATTAGT TM-1 GTATTGATTAATAATACAAGATAATTAAGAATATTTAATTTATTCTTTTCATTTTCATATTAATAAACCCTTGTATTAGT**

**HJDGZ CAAATAATCTACTTTTGCTTTGCCCTAAATTTGGCATTACTCATTTAGAAGAAAGTGTTAGGGTAGAAGTAAATATCCTT BYM CAAATAATCTACTTTTGCTTTGCCCTAAATTTGGCATTACTCATTTAGAAGAAAGTGTTAGGGTAGAAGTAAATATCCTT CN CAAATAATCTACTTTTGCTTTGCCCTAAATTTGGCATTACTCATTTAGAAGAAAGTGTTAGGGTAGAAGTAAATATCCTT NDM8 CAAATAATCTACTTTTGCTTTGCCCTAAATTTGGCATTACTCATTTAGAAGAAAGTGTTAGGGTAGAAGTAAATATCCTT San4080 CAAATAATCTACTTTTGCTTTGCCCTAAATTTGGCATTACTCATTTAGAAGAAAGTGTTAGGGTAGAAGTAAATATCCTT ZM24 CAAATAATCTACTTTTGCTTTGCCCTAAATTTGGCATTACTCATTTAGAAGAAAGTGTTAGGGTAGAAGTAAATATCCTT ZMS35 CAAATAATCTACTTTTGCTTTGCCCTAAATTTGGCATTACTCATTTAGAAGAAAGTGTTAGGATAGAAGTAAATATCCTT Jin7 CAAATAATCTACTTTTGCTTTGCCCTAAATTTGGCATTACTCATTTAGAAGAAAGTGTTAGGATAGAAGTAAATATCCTT Jin668 CAAATAATCTACTTTTGCTTTGCCCTAAATTTGGCATTACTCATTTAGAAGAAAGTGTTAGGATAGAAGTAAATATCCTT YZ-1 CAAATAATCTACTTTTGCTTTGCCCTAAATTTGGCATTACTCATTTAGAAGAAAGTGTTAGGATAGAAGTAAATATCCTT TM-1 CAAATAATCTACTTTTGCTTTGCCCTAAATTTGGCATTACTCATTTAGAAGAAAGTGTTAGGATAGAAGTAAATATCCTT**

**HJDGZ BYM CN**

**NDM8 San4080 ZM24**

**ZMS35 Jin7**

**Jin668 YZ-1 TM-1**

**TAAAATAATTATTTATTTTATTTTTAATAATAATCTTTTTT TAAAATAATTATTTATTTTATTTTTAATAATAATCTTTTTT TAAAATAATTATTTATTTTATTTTTAATAATAATCTTTTTT TAAAATAATTATTTATTTTATTTTTAATAATAATCTTTTTT TAAAATAATTATTTATTTTATTTTTAATAATAATCTTTTTT TAAAATAATTATTTATTTTATTTTTAATAATAATCTTTTTT TAAAATAATTATTTATTTTATTTTTAATAATAATCTTTTTT TAAAATAATTATTTATTTTATTTTTAATAATAATCTTTTTT TAAAATAATTATTTATTTTATTTTTAATAATAATCTTTTTT TAAAATAATTATTTATTTTATTTTTAATAATAATCTTTTTT TAAAATAATTATTTATTTTATTTTTAATAATAATCTTTTTT**
