# Supplementary material for: GhSBI1, a CUP‐SHAPED COTYLEDON 2 homologue, modulates branch internode elongation in cotton
Source: Plant Biotechnol J. 2024 Jul 26;22(11):3175–93. doi: 10.1111/pbi.14439 (PMC11500989; doi:10.1111/pbi.14439)
Supplement: Supplementary file 3 [file PBI-22-3175-s001.docx]

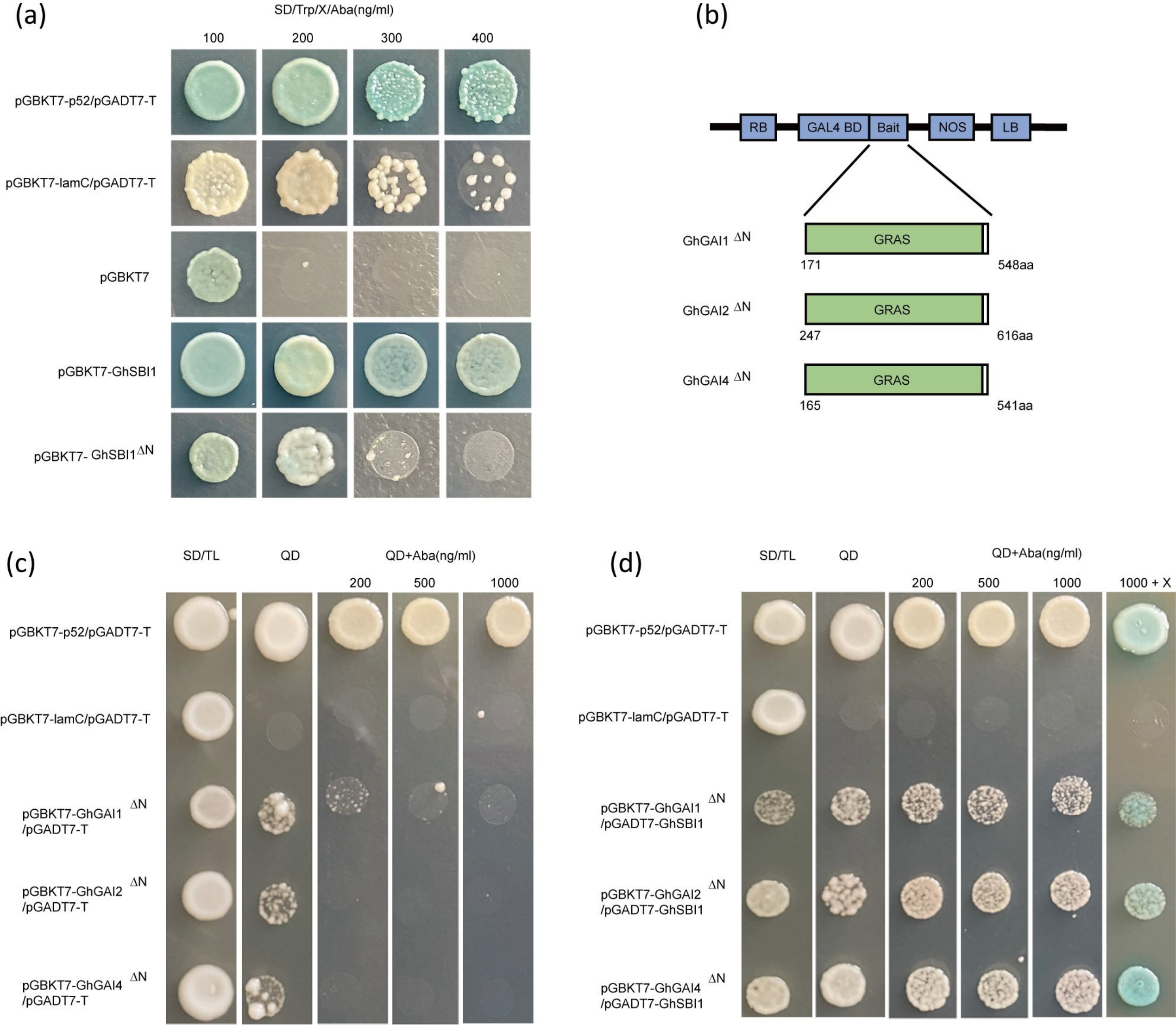

(a)
SD/Trp/X/Aba(ng/ml)
100
200
300
400
pGBKT7-p52/pGADT7-T
pGBKT7-lamC/pGADT7-T
pGBKT7
pGBKT7-GhSBI1
pGBKT7- GhSBI1 ΔN
(b)
RB
GAL4 BD
Bait
NOS
LB
GhGAI1 ΔN
GRAS
171
548aa
GhGAI2 ΔN
GRAS
247
616aa
GhGAI4 ΔN
GRAS
165
541aa
(c)
SD/TL
QD
QD+Aba(ng/ml)
200
500
1000
pGBKT7-p52/pGADT7-T
pGBKT7-lamC/pGADT7-T
pGBKT7-GhGAI1 ΔN /pGADT7-T
pGBKT7-GhGAI2 ΔN /pGADT7-T
pGBKT7-GhGAI4 ΔN /pGADT7-T
(d)
SD/TL
QD
QD+Aba(ng/ml)
200
500
1000
1000 + X
pGBKT7-p52/pGADT7-T
pGBKT7-lamC/pGADT7-T
pGBKT7-GhGAI1 ΔN /pGADT7-GhSBI1
pGBKT7-GhGAI2 ΔN /pGADT7-GhSBI1
pGBKT7-GhGAI4 ΔN /pGADT7-GhSBI1
