# Supplementary material for: GhSBI1, a CUP‐SHAPED COTYLEDON 2 homologue, modulates branch internode elongation in cotton
Source: Plant Biotechnol J. 2024 Jul 26;22(11):3175–93. doi: 10.1111/pbi.14439 (PMC11500989; doi:10.1111/pbi.14439)
Supplement: Supplementary file 4 [file PBI-22-3175-s002.docx]

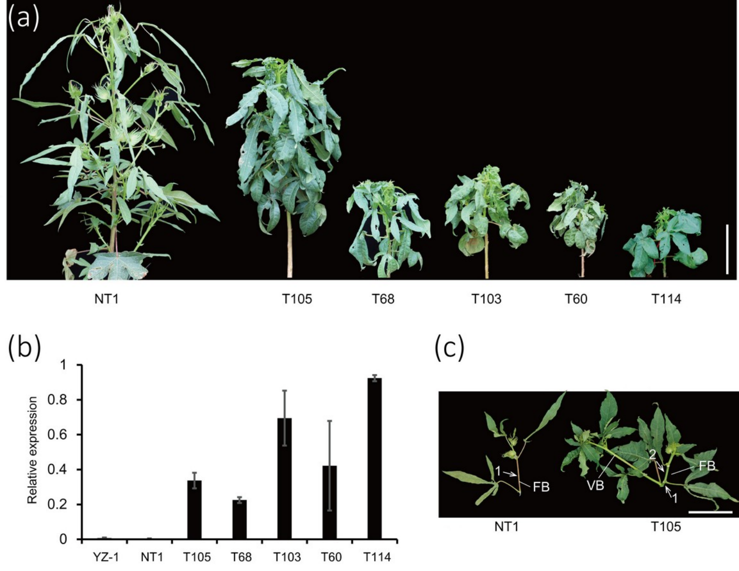
(a)
NT1
T105
T68
T103
T60
T114
(b)
Relative expression
0
0.2
0.4
0.6
0.8
1
YZ-1
NT1
T105
T68
T103
T60
T114
(c)
1
FB
VB
2
FB
1
NT1
T105
