# Supplementary material for: GhSBI1, a CUP‐SHAPED COTYLEDON 2 homologue, modulates branch internode elongation in cotton
Source: Plant Biotechnol J. 2024 Jul 26;22(11):3175–93. doi: 10.1111/pbi.14439 (PMC11500989; doi:10.1111/pbi.14439)
Supplement: Supplementary file 5 [file PBI-22-3175-s003.docx]

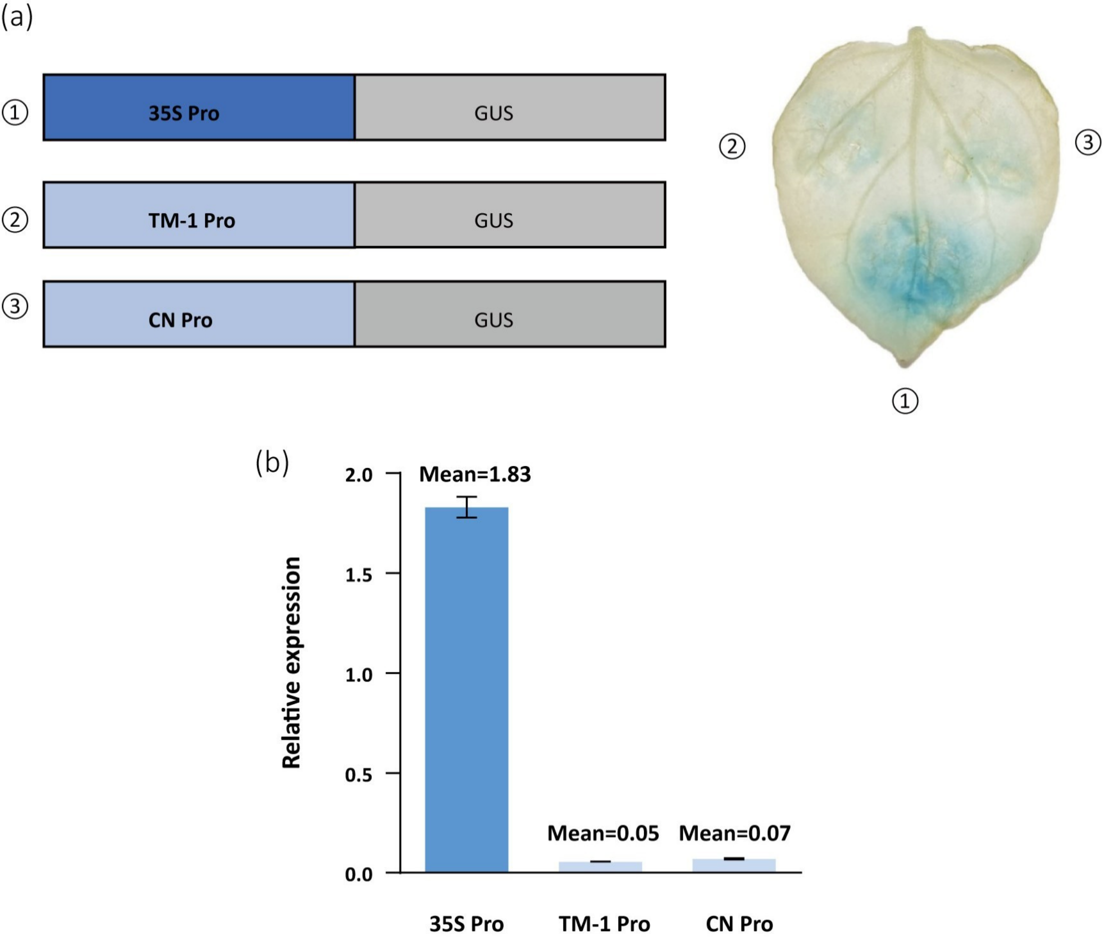
(a)
①
35S Pro
GUS
②
TM-1 Pro
GUS
③
CN Pro
GUS
②
③
①
(b)
Mean=1.83
Mean=0.05
Mean=0.07
Relative expression
2.0
1.5
1.0
0.5
0.0
35S Pro
TM-1 Pro
CN Pro
