# Supplementary material for: GhSBI1, a CUP‐SHAPED COTYLEDON 2 homologue, modulates branch internode elongation in cotton
Source: Plant Biotechnol J. 2024 Jul 26;22(11):3175–93. doi: 10.1111/pbi.14439 (PMC11500989; doi:10.1111/pbi.14439)

Motif 2: 5’-AAAATWTAKYTWCWTT-3’ E-value = 2.5e-020

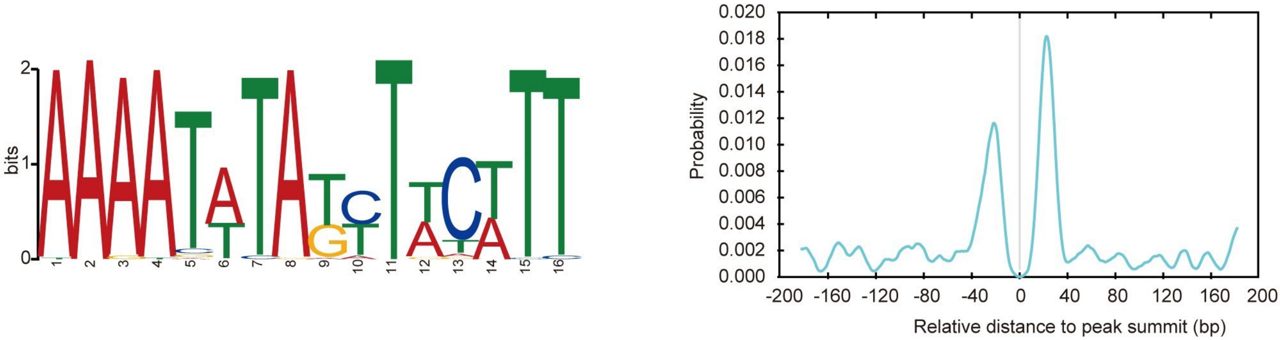

Motif 3: 5’-AGAMWTTGGRTYACACMA-3’ E-value = 8.7e-019

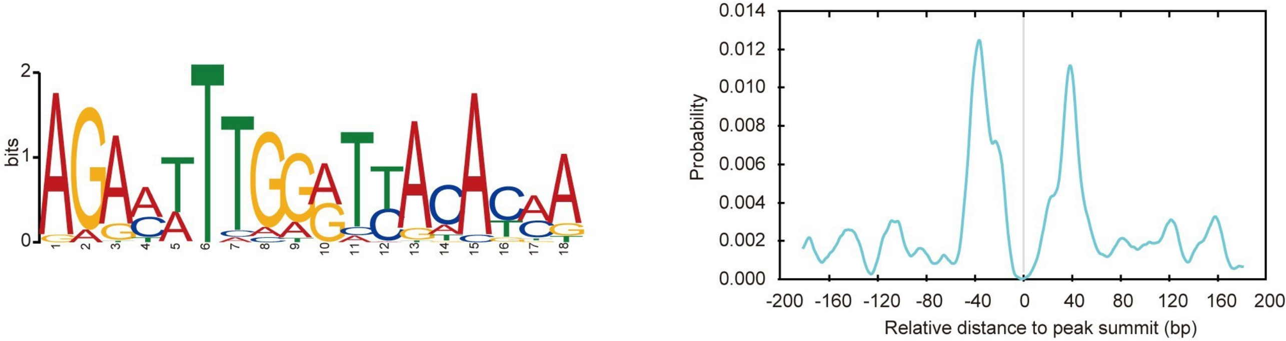

Motif 4: 5’-AGTCACTTTCTATAGTT-3’ E-value = 7.8e-015

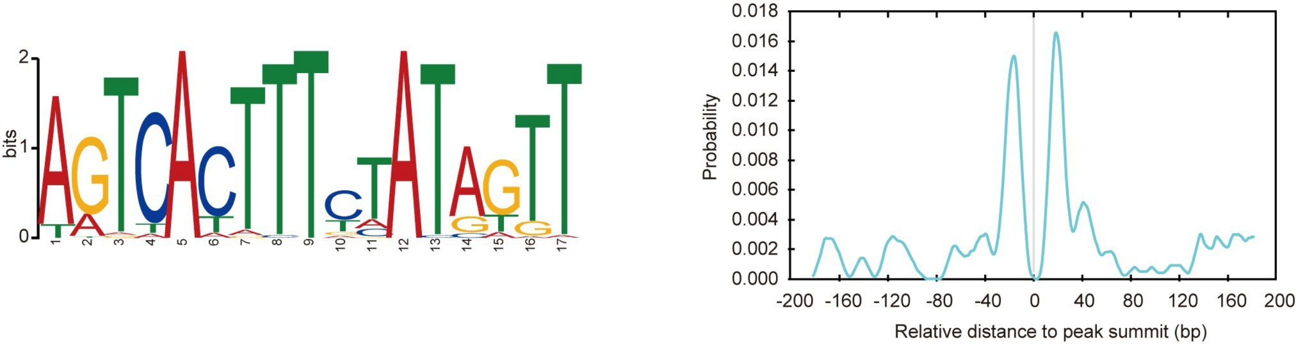

Supplement: Supplementary file 6 [file PBI-22-3175-s006.docx]
