# Supplementary material for: GhSBI1, a CUP‐SHAPED COTYLEDON 2 homologue, modulates branch internode elongation in cotton
Source: Plant Biotechnol J. 2024 Jul 26;22(11):3175–93. doi: 10.1111/pbi.14439 (PMC11500989; doi:10.1111/pbi.14439)
Supplement: Supplementary file 7 [file PBI-22-3175-s007.docx]

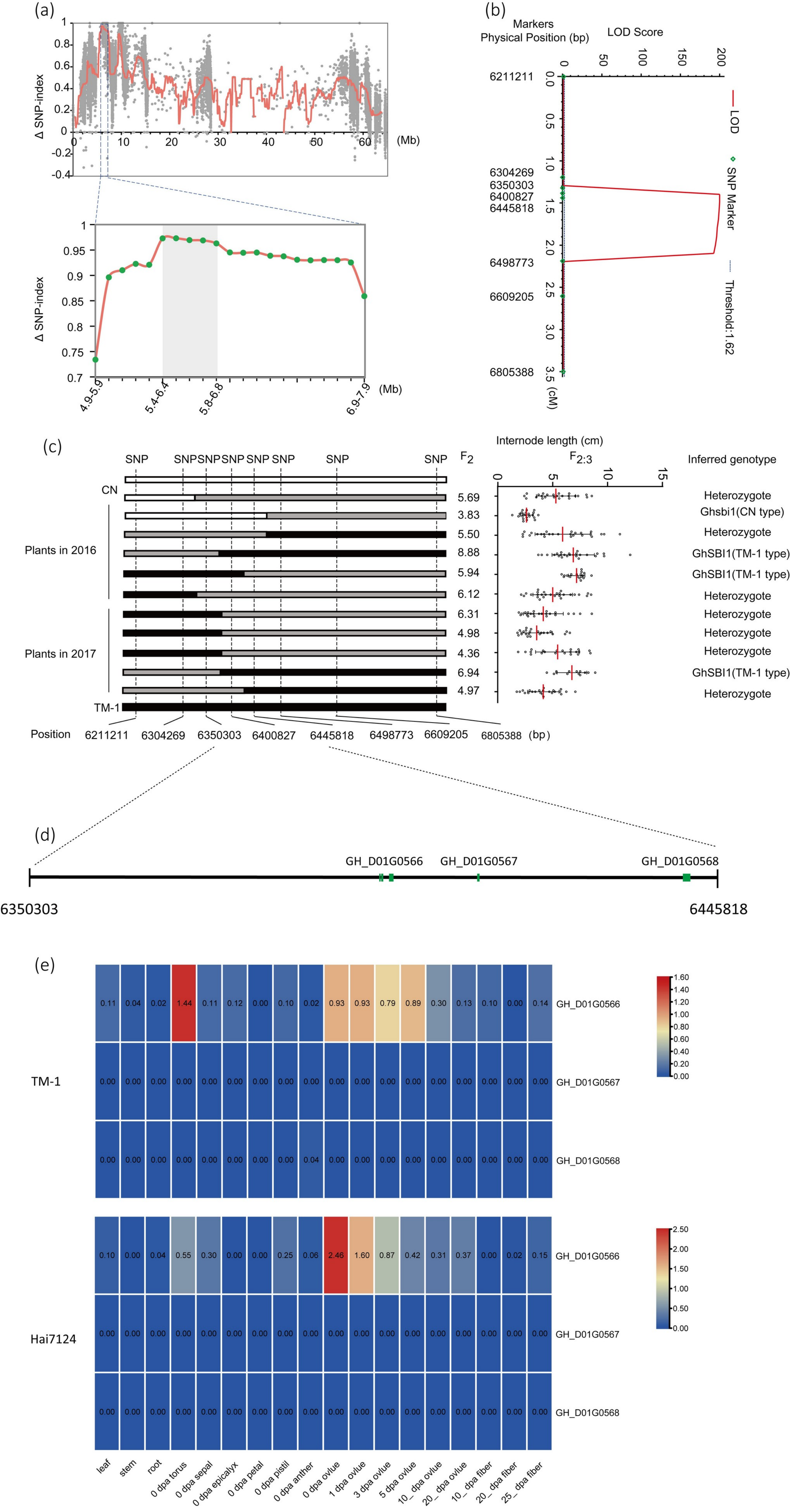

(a)
Δ SNP-index
(Mb)
4.9-5.9
5.4-6.4
5.8-6.8
6.9-7.9
(b)
Markers Physical Position (bp)
LOD Score
6211211
6304269
6350303
6400827
6445818
6498773
6609205
6805388
(cM)
LOD
SNP Marker
Threshold:1.62
(c)
SNP
CN
Plants in 2016
Plants in 2017
TM-1
Position
6211211 6304269 6350303 6400827 6445818 6498773 6609205 6805388 (bp)
F2
5.69
3.83
5.50
8.88
5.94
6.12
6.31
4.98
4.36
6.94
4.97
Internode length (cm)
F2:3
Inferred genotype
Heterozygote
Ghsbi1(CN type)
Heterozygote
GhSBI1(TM-1 type)
GhSBI1(TM-1 type)
Heterozygote
Heterozygote
Heterozygote
Heterozygote
GhSBI1(TM-1 type)
Heterozygote
(d)
GH_D01G0566
GH_D01G0567
GH_D01G0568
6350303
6445818
(e)
TM-1
0.11 0.04 0.02 1.44 0.11 0.12 0.00 0.10 0.02 0.93 0.93 0.79 0.89 0.30 0.13 0.10 0.00 0.14 GH_D01G0566
0.00 0.00 0.00 0.00 0.00 0.00 0.00 0.00 0.00 0.00 0.00 0.00 0.00 0.00 0.00 0.00 0.00 0.00 GH_D01G0567
0.00 0.00 0.00 0.00 0.00 0.00 0.00 0.00 0.04 0.00 0.00 0.00 0.00 0.00 0.00 0.00 0.00 0.00 GH_D01G0568
Hai7124
0.10 0.00 0.04 0.55 0.30 0.00 0.00 0.25 0.06 2.46 1.60 0.87 0.42 0.31 0.37 0.00 0.02 0.15 GH_D01G0566
0.00 0.00 0.00 0.00 0.00 0.00 0.00 0.00 0.00 0.00 0.00 0.00 0.00 0.00 0.00 0.00 0.00 0.00 GH_D01G0567
0.00 0.00 0.00 0.00 0.00 0.00 0.00 0.00 0.00 0.00 0.00 0.00 0.00 0.00 0.00 0.00 0.00 0.00 GH_D01G0568
leaf
stem
root
0 dpa torus
0 dpa sepal
0 dpa epicalyx
0 dpa petal
0 dpa pistil
0 dpa anther
0 dpa ovlue
1 dpa ovlue
3 dpa ovlue
5 dpa ovlue
10_ dpa ovlue
20_ dpa ovlue
10_dpa fiber
20_ dpa fiber
25_ dpa fiber
