# Supplementary material for: GhSBI1, a CUP‐SHAPED COTYLEDON 2 homologue, modulates branch internode elongation in cotton
Source: Plant Biotechnol J. 2024 Jul 26;22(11):3175–93. doi: 10.1111/pbi.14439 (PMC11500989; doi:10.1111/pbi.14439)
Supplement: Supplementary file 8 [file PBI-22-3175-s008.docx]

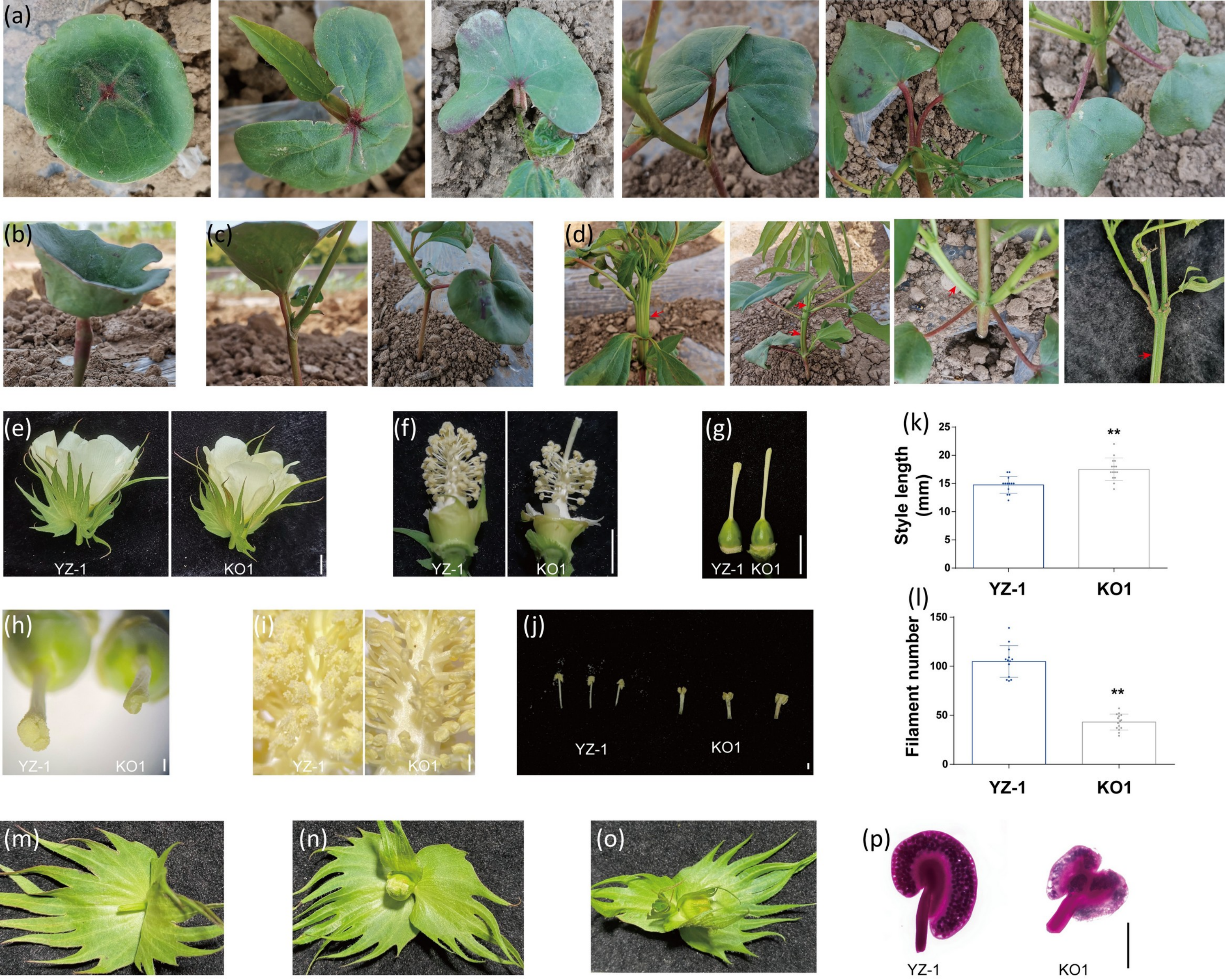

(a)
(b)
(c)
(d)
(e)
YZ-1
KO1
(f)
YZ-1
KO1
(g)
YZ-1 KO1
(h)
YZ-1
KO1
(i)
YZ-1
KO1
(j)
YZ-1
KO1
(k)
Style length (mm)
25
20
15
10
5
0
**
YZ-1
KO1
(l)
Filament number
150
100
50
0
**
YZ-1
KO1
(m)
(n)
(o)
(p)
YZ-1
KO1
