# Supplementary material for: GhSBI1, a CUP‐SHAPED COTYLEDON 2 homologue, modulates branch internode elongation in cotton
Source: Plant Biotechnol J. 2024 Jul 26;22(11):3175–93. doi: 10.1111/pbi.14439 (PMC11500989; doi:10.1111/pbi.14439)
Supplement: Supplementary file 9 [file PBI-22-3175-s009.docx]

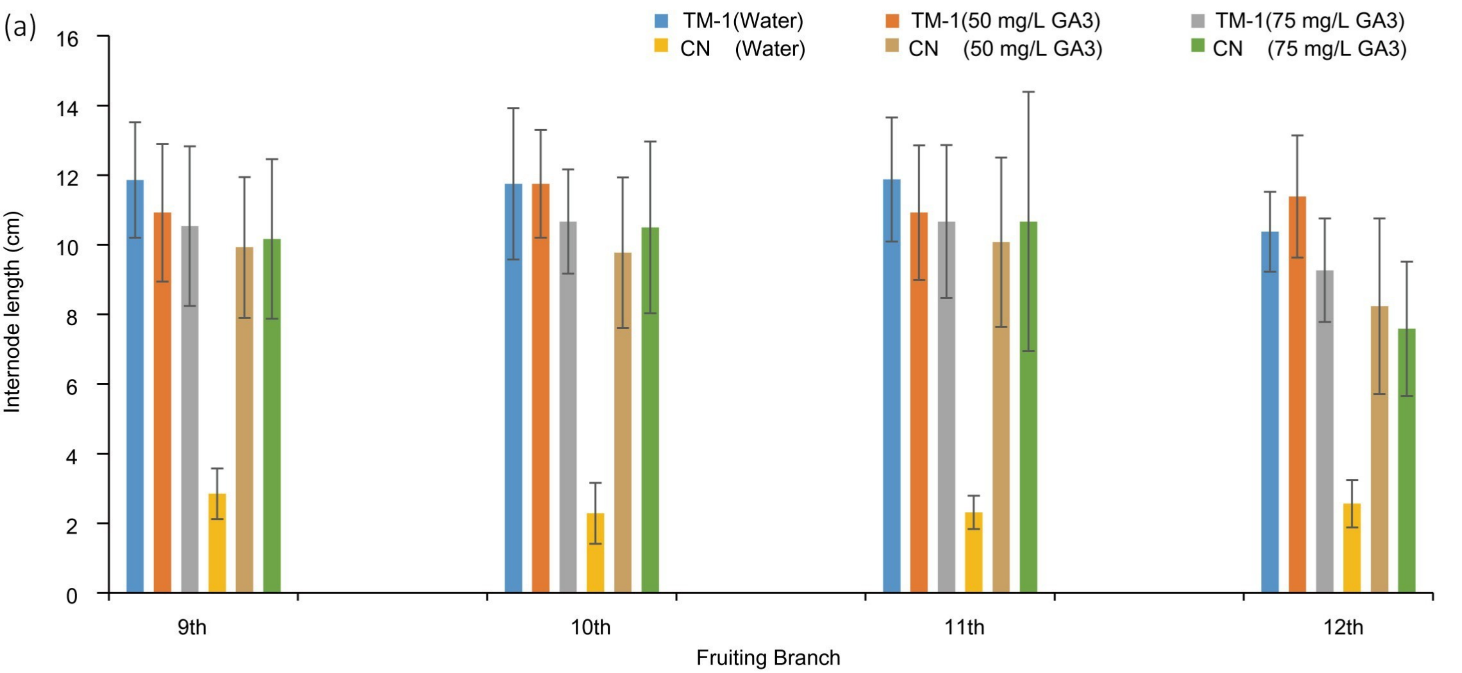
(a)
TM-1(Water)
TM-1(50 mg/L GA3)
TM-1(75 mg/L GA3)
CN (Water)
CN (50 mg/L GA3)
CN (75 mg/L GA3)
Internode length (cm)
0
2
4
6
8
10
12
14
16
9th
10th
11th
12th
Fruiting Branch

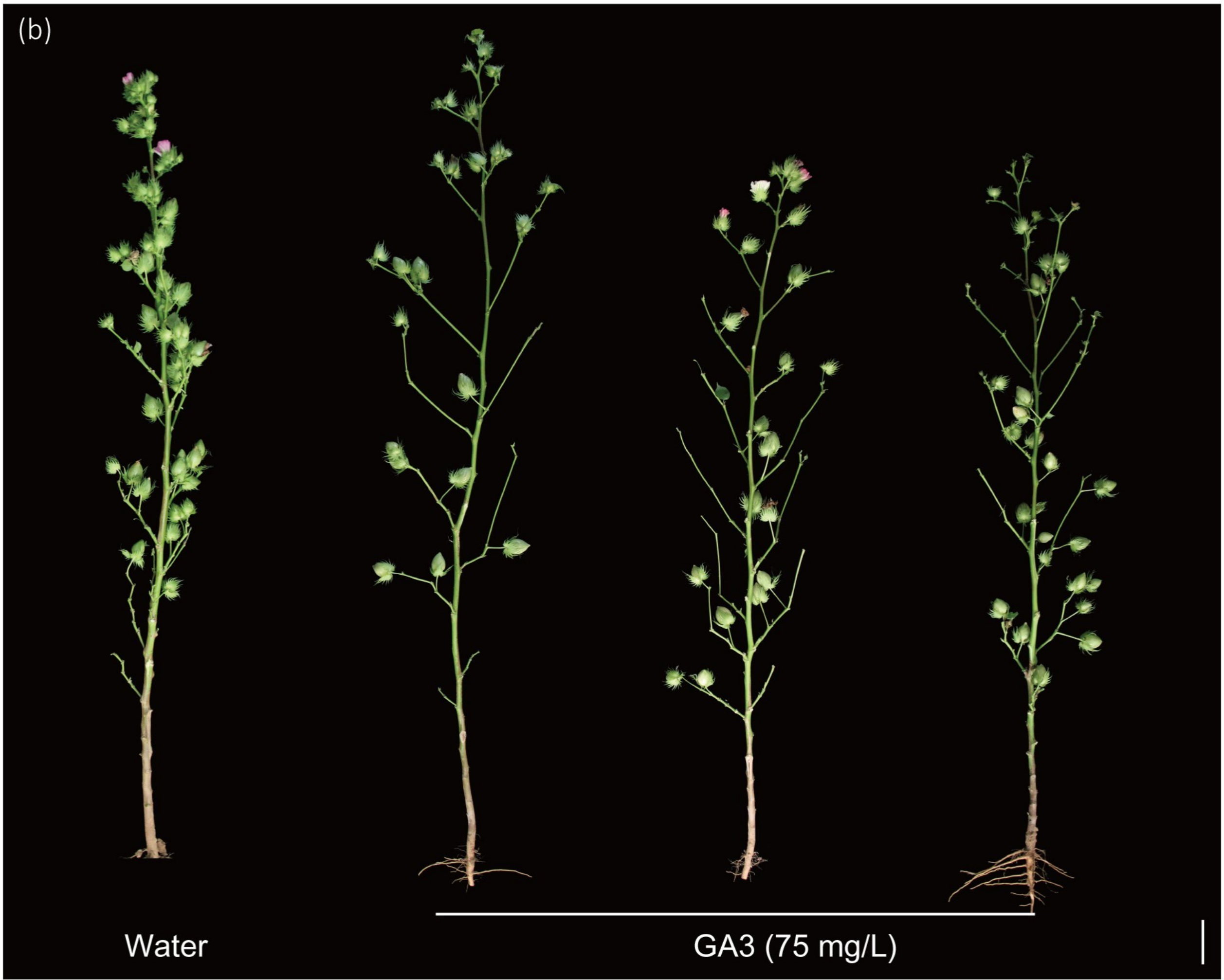
(b)
Water
GA3 (75 mg/L)
