# Supplementary material for: GhSBI1, a CUP‐SHAPED COTYLEDON 2 homologue, modulates branch internode elongation in cotton
Source: Plant Biotechnol J. 2024 Jul 26;22(11):3175–93. doi: 10.1111/pbi.14439 (PMC11500989; doi:10.1111/pbi.14439)
Supplement: Supplementary file 10 [file PBI-22-3175-s010.docx]

(a)

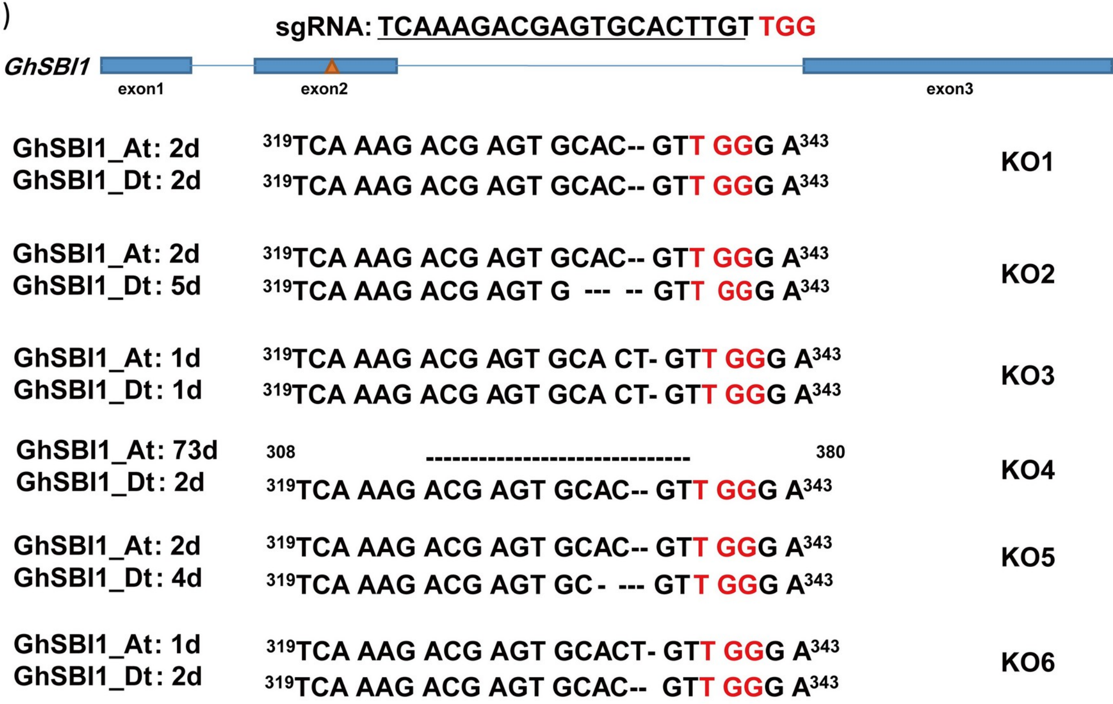

(b)

Lines	Fully fused (Cup-shaped)	Partially fused	Unfused	Ratio of fused cotyledons
KO1	3	12	22	0.41
KO2	1	29	15	0.67
KO3	0	9	11	0.45
KO4	0	9	12	0.43
KO5	1	25	35	0.43
KO6	1	13	27	0.34
